# Green-Emitting 4,5-Diaminonaphthalimides in Activity-Based Probes for the Detection of Thrombin

**DOI:** 10.1021/acs.orglett.2c02320

**Published:** 2022-07-21

**Authors:** Maciej Krzeszewski, Sylwia Modrzycka, Manon H. E. Bousquet, Denis Jacquemin, Marcin Drąg, Daniel T. Gryko

**Affiliations:** † Institute of Organic Chemistry, Polish Academy of Sciences, Kasprzaka 44/52, Warsaw 01-224, Poland; ‡ Department of Chemical Biology and Bioimaging, Wroclaw University of Science and Technology, Wybrezeże Wyspiańskiego 27, Wrocław 50-370, Poland; § CEISAM UMR CNRS 6230, Nantes University, Nantes 44000, France

## Abstract

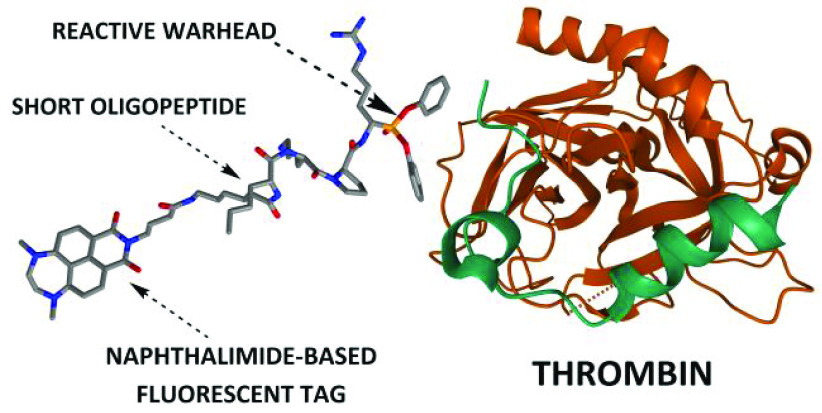

The natures of electron-donating groups as well as the bridge between them determine the fate of substituted 1,8-naphthalimide molecules in the excited state. An activity-based probe constructed from a selective peptide sequence, a reactive warhead, and the brightest green-emitting fluorophore displays impressive performance for thrombin protease detection in a newly constructed series of 1,8-naphthalimides.

Many aspects of modern molecular biology and medicine depend on fluorescence imaging modalities to gather key information about the functions of biological systems and to develop efficient diagnostic tools or even tools for imaging during surgery. Currently, one of the hottest areas of development regarding new imaging systems is the visualization of enzyme activity, particularly for enzymes that are involved in the development of diseases. One of the most extensively studied proteolytic enzyme is thrombin, a serine protease from the common coagulation cascade pathway.^1^ Beyond maintaining the balance between coagulation and blood circulation, thrombin plays an important role in inflammation and is involved in viral infections, angiogenesis, and fibrosis.^2,3^ The abnormal activity of this protease has been associated with numerous disorders, such as hemophilia,^4^ thrombosis,^5^ stroke,^6^ multiple sclerosis,^7^ cancer progression,^8^ and Alzheimer’s disease.^9,10^ Furthermore, recent studies have shown that thrombin inhibitors can be used to treat COVID-19 patients.^3^ The ability to monitor and detect thrombin activity would improve our understanding of thrombin’s role in both physiological and pathophysiological conditions. Therefore, the aim of our study was to develop chemical tools with new fluorescent tags for the simple and straightforward detection of thrombin. To achieve this goal, molecules called activity-based probes (ABPs) are used. The power of ABPs has been demonstrated in several crucial biological and pathological processes such as apoptosis, cancer metastasis, and malaria infection, and current approaches are even related to cancer imaging *in vivo* during surgery.^11−13^ Along these lines, the construction of rapid and effective diagnostic tests involves the rational design of a fluorescent probe anchored to a potent inhibitor in the form of a short oligopeptide.^14^ A suitable fluorescent reporter for this purpose should exhibit superb photostability and high brightness (defined as a product of the molar extinction coefficient and the fluorescence quantum yield, ε × Φ_F_) while simultaneously maintaining a small molecular size so that it does not interfere with the protease–oligopeptide recognition process. One of the most favorable chromophores that meets these requirements is 1,8-naphthalimide (NI). 1,8-Naphthalimides are conveniently modifiable, and their photophysical properties are easily tunable.^15−20^ Due to their versatility, NIs have been extensively studied and have found applications in many fields, including the fabrication of organic light-emitting diodes (OLEDs),^21^ organic solar cells,^22^ memory devices,^23,24^ and chemosensors^25^ and as tags in fluorescence-labeled probes for cellular imaging.^26^ In this article we present our investigation of emissive 1,8-naphthalimide derivatives as potential fluorescent tags that can be used in the synthesis of activity-based probes for thrombin detection. Our molecular design strategy toward potentially highly emissive fluorophores involved the synthesis of a library of 1,8-naphthalimides bearing various nitrogen-based substituents located at positions 4 and 5 of the core opposite the imide functionality. We prepared NIs with various electron-donating groups—the core of the dye acted as the electron-accepting moiety—to investigate the impact of the electronic character of the substituents on the overall absorption and emission features. The installation of electron-donating groups on the electron-deficient 1,8-naphthalimide core indeed leads to a donor−π–acceptor (D−π–*A*) dipolar architecture. Such push–pull systems involve the formation of an intramolecular charge-transfer (ICT) excited state and result in significant bathochromic shifts in both absorption and emission spectra.^27^

We began with the preparation of the subsituted parent 1,8-naphthalimides **2a**–**d**, namely, *N*-octyl-4,5-dibromo-1,8-naphthalimide, *N*-butyl-4,5-dibromo-1,8-naphthalimide, *N*-butyl-4-nitro-1,8-naphthalimide, and *N*-butyl-4-bromo-5-nitro-1,8-naphthalimide, respectively (Figure 1a), which were easily obtained from the corresponding anhydrides **1a**–**c** (see Scheme S1). Then, **2a**–**d** were subjected to three different synthetic strategies. The first approach involved an aromatic nucleophilic substitution (S_N_Ar) reaction. In the reaction of **2a** with guanidine hydrochloride in DMF at 110 °C for 4 h, dye **3a** was formed in a 48% yield.^28^ In the reactions of **2a** and **2b** with dimethylamine hydrochloride, 1,2-dimethylethylenediamine (DMEDA), *n-*butylamine, and azetidine in DMSO at 120 °C for 16 h in the presence of triethylamine, **3b**–**e** were furnished, respectively.^15,29^ Another approach was applied for dye **3f** that involved the reduction of the nitro group of **2c** followed by the Paal–Knorr synthesis of the pyrrole, which gave the product in a yield of 78% over two steps.^30^ The dye **3g** was obtained from **2d**, bearing both nitro and bromo functional groups, in a manner analogous to that of **3f**, with an additional final step of a Pd-catalyzed intramolecular direct arylation reaction.^31−33^ The final yield reached 59% over three steps (Figure 1a). For the reaction of **2b** with azetidine under the given conditions, it should be noted the transformation took place only on one side of the molecule, leaving the second bromine atom intact and providing **3e** with a yield of a 72%. Furthermore, in the reaction of **2a** with dimethylamine hydrochloride, the expected product was not observed; instead, the final compound **3b**, which formed in an 86% yield, contained only one attached methylamino group (a product of simultaneous demethylation and debromination). The photophysical properties of this library of seven strongly polarized 1,8-naphthalimides were investigated (Figure 1b and Figures S1−S7). The preliminary results suggested that dyes **3a** and **3c** were the most promising candidates for further biological studies, as they possessed the highest brightnesses with values of 28900 and 27200 M^–1^ cm^–1^, respectively. Interestingly the emission of imide **3c** in ethanol was hypsochromically shifted *ca*. 40 nm compared to that of the 4-amino-1,8-naphthalimides. In contrast, the installation of the pyrrole moiety in the chromophore had a detrimental effect on the Φ_F_ values, as shown by **3f** and **3g** (a similar effect was observed earlier for a regioisomeric pyrrole-fused NI).^34^ The photophysical outcomes were rationalized by theoretical calculations (*vide infra*). Having gained this knowledge, we designed and synthesized analogous fluorophores carrying a carboxylic functional group that was attached to the main core through a short alkyl chain (**4a** and **4b**; Figure 1c and Scheme S5). Along these lines 1,8-naphthalimide **2e**, which contained a short chain of *tert*-butyl butyrate, was prepared.^35^ The dyes **3h** and **3i** were synthesized analogously to **3c** and **3a**, respectively. The *tert*-butyl ester group was easily hydrolyzed by trifluoroacetic acid (TFA), yielding **4a** and **4b**. On the basis of the structure of the previously obtained thrombin-selective substrate^36^ we designed two fluorescent activity-based probes, namely, **5a** and **5b**, that contained fluorophores **4a** and **4b**, respectively, and one reference biotin-labeled probe **5c** that contained a commercially available biotin tag. First, using the solid-phase approach, a recognition element was synthesized by elongating the peptide on 2-chlorotrityl resin. Then, the tags were separated from the substrate sequence with a 6-aminohexanoic acid linker, which was applied to reduce the steric hindrance. We selected a diphenyl phosphonate, an electrophile known to covalently bind to the active site of serine proteases,^37^ as a reactive warhead. The warhead was obtained according to a previously described methodology^38^ and coupled in a solution to the peptide sequence using coupling reagents to give the final activity-based probes **5a**–**c** (see the Supporting Information for details). The mechanism of thrombin inhibition by the activity-based probe is presented in the Supporting Information (Scheme S6). The very different photophysical properties of the structurally similar dyes **3a**–**3g** puzzled us, and we decided to perform theoretical calculations, which are detailed in the Supporting Information. In Figure 2 we report density difference plots for **3a**, **3b**, and **3g**. It can be seen that while ICT is rather mild in the former dye it is significant in **3b** and large in **3g**, which qualitatively explains the small experimental Stokes shift in **3a** (400 cm^–1^) and the significantly higher values in **3b** (3800 cm^–1^) and **3g** (5700 cm^–1^). Note that for the latter dye the theoretical calculation corresponds to the lowest excited state, which yields the moderately intense red-shifted band at 465 nm; the second excited state, which presents a much larger oscillator strength, corresponds to the more intense 418 nm band.

**Figure 1:**
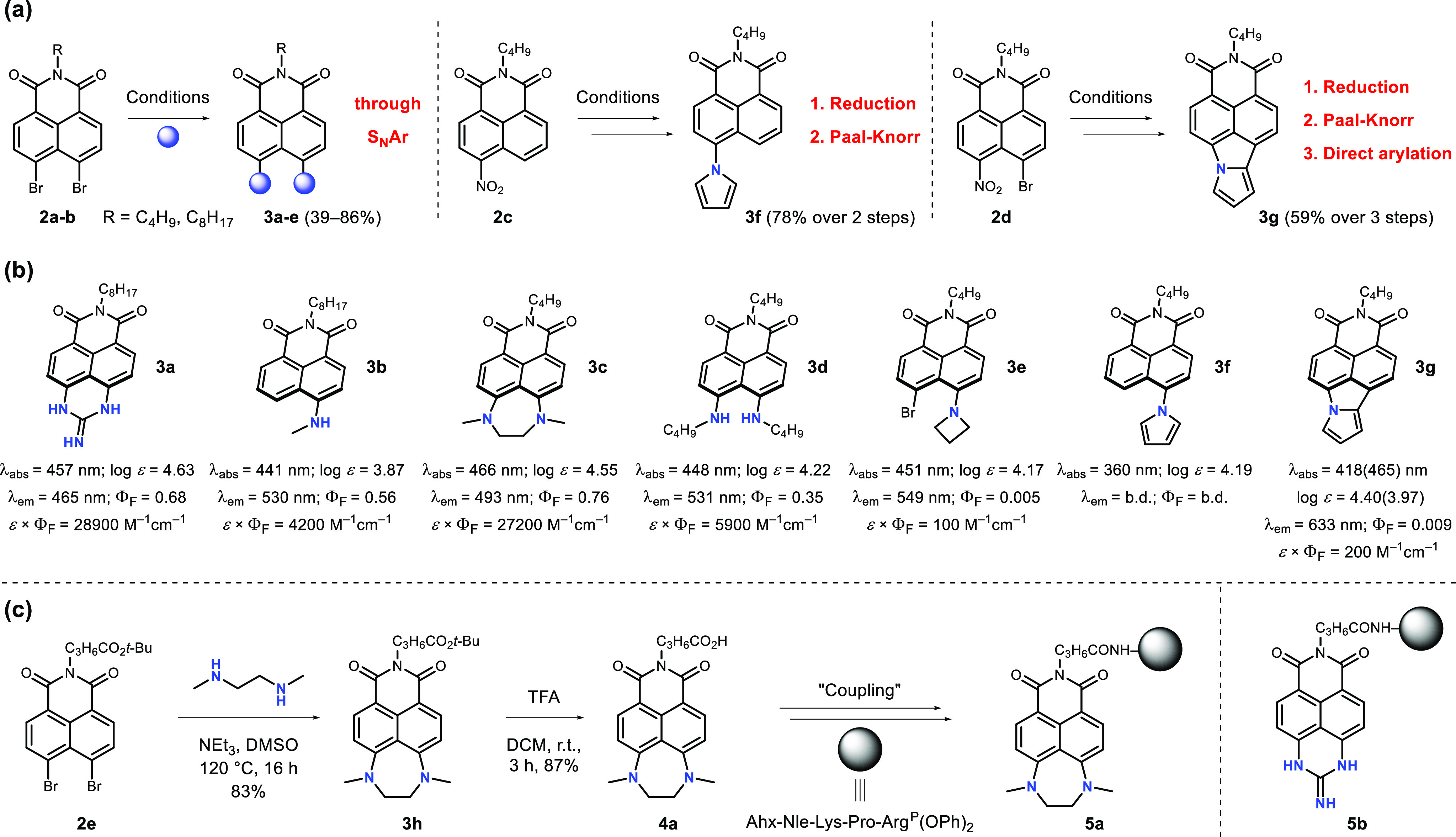
Summary of the synthetic part of this study. (a) Three general synthetic strategies utilized in the preparation of **3a**–**g**. (b) Preliminary screening of the synthesized dyes for desirable photophysical properties. All measurements were performed in ethanol, and the fluorescence quantum yield (Φ_F_) was determined with Coumarin 153 (Φ_F_ = 0.544) as a standard; b.d. = below the detection limit. (c) An example synthetic pathway toward a selected activity-based probe **5a** for thrombin detection. The probe **5b** was synthesized in an analogous manner (see Scheme S5).

**Figure 2:**
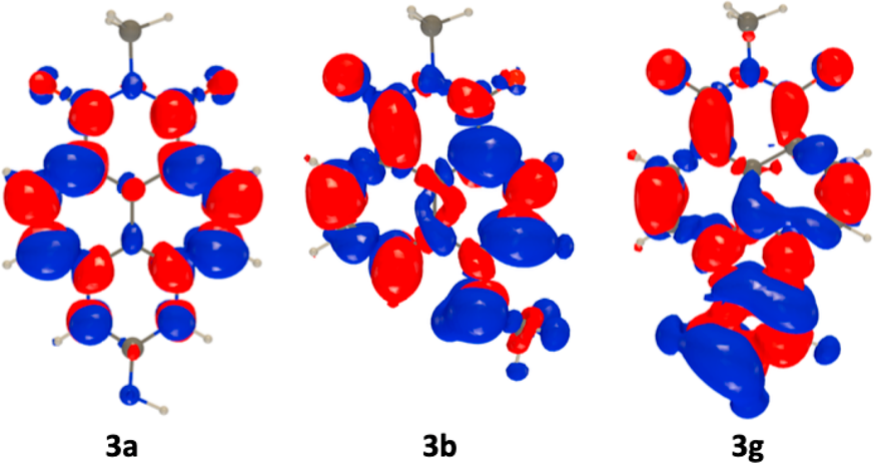
Density difference plot of selected dyes. The blue and red lobes correspond to regions of decreasing and increasing electron density upon excitation, respectively. The contour threshold was 1 × 10^–3^.

Next, we turned our attention to more quantitative comparisons with the photophysical signatures. Calculations show that twisted intramolecular charge transfer (TICT), which leads to a dark (nonemissive) state, is an almost barrierless process in **3f** and has a low barrier in **3e** (Table 1). For those two dyes, the quenching of the emission is therefore due to TICT. In addition, in **3e**, the intersystem crossing likely plays a role in lowering the emission yield, as the spin–orbit coupling is noticeably larger than those in all other compounds (see Table S2). In **3d**, two groups can undergo TICT. The rotation of the first group is a barrierless process that leads to a bright excited state with a strong intramolecular H-bond between the amino groups; this makes the second TICT less accessible (barrier of ca. 0.5 eV, see Figure S10) but probably not beyond reach due to the large vibrational energy present in the excited state. In any case, there is a large geometry reorganization in the excited state of **3d** (first TICT), which is consistent with the dissymmetric absorption and emission band shapes.

For **3a**, **3b**, **3c**, and **3g**, the calculations indicate the absence of both TICT and ISC. In such a scenario, the remaining competitive excited state processes are likely fluorescence (radiative) and internal conversion (nonradiative). The fact that the *ab initio* determined Φ_F_ values of 0.77, 0.40, 0.66, and 0.08 for **3a**, **3b**, **3c**, and **3g**, respectively, are in agreement with the experimental values of 0.68, 0.56, 0.76, and 0.01 reported in Figure 1 confirms that no parasitic nonradiative pathway is active in those four compounds. As can be seen in the Supporting Information (Figures S11, S12), there is also a good agreement between experimental and theoretical band shapes for all cases. The notably lower fluorescence yield of **3g** is due to a combination of a relatively small radiative constant (small *k*_r_) and a large IC rate (large *k*_nr_), which are respectively related to a small oscillator strength and very red-shifted emission. Finally, for **3d**, theoretical calculations yield a Φ_F_ value that is too large (0.61 versus 0.36); we take this as an indication that the second TICT process actually competes with radiative decay, which might explain the lower measured yield.

**Table 1:** Comparison between Experimental and Theoretical 0-0 Energies (eV), log(ε) Values, and Emission Yields for the Five Dyes Not Quenched by TICT^a^

	0–0 (eV)		log(ε)		Φ_F_	
dye	theor	exptl	theor	exptl	theor	exptl
**3a**	2.96	2.69	4.48	4.63	0.77	0.68
**3b**	2.65	2.58	4.27	3.87	0.40	0.56
**3c**	2.68	2.59	4.57	4.55	0.66	0.76
**3d**	2.65	2.50	4.55	4.22	0.61	0.35
**3g**	2.42	2.31	3.78	3.97	0.08	0.01

a The yields consider only the radiative and internal conversion processes. For **3d**, the emissive properties were determined from the bright single-TICT structure. See the Supporting Information for details.

The initial goal was to develop chemical tools with novel fluorescent tags capable of detecting active proteases. Our activity-based probes are equipped with diphenyl phosphonate as a reactive group; therefore, they also have the properties of a covalent inhibitor. Since we introduced a modification in the structure of the reference biotinylated probe **5c** (the *N*-terminus biotin was changed to two new fluorescent tags), we had to verify whether the inhibition potencies of our **5a** and **5b** probes were maintained. Using a fluorogenic tetrapeptide substrate designed for thrombin, we tested the residual activity of thrombin that was separately preinhibited with each probe at various probe concentrations ranging from 1 to 1000 nM. As indicated in Figure 3, we can see the minimal inhibition of thrombin with probe concentrations of 1 and 10 nM. However, at 10-fold probe excess (100 nM), both compounds with new fluorophores inhibited thrombin activity more effectively than the reference biotinylated probe, indicating that tag replacement did not negatively interfere with the protease-activity-based probe recognition process. The activity of thrombin was completely blocked when the concentration of all the activity-based probes reached 1000 nM.

**Figure 3:**
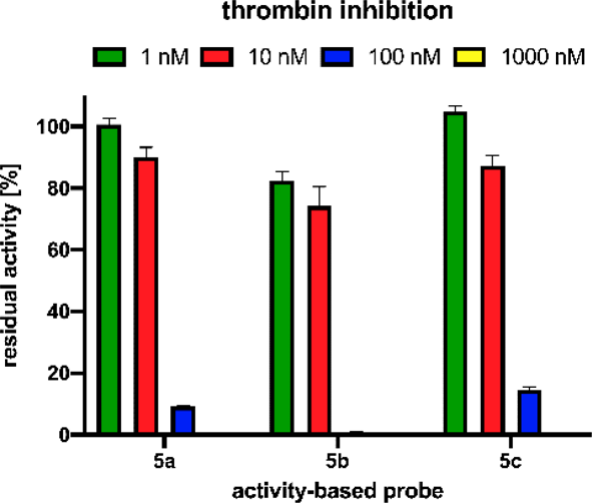
Inhibition of thrombin by biotinylated and fluorescent activity-based probes. The enzyme (10 nM) was incubated with various probe concentrations ranging from 1 to 1000 nM for 30 min at 37 °C. The appropriate substrate (100 μM) was then added to the reaction mixture, and the hydrolysis was measured for 30 min (λ_ex_ = 355 nm and λ_em_ = 460 nm). Data represent the mean values ± s.d.; *n* = 3, where *n* is the number of independent experiments.

To date, the *in situ* study of thrombin relies on antibody-related techniques that allow the evaluation of the total amount of enzyme present in a sample but discriminate poorly between the active and inactive forms (e.g., zymogen and inhibited protease). Since the presence of the active protein defines its function, there is a need for sensitive chemical tools able to detect the level of active thrombin in biological samples. Biotinylated activity-based probes are not suitable for the direct detection of protease activity; therefore, we next tested the utility of our fluorescent probes for the convenient and straightforward in-gel detection of active thrombin in test samples (Figure 4). We incubated purified thrombin with probe **5a** at concentrations ranging from 25 to 1200 nM for 30 min. As controls, we used the probe and enzyme alone.

**Figure 4:**
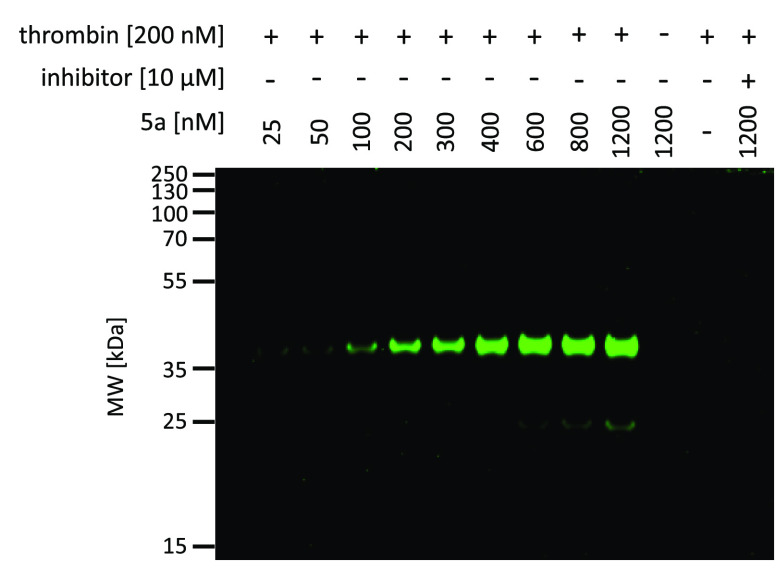
Labeling of purified thrombin using the fluorescent activity-based probe **5a**. The enzyme (200 nM) was incubated with various probe concentrations ranging from 25 to 1200 nM for 30 min at 37 °C. Additionally, thrombin (200 nM) was incubated with its inhibitor (final inhibitor concentration 10 μM) for 60 min prior to probe addition. The samples were then subjected to SDS-PAGE analysis and detected at 488 nm using an Azure Biosystems Sapphire Biomolecular Imager. The results are representative of at least three replicates.

One sample of thrombin was also preinhibited with 10 μM thrombin inhibitor for 60 min prior to probe addition. Then, a simple SDS-PAGE analysis was carried out to detect labeled thrombin. The probe bound to the enzyme, as indicated by the fluorescent signal from a protein between 35 and 55 kDa, which corresponds to the size of thrombin. The detection limit, that is, the minimum concentration of **5a** needed for thrombin labeling, was estimated to be approximately 100 nM. However, the optimal concentration was 600 nM. We did not observe an increase in fluorescence with a higher probe concentration; therefore, we concluded that thrombin was maximally labeled with the probe. Additionally, preblocking the active site of thrombin with the inhibitor prevented probe biding and completely inhibited the fluorescent signal. Therefore, we concluded that our activity-based probe selectively bound within the active site, supporting the utility of **5a** for the detection of thrombin.

In conclusion, nucleophilic aromatic substitution enables the straightforward synthesis of 1,8-naphthalimides possessing one or two electron-donating groups in positions 4 and 5. In the case of 4,5-diamino-NIs, the emission is markedly hypsochromically shifted compared to those of classic 4-amino-1,8-naphthalimides, but the brightness is higher. The use of probes containing these newly designed green-emitting fluorophores in thrombin detection revealed their superior performance, which supports their use in the design of activity-based probes for protease detection.

## Supporting Information Available

Experimental details and procedures, photophysical properties, biological studies, and spectral data of all new compounds (PDF)
