# Supplementary material for: Green-Emitting 4,5-Diaminonaphthalimides in Activity-Based Probes for the Detection of Thrombin
Source: Org Lett. 2022 Jul 21;24(30):5602–7. doi: 10.1021/acs.orglett.2c02320 (PMC9361357; doi:10.1021/acs.orglett.2c02320)
Supplement: Supplementary file 1 — ol2c02320_si_001.pdf [file ol2c02320_si_001.pdf]

Supporting Information

Green-Emitting 4,5-Diaminonaphthalimides in Activity-based Probes for the Detection of Thrombin

Maciej Krzeszewski,[a] Sylwia Modrzycka,[b] Manon H. E. Bousquet,[c] Denis Jacquemin,*[c] Marcin Drąg*[b] and Daniel T. Gryko *[a]

[a] Institute of Organic Chemistry, Polish Academy of Sciences, Kasprzaka 44/52, 01-224 Warsaw, Poland.
[b] Department of Chemical Biology and Bioimaging, Wroclaw University of Science and Technology, Wyb. Wyspianskiego 27, 50-370 Wroclaw, Poland
[c] CEISAM UMR 6230, CNRS, Université de Nantes, 44000 Nantes, France

Table of contents

1. General remarks....S2
2. Experimental section....S3
3. Photophysical properties....S12
4. Theoretical investigation....S16
5. Biological studies....S22
6. ^{1}H and ^{13}C NMR spectra for synthesized compounds....S28
7. References....S65

1. General remarks

All reagents and solvents were purchased from commercial sources and were used as received unless otherwise noted. 4,5-Dibromo-1,8-naphthalic anhydride (**1a**) was prepared according to the literature procedure[1] 4-nitro-1,8-naphthalic anhydride (**1b**) was purchased from Aldrich and 4-bromo-5-nitro-1,8-naphthalic anhydride (**1c**) was prepared according to the literature procedure.[2] *Tert*-butyl 4-aminobutyrate was prepared according to the literature procedure.[3] [1,4-Bis(diphenylphosphino)butane](η3-allyl)palladium (II) chloride ($PdCl(C_3H_5)$(dppb)) was prepared according to the literature procedure.[4] Reagent grade solvents (CH_2Cl_2, hexanes) were distilled prior to use. DMF was dried over magnesium sulfate, then distilled and stored under argon. For water-sensitive reactions solvents were dried using Solvent Purification System from MBraun (https://www.mbraun.com/us/). Transformations with moisture and oxygen sensitive compounds were performed under a stream of argon. The reaction progress was monitored by means of thin layer chromatography (TLC), which was performed on aluminium foil plates, covered with Silica gel 60 F_{254} (Merck) or Aluminium oxide 60 F_{254} (neutral, Merck). Products purification was done by means of column chromatography with Kieselgel 60 (Merck) or Aluminium oxide (Fluka). Occasionally, dry column vacuum chromatography (DCVC**)** for purification of products obtained was performed using Silica gel Type D 5F. The identity and purity of prepared compounds were proved by ^{1}H NMR and ^{13}C NMR spectrometry as well as by MS-spectrometry (*via* APCI-MS or ESI-MS). NMR spectra were measured on Bruker AM 500 MHz, Bruker AM 600 MHz, Varian 600 MHz, Varian 400 MHz or Varian 200 MHz instruments with TMS as internal standard. Chemical shifts for ^{1}H NMR are expressed in parts per million (ppm) relative to tetramethylsilane (δ 0.00 ppm), $CDCl_3$ (δ 7.26 ppm) or [D_6]-DMSO (δ 2.50 ppm). Chemical shifts for ^{13}C NMR are expressed in ppm relative to $CDCl_3$ (δ 77.16 ppm) or [D_6]-DMSO (δ 39.52 ppm). Data are reported as follows: chemical shift, multiplicity (s = singlet, d = doublet, dd = doublet of doublets, ddd = doublet of doublet of doublets, t = triplet, td = triplet of doublets, q = quartet, quint = quintet, sex = sextet, m = multiplet), coupling constant (Hz), and integration. All melting points for crystalline products were measured with automated melting point apparatus EZ-MELT and were given without correction. A Shimadzu UV-3600i Plus spectrophotometer and an Edinburgh Instruments Spectrofluorometer FS5 equipped with Hamamatsu R13456 PMT were used to acquire the absorption and emission spectra. Spectrophotometric grade solvents were used without further purification.

Linear optical measurements

All photophysical studies have been performed with freshly-prepared, air equilibrated solutions at room temperature (298 K). Steady-state fluorescence measurements were performed with dilute solutions (10^{-6} M, optical density < 0.1) contained in standard 1 cm quartz cuvettes. Compounds were dissolved in ethanol (EtOH) unless otherwise noted. Emission spectra of all the compounds were obtained under excitation at λ = 430 nm. Fluorescence quantum yields (Φ_F) were determined by using Coumarin 153 in ethanol (Φ_F = 0.554) as a standard.

2. Experimental section

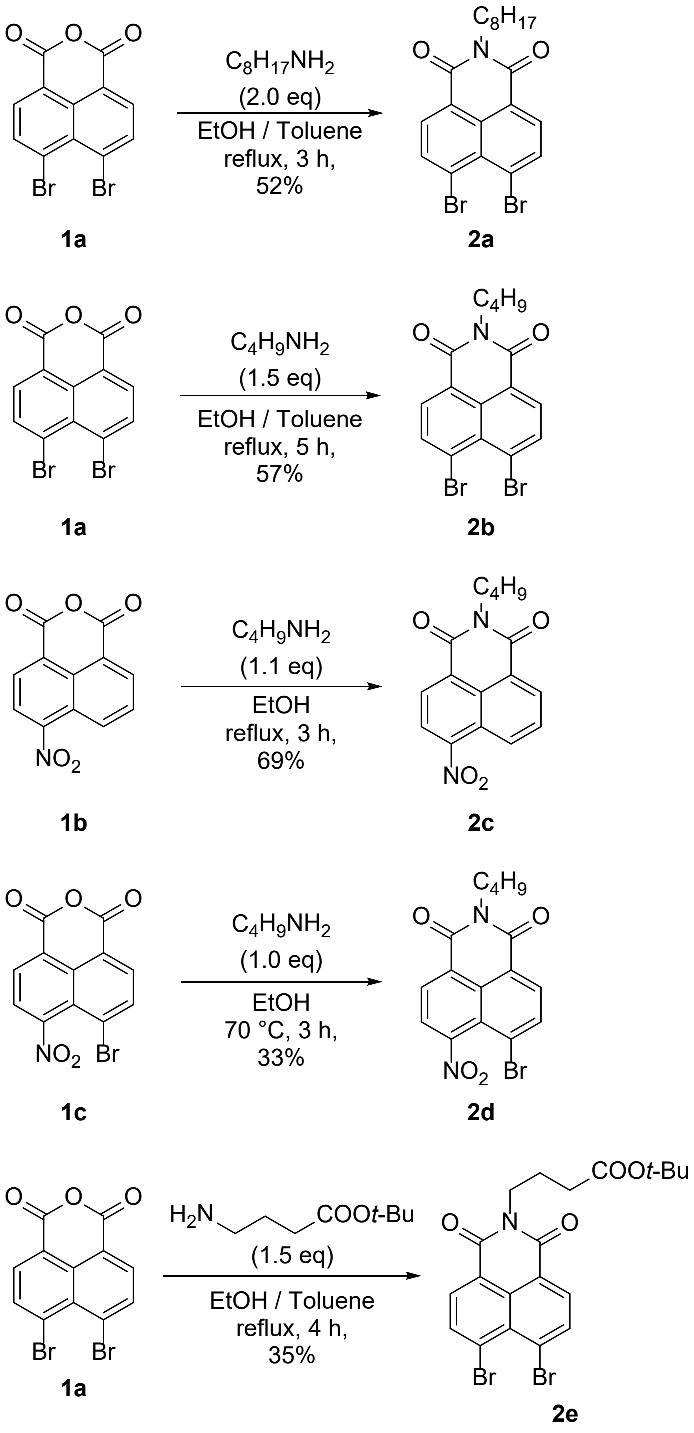

Scheme S1. Preparation of parent 1,8-naphthalimides **2a-e**.

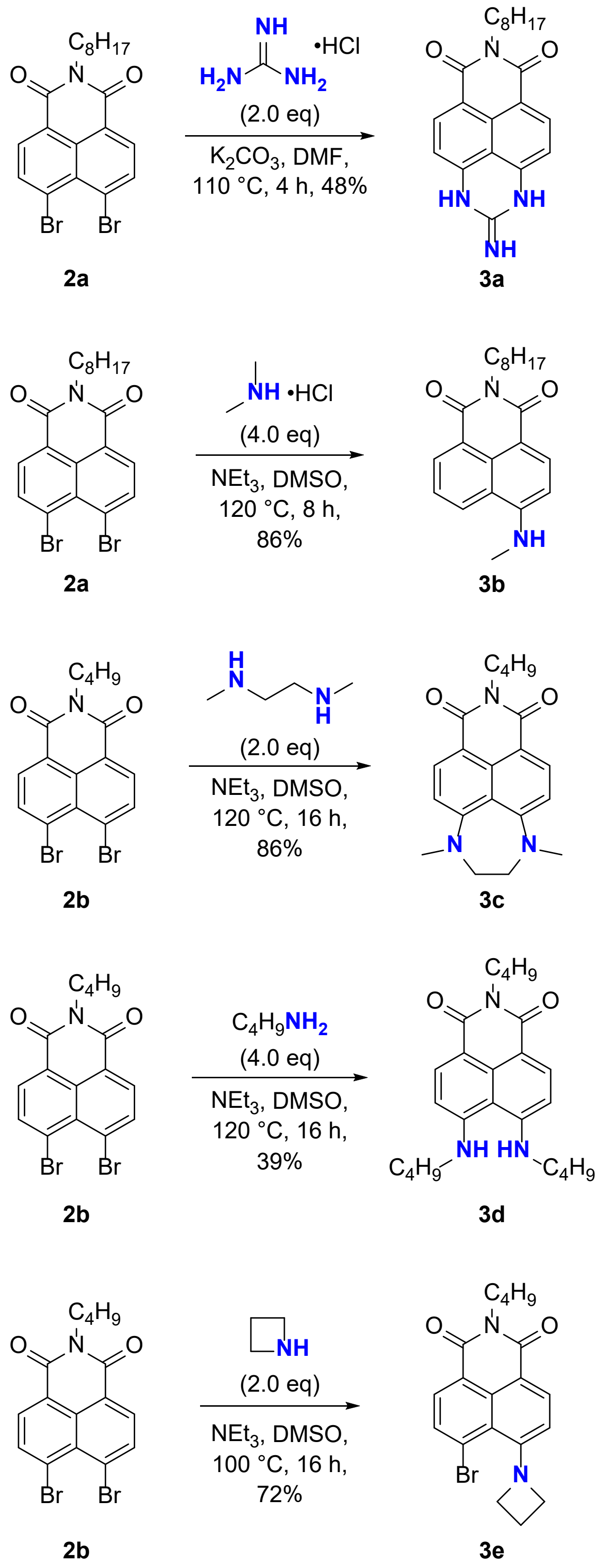

Scheme S2. Preparation of 1,8-naphthalimides **3a-e**.

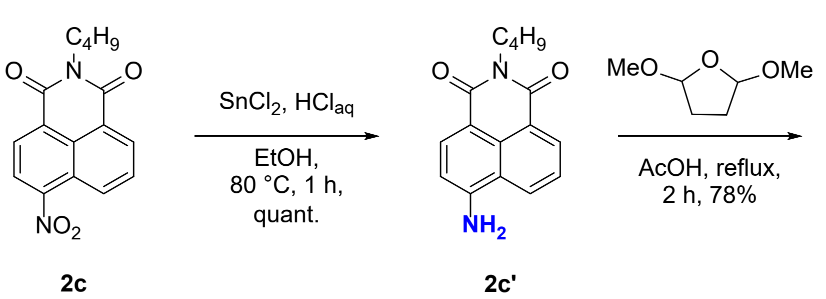

Scheme S3. The synthesis of 1,8-naphthalimide **3f**.

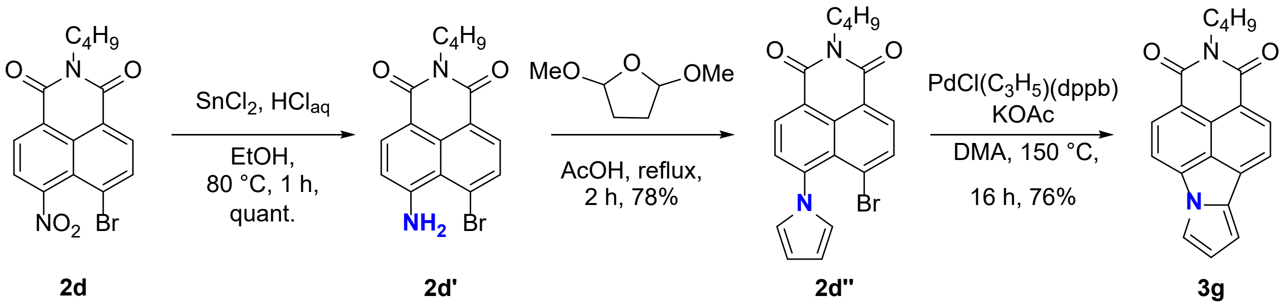

Scheme S4. The synthesis of 1,8-naphthalimide **3g**.

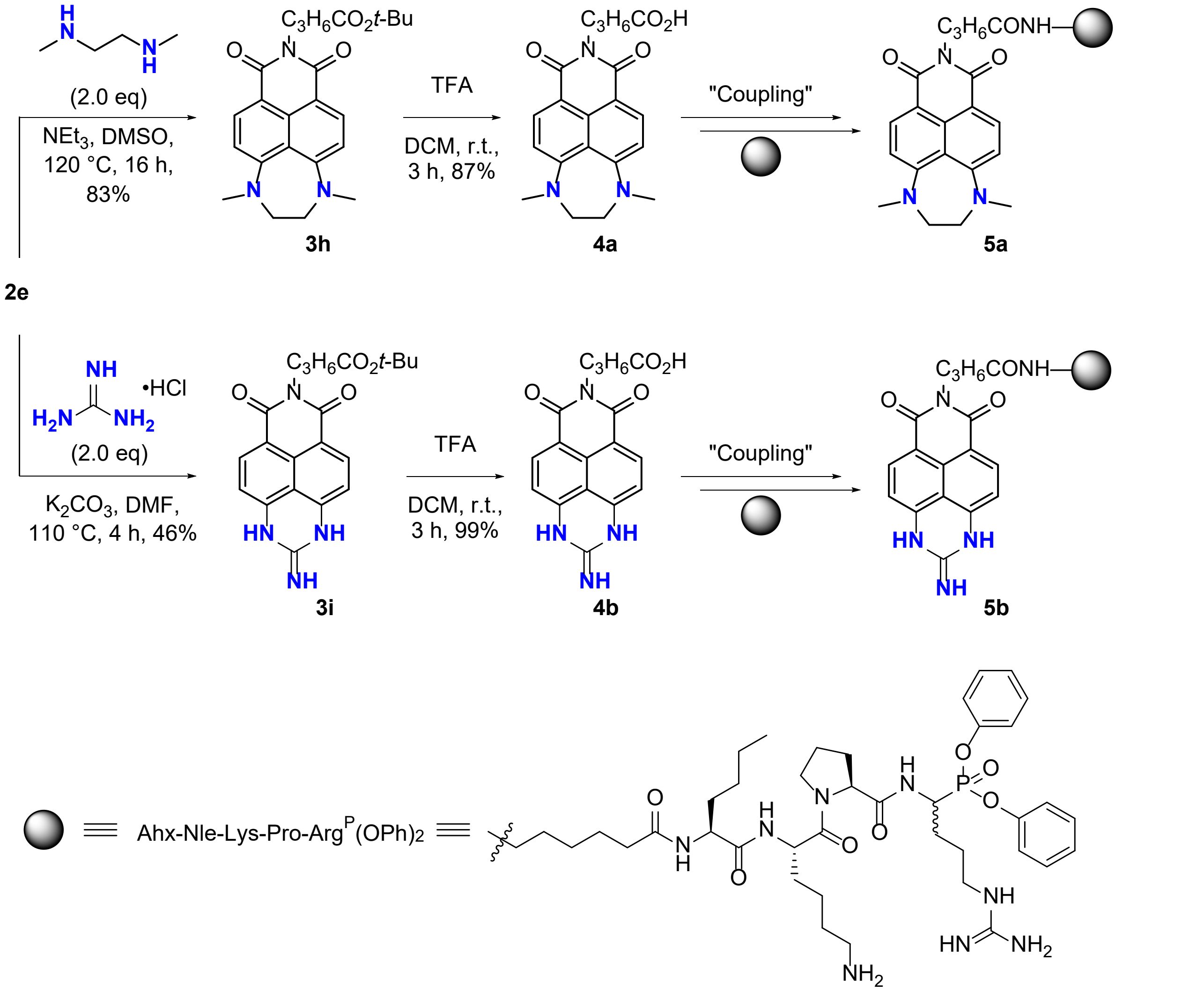

Scheme S5. Preparation of activity-based probes **5a-b** (see the *Biological Studies* paragraph, for details of the coupling reactions performed).

General Synthetic Procedure for 1,8-naphthalimides 2a-e

Parent naphthalic anhydrides **1a-c** (1.0 equiv) were suspended in ethanol or ethanol/toluene (1/1 v/v) mixture. To the suspensions were added the corresponding amines (1.0–2.0 equiv), and the resulting mixtures were then stirred in an oil bath at reflux for 3–5 h under argon atmosphere. The solvents were evaporated, and the crude products were purified by a silica-gel column chromatography.

General Synthetic Procedure for 1,8-naphthalimides 3a/3i

1,8-naphthalimides **2a**/**2e** (1.0 equiv), guanidine hydrochloride (2.0 equiv) and K_2CO_3 (4.0 equiv) were suspended in 10 ml DMF. The resulting mixture was stirred and heated in an oil bath at 110 °C for 4 h under argon atmosphere. The solvent was evaporated and the crude product was purified by a silica-gel column chromatography.

General Synthetic Procedure for 1,8-naphthalimides 3b-e

1,8-naphthalimides **2a-b** (1.0 equiv), corresponding amine (or amine hydrochloride) (2.0 equiv) and triethylamine (0.2 mL) were dissolved in anhydrous DMSO (10 mL). The resulting mixture was stirred in an oil bath at 120 °C for 16 h under argon atmosphere. The solvent was evaporated and the crude product was purified by a silica-gel column chromatography.

N-Octyl-4,5-dibro-1,8-naphthalimide (**2a**):

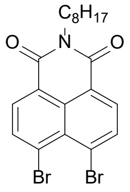

2a

4,5-Dibromo-1,8-naphthalic anhydride (**1a**) (1.07 g, 3.0 mmol) was suspended in a mixture of ethanol and toluene (25/25 mL/mL). To the suspension *n*-octylamine (0.78 g, 1.0 mL, 6.0 mmol) was added and the resulting mixture was stirred at reflux for 3 hours under argon atmosphere. Solvents were evaporated, the crude product was purified by a silica-gel column chromatography (hexanes/CH_2Cl_2, 2:1). Collected product was then washed with a cold hexane, affording **2a** (0.73 g, 52%) as a pale yellow solid. M.p. = 106 – 107 °C. **^{1}H NMR (500 MHz, $CDCl_3$)** *δ* 8.38 (d, *J* = 8.0 Hz, 2H), 8.19 (d, *J* = 8.0 Hz, 2H), 4.13 (t, *J* = 6.9 Hz, 2H), 1.71 (quint, *J* = 7.4 Hz, 2H), 1.44 – 1.22 (m, 10H), 0.87 (t, *J* = 7.1 Hz, 3H). **^{13}C NMR (126 MHz, $CDCl_3$)** *δ* 163.3, 136.3, 131.6, 131.3, 128.2, 127.9, 123.3, 40.9, 31.9, 29.43, 29.34, 28.1, 27.2, 22.8, 14.2. **HRMS (ESI)** m/z: $[M+H]^+$ Calcd for $C_{20}H_{22}Br_2O_2N$ 466.0017; Found 466.0012.

N-Butyl-4,5-dibro-1,8-naphthalimide (**2b**):

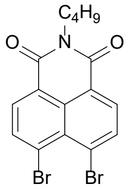

2b

4,5-Dibromo-1,8-naphthalic anhydride (**1a**) (1.07 g, 3.0 mmol) was suspended in a mixture of ethanol and toluene (25/25 mL/mL). To the suspension *n*-butylamine (0.33 g, 0.45 mL, 4.5 mmol) was added and the resulting mixture was stirred at reflux for 5 hours under argon atmosphere. The solvents were then removed, and the crude product was purified by a silica-gel column chromatography (CH_2Cl_2/AcOEt, 98:2). Collected product was then washed with a cold hexane, affording **2a** (0.70 g, 57%) as a pale yellow solid. M.p. = 187 – 189 °C. **^{1}H NMR (500 MHz, $CDCl_3$)** *δ* 8.39 (d, *J* = 8.0 Hz, 2H), 8.20 (d, *J* = 8.0 Hz, 2H), 4.17 – 4.13 (m, 2H), 1.74 – 1.67 (m, 2H), 1.44 (sex, *J* = 7.5 Hz, 2H), 0.98 (t, *J* = 7.4 Hz, 3H). **^{13}C NMR (126 MHz, $CDCl_3$)** *δ* 163.3, 136.3, 131.6, 131.4, 128.2, 127.9, 123.3, 40.7, 30.2, 20.5, 13.9. **HRMS (APCI)** m/z: $[M+H]^+$ Calcd for $C_{16}H_{14}Br_2O_2N$ 409.9391; Found 409.9382.

N-Butyl-4-nitro-1,8-naphthalimide (**2c**):

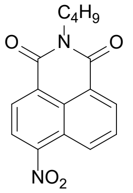

2c

4-Nitro-1,8-naphthalic anhydride (**1b**) (0.97 g, 4.0 mmol) was suspended in ethanol (20 mL). To the suspension *n*-butylamine (0.32 g, 0.44 mL, 4.4 mmol) was added and the resulting mixture was stirred at reflux for 3 hours. The solvent was removed, and the crude product was purified by a silica-gel column chromatography (CH_2Cl_2), affording **2c** (0.83 g, 69%) as a pale yellow solid. M.p. = 90 – 91 °C. **^{1}H NMR (500 MHz, $CDCl_3$)** *δ* 8.84 (dd, *J* = 8.7, 0.7 Hz, 1H), 8.74 (dd, *J* = 7.2, 0.6 Hz, 1H), 8.69 (d, *J* = 8.0 Hz, 1H), 8.40 (d, *J* = 8.0 Hz, 1H), 7.98 (dd, *J* = 8.6, 7.4 Hz, 1H), 4.22 – 4.17 (m, 2H), 1.77 – 1.69 (m, 2H), 1.45 (sex, *J* = 7.5 Hz, 2H), 0.99 (t, *J* = 7.4 Hz, 3H). Spectral properties were in agreement with those previously reported.[5]

N-Butyl-4-amino-1,8-naphthalimide (**2c'**):

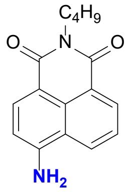

2c'

N-butyl-4-nitro-1,8-naphthalimide (**2c**) (0.81 g, 2.7 mmol) and tin(II) chloride dihydrate (3.06 g, 13.5 mmol) were suspended in ethanol (15 mL) and conc. hydrochloric acid (35%, 7 mL) was added dropwise. The resulting suspension was stirred at 80 °C for 1 hour. Then, the mixture was filtered and washed with water and ethanol, affording **2c'** (0.72 g, quant.) as an orange solid. The compound was used without further purification. M.p. = 182 – 183 °C. **^{1}H NMR (500 MHz, $CDCl_3$)** 8.59 (dd, *J* = 7.3, 0.5 Hz, 1H), 8.41 (d, *J* = 8.1 Hz, 1H), 8.10 (d, *J* = 8.3 Hz, 1H), 7.64 (dd, *J* = 8.2, 8.1 Hz, 1H), 6.88 (d, *J* = 8.1 Hz, 1H), 4.97 (bs, 2H), 4.19 – 4.13 (m, 2H), 1.75 – 1.67 (m, 2H), 1.44 (sex, *J* = 7.5 Hz, 2H), 0.96 (t, *J* = 7.4 Hz, 3H). **^{13}C NMR (126 MHz, $CDCl_3$)** *δ* 164.7, 164.2, 149.1, 133.8, 131.6, 129.9, 126.9, 125.1, 123.4, 120.3, 112.5, 109.7, 40.2, 30.4, 20.6, 14.0. **HRMS (ESI)** m/z: $[M+H]^+$ Calcd for $C_{16}H_{17}O_2N_2$ 269.1290; Found 269.1296.

N-Butyl-4-bromo-5-nitro-1,8-naphthalimide (**2d**):

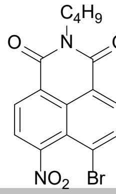

2d

4-Bromo-5-nitro-1,8-naphthalic anhydride (**1c**) (1.29 g, 4.0 mmol) was suspended in ethanol (20 mL). To the suspension *n*-butylamine (0.30 g, 0.40 mL, 4.0 mmol) was added and the resulting mixture was stirred at 70 °C for 3 hours under argon atmosphere. The solvent was then removed, and the crude product was purified by a silica-gel column chromatography (CH_2Cl_2), affording **2d** (0.50 g, 33%) as an orange solid. M.p. = 166 – 168 °C. **^{1}H NMR (500 MHz, $CDCl_3$)** *δ* 8.70 (d, *J* = 7.8 Hz, 1H), 8.51 (d, *J* = 7.9 Hz, 1H), 8.21 (d, *J* = 7.9 Hz, 1H), 7.92 (d, *J* = 7.8 Hz, 1H), 4.19 – 4.14 (m, 2H), 1.75 – 1.67 (m, 2H), 1.44 (sex, *J* = 7.5 Hz, 2H), 0.98 (t, *J* = 7.4 Hz, 3H). **^{13}C NMR (126 MHz, $CDCl_3$)** *δ* 162.9, 162.1, 151.4, 136.1, 132.4, 131.3, 130.7, 125.9, 124.2, 123.7, 122.6, 121.4, 40.9, 30.2, 20.5, 13.9. **HRMS (APCI)** m/z: $[M+H]^+$ Calcd for $C_{16}H_{14}BrO_4N_2$ 377.0137; Found: 377.0131.

N-Butyl-4-amino-5-bromo-1,8-naphthalimide (**2d'**):

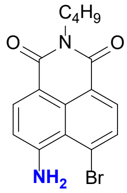

2d'

N-butyl-4-bromo-5-nitro-1,8-naphthalimide (**2d**) (0.42 g, 1.1 mmol) and tin(II) chloride dihydrate (1.25 g, 5.5 mmol) were suspended in ethanol (6 mL) and conc. hydrochloric acid (35%, 3 mL) was added dropwise. The resulting suspension was stirred at 80 °C for 1 hour. Then, the mixture was filtered and washed with water and ethanol, affording **2d'** (0.38 g, 99 %) as a yellow solid. M.p. = 209 – 211 °C. The compound was used without further purification. **^{1}H NMR (500 MHz, $CDCl_3$)** *δ* 8.41 (d, *J* = 8.3 Hz, 1H), 8.33 (d, *J* = 7.9 Hz, 1H), 7.83 (d, *J* = 7.9 Hz, 1H), 6.84 (d, *J* = 8.4 Hz, 1H), 6.13 (bs, 2H), 4.16 – 4.10 (m, 2H), 1.73 – 1.66 (m, 2H), 1.43 (sex, *J* = 7.5 Hz, 2H), 0.96 (t, *J* = 7.4 Hz, 3H). **^{13}C NMR (126 MHz, $CDCl_3$)** *δ* 164.1, 163.7, 150.6, 134.4, 132.6, 131.9, 131.7, 125.6, 122.9, 117.7, 112.3, 112.1, 40.3, 30.3, 20.5, 14.0. **HRMS (APCI)** m/z: $[M+H]^+$ Calcd for $C_{16}H_{16}BrO_2N_2$ 347.0395; Found: 347.0400.

6-Bromo-2-butyl-7-(1*H*-pyrrol-1-yl)-1*H*-benzo[*de*]isoquinoline-1,3(2*H*)-dione (**2d''**):

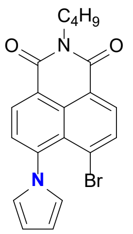

2d''

N-butyl-4-amino-5-bromo-1,8-naphthalimide (**2d'**) (0.35 g, 1.0 mmol) and dimethoxytetrahydrofuran (0.15 g, 0.14 mL, 1.1 mmol) were dissolved in glacial acetic acid (4 mL). The resulting mixture was stirred under reflux for 2 hours. Then, the solvent was removed, and the crude product was purified by a silica-gel column chromatography (CH_2Cl_2), affording **2d''** (0.31 g, 78%) as a yellow solid. M.p. = 113 – 115 °C. **^{1}H NMR (500 MHz, $CDCl_3$)** δ 8.68 (d, *J* = 7.7 Hz, 1H), 8.44 (d, *J* = 7.9 Hz, 1H), 8.10 (d, *J* = 7.9 Hz, 1H), 7.75 (d, *J* = 7.7 Hz, 1H), 6.81 – 6.78 (m, 2H), 6.42 – 6.39 (m, 2H), 4.22 – 4.16 (m, 2H), 1.77 – 1.69 (m, 2H), 1.45 (sex, *J* = 7.5 Hz, 2H), 0.99 (t, *J* = 7.4 Hz, 3H). **^{13}C NMR (126 MHz, $CDCl_3$)** δ 163.7, 163.2, 143.3, 135.4, 131.62, 131.57, 131.0, 128.9, 126.4, 125.7, 124.4, 123.2, 122.7, 110.5, 40.6, 30.3, 20.5, 14.0. **HRMS (ESI)** m/z: $[M+H]^+$ Calcd for $C_{20}H_{18}BrO_2N_2$ 397.0552; Found 397.0544.

Tert-butyl 4-(6,7-dibromo-1,3-dioxo-1*H*-benzo[de]isoquinolin-2(3*H*)-yl)butanoate (**2e**):

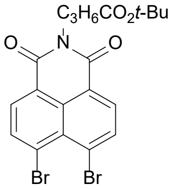

2e

4,5-Dibromo-1,8-naphthalic anhydride (**1a**) (1.07 g, 3.0 mmol) was suspended in a mixture of ethanol and toluene (25/25 mL/mL). To the suspension *tert*-butyl 4-aminobutyrate (0.72 g, 4.5 mmol) was added and the resulting mixture was stirred at reflux for 4 hours under argon atmosphere. The solvents were then removed, and the crude product was purified by a silica-gel column chromatography (CH_2Cl_2/AcOEt, 98:2). Collected product was then washed with a cold hexane, affording **2e** (0.52 g, 35%) as a pale yellow solid. M.p. = 169 – 171 °C. **^{1}H NMR (500 MHz, $CDCl_3$)** δ 8.40 (d, *J* = 8.0 Hz, 2H), 8.21 (d, *J* = 8.0 Hz, 2H), 4.22 – 4.18 (m, 2H), 2.34 (t, *J* = 7.5 Hz, 2H), 2.03 (quint, *J* = 7.3 Hz, 2H), 1.42 (s, 9H). **^{13}C NMR (126 MHz, $CDCl_3$)** δ 172.2, 163.3, 136.3, 131.7, 131.4, 128.4, 128.0, 123.2, 80.5, 40.1, 33.3, 28.2, 23.5. **HRMS (ESI)** m/z: $[M+Na]^+$ Calcd for $C_{20}H_{19}Br_2O_4NNa$ 517.9579; Found 517.9572.

2-Imino-7-octyl-2,3-dihydropyrido[3,4,5-*gh*]perimidine-6,8(1*H*,7*H*)-dione (**3a**):

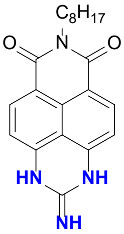

3a

N-octyl-4,5-dibro-1,8-naphthalimide (**2a**) (117 mg, 0.25 mmol), guanidine hydrochloride (48 mg, 0.5 mmol) and K_2CO_3 (138 mg, 1.0 mmol) were suspended in DMF (10 ml). The resulting mixture was stirred and heated at 110 °C for 4 hours under argon atmosphere. The solvent was evaporated and the crude product was purified by a silica-gel column chromatography (CH_2Cl_2/MeOH, 9:1), affording **3e** (43 mg, 48%) as a yellow solid. M.p. = >400 °C. **^{1}H NMR (500 MHz, [D_6]-DMSO)** δ 11.89 (bs, 1H), 8.19 (d, *J* = 8.3 Hz, 2H), 7.04 (bs, 2H), 6.79 (d, *J* = 8.3 Hz, 2H), 4.02 – 3.96 (m, 2H), 1.60 – 1.52 (m, 2H), 1.33 – 1.18 (m, 10H), 0.84 (t, *J* = 6.9 Hz, 3H). **^{13}C NMR (126 MHz, [D_6]-DMSO)** δ 162.9, 153.3, 134.2, 130.1, 113.9, 31.2, 28.8, 28.6, 27.6, 26.6, 22.1, 13.9. **HRMS (ESI)** m/z: $[M+H]^+$ Calcd for $C_{21}H_{25}O_2N_4$ 365.1978; Found: 365.1986.

6-(Methylamino)-2-octyl-1*H*-benzo[*de*]isoquinoline-1,3(2*H*)-dione (**3b**):

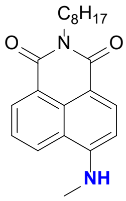

3b

N-octyl-4,5-dibro-1,8-naphthalimide (**2a**) (120 mg, 0.26 mmol), dimethylamine hydrochloride (89 mg, 1.10 mmol), trimethylamine (290 mg, 0.4 mL, 2.86 mmol) were dissolved in anhydrous DMSO (10 mL). The resulting mixture was stirred at 120 °C for 16 hours under argon atmosphere. The solvent was evaporated and the crude product was purified by a silica-gel column chromatography (CH_2Cl_2/MeOH, 98:2), affording **3b** (87 mg, 86%) as a bright orange solid. M.p. = 153 – 155 °C. **^{1}H NMR (500 MHz, $CDCl_3$)** δ 8.57 (dd, *J* = 7.6, 0.7 Hz, 1H), 8.48 (d, *J* = 8.4 Hz, 1H), 8.08 (d, *J* = 8.3 Hz, 1H), 7.61 (dd, *J* = 8.1, 7.9 Hz, 1H), 6.74 (d, *J* = 8.2 Hz, 1H), 4.17 – 4.12 (m, 2H), 3.14 (s, 3H), 1.76 – 1.68 (m,

2H), 1.45 – 1.20 (m, 10H), 0.86 (t, *J* = 6.9 Hz, 3H). **^{13}C NMR (126 MHz, $CDCl_3$)** *δ* 164.8, 164.3, 134.4, 131.2, 129.8, 125.9, 125.03, 125.00, 123.5, 40.4, 32.0, 29.8, 29.5, 29.4, 28.4, 27.4, 22.8, 14.2. **HRMS (ESI)** m/z: $[M+H]^+$ Calcd for $C_{21}H_{27}O_2N_2$ 339.2073; Found: 339.2079.

8-Butyl-1,4-dimethyl-1,2,3,4-tetrahydro-7*H*-pyrido[3',4',5':4,5]naphtho[1,8-*ef*][1,4]diazepine-7,9(8*H*)-dione (**3c**):

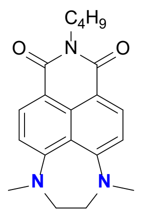

3c

N-butyl-4,5-dibro-1,8-naphthalimide (**2b**) (103 mg, 0.25 mmol), 1,2-dimethylethylenediamine (DMEDA) (44 mg, 54 μL, 0.50 mmol), trimethylamine (101 mg, 0.14 mL, 1.0 mmol) were dissolved in anhydrous DMSO (10 mL). The resulting mixture was stirred at 120 °C for 16 hours under argon atmosphere. The solvent was evaporated and the crude product was purified by a silica-gel column chromatography (CH_2Cl_2/AcOEt, 98:2), affording **3c** (77 mg, 86%) as a bright orange solid. M.p. = 119 – 121 °C. **^{1}H NMR (500 MHz, $CDCl_3$)** *δ* 8.43 (d, *J* = 8.4 Hz, 2H), 6.80 (d, *J* = 8.5 Hz, 2H), 4.17 – 4.12 (m, 2H), 3.58 (s, 4H), 3.15 (s, 6H), 1.74 – 1.66 (m, 2H), 1.48 – 1.39 (m, 2H), 0.96 (t, *J* = 7.4 Hz, 3H). **^{13}C NMR (126 MHz, $CDCl_3$)** *δ* 164.6, 154.9, 132.9, 132.8, 117.7, 113.0, 110.4, 57.9, 41.8, 40.0, 30.5, 20.6, 14.1. **HRMS (ESI)** m/z: $[M+H]^+$ Calcd for $C_{20}H_{24}O_2N_3$ 338.1869; Found: 338.1878.

2-Butyl-6,7-bis(butylamino)-1*H*-benzo[*de*]isoquinoline-1,3(2*H*)-dione (**3d**):

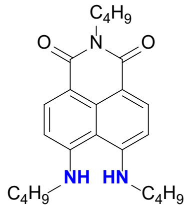

3d

N-butyl-4,5-dibro-1,8-naphthalimide (**2b**) (103 mg, 0.25 mmol), *n*-butylamine (74 mg, 0.1 mL, 1.0 mmol), trimethylamine (101 mg, 0.14 mL, 1.0 mmol) were dissolved in anhydrous DMSO (10 mL). The resulting mixture was stirred at 120 °C for 16 h under argon atmosphere. The solvent was evaporated and the crude product was purified by a silica-gel column chromatography (CH_2Cl_2/AcOEt, 98:2), affording **3d** (38 mg, 39%) as a yellow solid. M.p. = 186 – 187 °C. **^{1}H NMR (500 MHz, $CDCl_3$)** *δ* 8.39 (d, *J* = 8.4 Hz, 2H), 6.73 (d, *J* = 8.4 Hz, 2H), 5.66 (bs, 2H), 4.16 – 4.11 (m, 2H), 3.23 (t, *J* = 7.0 Hz, 4H), 1.79 – 1.71 (m, 4H), 1.71 – 1.65 (m, 2H), 1.53 (sex, *J* = 7.5 Hz 4H), 1.43 (sex, *J* = 7.5 Hz, 2H), 1.03 (t, *J* = 7.4 Hz, 6H), 0.96 (t, *J* = 7.4 Hz, 3H). **^{13}C NMR (126 MHz, $CDCl_3$)** *δ* 164.7, 152.6, 133.6, 132.2, 112.5, 111.8, 107.3, 44.7, 39.9, 31.4, 30.5, 20.68, 20.62, 14.1, 14.0. **HRMS (ESI)** m/z: $[M+H]^+$ Calcd for $C_{24}H_{34}O_2N_3$ 396.2651; Found: 396.2658.

6-(Azetidin-1-yl)-7-bromo-2-butyl-1*H*-benzo[*de*]isoquinoline-1,3(2*H*)-dione (**3e**):

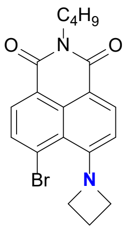

3e

N-butyl-4,5-dibro-1,8-naphthalimide (**2b**) (103 mg, 0.25 mmol), azetidine (29 mg, 35 μL, 0.5 mmol), trimethylamine (101 mg, 0.14 mL, 1.0 mmol) were dissolved in anhydrous DMSO (10 mL). The resulting mixture was stirred at 100 °C for 16 h under argon atmosphere. The solvent was evaporated and the crude product was purified by a silica-gel column chromatography (CH_2Cl_2/AcOEt, 99:1). Obtained orange oil was triturated with cold hexane, and formed crystals were filtered off, affording **3e** (66 mg, 72%) as an orange solid. M.p. = 108 – 110 °C. **^{1}H NMR (500 MHz, $CDCl_3$)** *δ* 8.46 (d, *J* = 8.5 Hz, 1H), 8.30 (d, *J* = 7.9 Hz, 1H), 7.81 (d, *J* = 7.8 Hz, 1H), 6.77 (d, *J* = 8.5 Hz, 1H), 4.24 (t, *J* = 7.5 Hz, 4H), 4.16 – 4.12 (m, 2H), 2.43 (quint, *J* = 7.4 Hz, 2H), 1.73 – 1.65 (m, 2H), 1.43 (sex, *J* = 7.5 Hz, 2H), 0.96 (t, *J* = 7.4 Hz, 3H). **^{13}C NMR (126 MHz, $CDCl_3$)** *δ* 164.3, 163.8, 156.2, 133.0, 132.4, 131.5, 131.2, 125.9, 121.9, 120.8, 112.3, 110.1, 57.0, 40.2, 30.4, 20.5, 17.4, 14.0. **HRMS (ESI)** m/z: $[M+H]^+$ Calcd for $C_{19}H_{20}BrO_2N_2$ 387.0708; Found 387.0711.

2-Butyl-6-(1*H*-pyrrol-1-yl)-1*H*-benzo[*de*]isoquinoline-1,3(2*H*)-dione (**3f**):

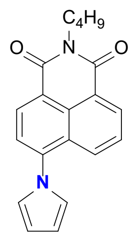

3f

N-butyl-4-amino-1,8-naphthalimide (**2c'**) (0.67 g, 2.5 mmol) and dimethoxytetrahydrofuran (0.36 g, 0.36 mL, 2.75 mmol) were dissolved in glacial acetic acid (10 mL). The resulting mixture was stirred under reflux for 2 hours. Then, the solvent was removed, and the crude product was purified by a silica-gel column chromatography (CH_2Cl_2/hexanes 4:1). Obtained product was triturated with cold hexane, and formed crystals were filtered off, affording **3f** (0.62 g, 78%) as a yellow solid. M.p. = 137 – 138 °C. **^{1}H NMR (500 MHz, $CDCl_3$)** *δ* 8.65 (dd, J = 7.4, 0.8 Hz, 1H), 8.64 (d, *J* = 7.8 Hz, 1H), 8.29 (dd, *J* = 8.5, 0.8 Hz, 1H), 7.77 (dd, *J* = 8.4, 7.4 Hz, 1H), 7.68 (d, *J* = 7.8 Hz, 1H), 7.08 – 7.05 (m, 2H), 6.50 – 6.48 (m, 2H), 4.23 – 4.18 (m, 2H), 1.77 – 1.70 (m, 2H), 1.46 (sex, *J* = 7.6 Hz, 2H), 0.99 (t, *J* = 7.4 Hz, 3H). **^{13}C NMR (126 MHz, $CDCl_3$)** *δ* 164.2, 163.7, 143.5, 131.9, 131.4, 130.2, 129.4, 127.7, 127.5, 123.5, 123.25, 123.22, 121.6, 110.9, 40.5, 30.3, 20.5, 14.0. **HRMS (ESI)** m/z: $[M+H]^+$ Calcd for $C_{20}H_{19}O_2N_2$ 319.1447; Found 319.1454.

2-Butyl-1*H*-pyrrolo[1',2':1,2]indolo[5,4,3-*def*]isoquinoline-1,3(2*H*)-dione (**3g**):

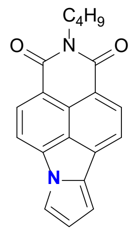

3g

The reaction flask was charged with 6-Bromo-2-butyl-7-(1*H*-pyrrol-1-yl)-1*H*-benzo[*de*]isoquinoline- 1,3(2*H*)-dione (**2d''**) (100 mg, 0.25 mmol), $PdCl(C_3H_5)$(dppb) (8 mg, 0.0125 mmol, 5 mol%) and potassium acetate (50 mg, 0.5 mmol) while purging the flask with argon. The reaction set-up was evacuated and backfilled with argon three times and dry DMA (6 mL) was added to flask with the constant argon flow. The resulting mixture was stirred at 150 °C for 16 hours. Then, solvent was evaporated. After evaporation of the reaction mixture, the residue was dissolved in dichloromethane (25 ml) and transferred to the separatory funnel. Organic layer was washed with distilled water (2 x 25 mL), brine (25 mL) and dried over anhydrous $MgSO_4$. Then, the drying agent was filtered off, solvent was evaporated, and the crude product was purified by a silica-gel column chromatography (CH_2Cl_2). Obtained product was pseuodorecrystillized with cyclohexane, and formed crystals were filtered off, affording **3g** (60 mg, 76%) as a bright red solid. M.p. = 186 – 188 °C. **^{1}H NMR (500 MHz, $CDCl_3$)** *δ* 8.26 (d, *J* = 7.4 Hz, 1H), 8.24 (d, *J* = 7.7 Hz, 1H), 7.45 (d, *J* = 7.4 Hz, 1H), 7.18 (d, *J* = 7.6 Hz, 1H), 7.14 (d, *J* = 2.8 Hz, 1H), 6.54 (d, *J* = 3.5 Hz, 1H), 6.28 – 6.24 (m, 1H), 4.14 – 4.08 (m, 2H), 1.72 – 1.65 (m, 2H), 1.44 (sex, *J* = 7.5 Hz, 2H), 0.98 (t, *J* = 7.4 Hz, 3H). **^{13}C NMR (126 MHz, $CDCl_3$)** *δ* 163.8, 163.6, 141.1, 137.2, 134.1, 133.1, 132.9, 128.6, 126.1, 119.7, 118.46, 118.42, 118.2, 116.1, 110.3, 107.5, 40.2, 30.7, 20.5, 14.0. **HRMS (ESI)** m/z: $[M+H]^+$ Calcd for $C_{20}H_{17}O_2N_2$ 317.1290; Found 317.1294.

Tert-butyl 4-(1,4-dimethyl-7,9-dioxo-1,2,3,4,7,9-hexahydro-8*H* pyrido[3',4',5':4,5]naphtho[1,8-*ef*][1,4]diazepin-8-yl)butanoate (**3h**):

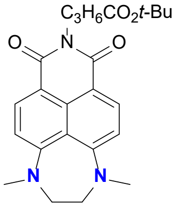

3h

Tert-butyl 4-(6,7-dibromo-1,3-dioxo-1*H*-benzo[de]isoquinolin-2(3*H*)-yl)butanoate (**2e**) (124 mg, 0.25 mmol), 1,2-dimethylethylenediamine (DMEDA) (44 mg, 54 μL, 0.50 mmol), trimethylamine (101 mg, 140 μL, 1.0 mmol) were dissolved in anhydrous DMSO (10 mL). The resulting mixture was stirred at 120 °C for 16 h under argon atmosphere. The solvent was evaporated and the crude product was purified by a silica-gel column chromatography (CH_2Cl_2/AcOEt, 98:2), affording **3h** (88 mg, 83%) as a bright orange solid. M.p. = 122 – 124 °C. **^{1}H NMR (500 MHz, $CDCl_3$)** *δ* 8.42 (d, *J* = 8.4 Hz, 2H), 6.78 (d, *J* = 8.5 Hz, 2H), 4.19 (t, *J* = 7.2 Hz, 2H), 3.58 (s, 4H), 3.15 (s, 6H), 2.33 (t, *J* = 7.8 Hz, 2H), (quint, *J* = 7.4 Hz 2H), 1.42 (s, 9H). **^{13}C NMR (126 MHz, $CDCl_3$)** *δ* 172.6, 164.5, 155.1, 133.0, 132.9, 117.7, 112.8, 110.3, 80.1, 57.9, 41.7, 39.3, 33.6, 28.2, 23.9. **HRMS (ESI)** m/z: $[M+Na]^+$ Calcd for $C_{24}H_{29}O_4N_3Na$ 446.2056; Found 446.2062.

Tert-butyl 4-(2-imino-6,8-dioxo-1,3,6,8-tetrahydropyrido[3,4,5-*gh*]perimidin-7(2*H*)-yl)butanoate (**3i**):

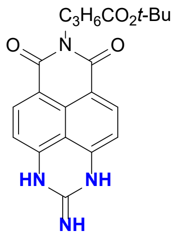

3i

Tert-butyl 4-(6,7-dibromo-1,3-dioxo-1*H*-benzo[de]isoquinolin-2(3*H*)-yl)butanoate (**2e**) (124 mg, 0.25 mmol), guanidine hydrochloride (48 mg, 0.5 mmol) and K_2CO_3 (138 mg, 1.0 mmol) were suspended in DMF (10 ml). The resulting mixture was stirred and heated at 110 °C for 4 h under argon atmosphere. The solvent was evaporated and the crude product was purified by a silica-gel column chromatography (CH_2Cl_2/MeOH, 9:1), affording **3e** (45 mg, 46%) as a yellow solid. M.p. = >400 °C. **^{1}H NMR (500 MHz, [D$_6$]-DMSO)** δ 11.55 (bs, 1H), 8.20 (d, J = 8.3 Hz, 2H), 6.99 (bs, 2H), 6.80 (d, J = 8.2 Hz, 2H), 4.03 (t, J = 7.0 Hz, 2H), 2.23 (t, J = 7.4 Hz, 2H), 1.82 (quint, J = 7.1 Hz, 2H), 1.35 (s, 9H). **^{13}C NMR (126 MHz, [D$_6$]-DMSO)** δ 171.7, 163.0, 153.2, 134.3, 134.2, 130.1, 113.9, 79.5, 64.9, 38.5, 32.6, 27.7, 23.3, 21.7, 15.1, 13.9. **HRMS (ESI)** m/z: $[M+H]^+$ Calcd for $C_{21}H_{23}O_4N_4$ 395.1719; Found 395.1723.

4-(1,4-Dimethyl-7,9-dioxo-1,2,3,4,7,9-hexahydro-8*H*-pyrido[3',4',5':4,5]naphtho[1,8-*ef*][1,4]diazepin-8-yl)butanoic acid (**4a**):

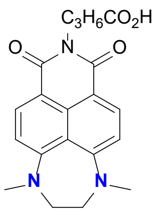

4a

3h (42 mg, 0.1 mmol) was dissolved in CH_2Cl_2 (1 mL) and TFA (223 mg, 150 μL, 1.96 mmol) was added dropwise. The resulting mixture was stirred at room temperature for 3 hours. Then, the solvent was evaporated, the product was triturated with hexane and filtered affording **4a** (32 mg, 87%) as an orange solid. The compound was used without further purification. M.p. = 236 – 238 °C. **^{1}H NMR (500 MHz, $CDCl_3$)** δ 8.44 (d, J = 8.5 Hz, 2H), 6.80 (d, J = 8.5 Hz, 2H), 4.26 (t, J = 6.6 Hz, 2H), 3.62 (s, 4H), 3.17 (s, 6H), 2.43 (t, J = 7.2 Hz, 2H), 2.11 (quint, J = 6.8 Hz 2H). (COO**H** proton not visible due to the rapid exchange). **^{13}C NMR (126 MHz, $CDCl_3$)** δ 175.6, 164.9, 155.5, 133.4, 133.0, 117.3, 112.2, 110.5, 58.0, 41.8, 39.1, 32.0, 24.1. **HRMS (ESI)** m/z: $[M+H]^+$ Calcd for $C_{20}H_{22}O_4N_3$ 368.1610; Found 368.1616.

4-(2-Imino-6,8-dioxo-1,3,6,8-tetrahydropyrido[3,4,5-*gh*]perimidin-7(2*H*)-yl)butanoic acid (**4b**):

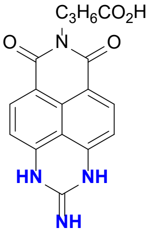

4b

3i (35 mg, 0.09 mmol) was dissolved in CH_2Cl_2 (1 mL) and TFA (300 mg, 200 μL, 2.60 mmol) was added dropwise. The resulting mixture was stirred at room temperature for 3 hours. Then, the solvent was evaporated, the product was triturated with hexane and filtered affording **4b** (30 mg, quant) as a yellow solid. The compound was used without further purification. M.p. = >400 °C. **^{1}H NMR (500 MHz, [D$_6$]-DMSO)** δ 8.27 (d, J = 8.2 Hz, 2H), 7.73 (bs, 2H), 6.95 (d, J = 8.2 Hz, 2H), 4.04 (t, J = 6.9 Hz, 2H), 2.28 – 2.23 (m, 2H), 1.84 (quint, J = 7.0 Hz, 1H). (COO**H** proton not visible due to the rapid exchange). **^{13}C NMR (126 MHz, [D$_6$]-DMSO)** δ 173.9, 162.9, 151.6, 134.2, 129.3, 113.3, 112.0, 108.3, 38.7, 31.4, 23.2. **HRMS (ESI)** m/z: $[M+H]^+$ Calcd for $C_{17}H_{15}O_4N_4$ 339.1093; Found 339.1094.

3. Photophysical properties

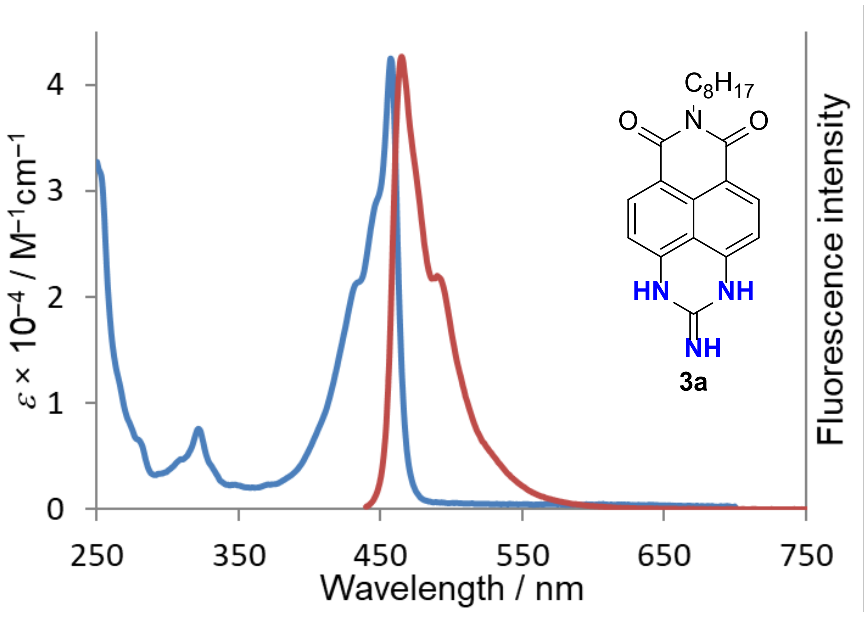

λ_{abs} = 457 nm
ε = 42500 $M^{-1}cm^{-1}$
log ε = 4.63
λ_{em} = 465 nm
Φ_F = 0.681
$\varepsilon \times \Phi_F$ = 28900 $M^{-1}cm^{-1}$

Figure S1. Absorption (blue) and emission (red) spectra of **3a** measured for ethanol.

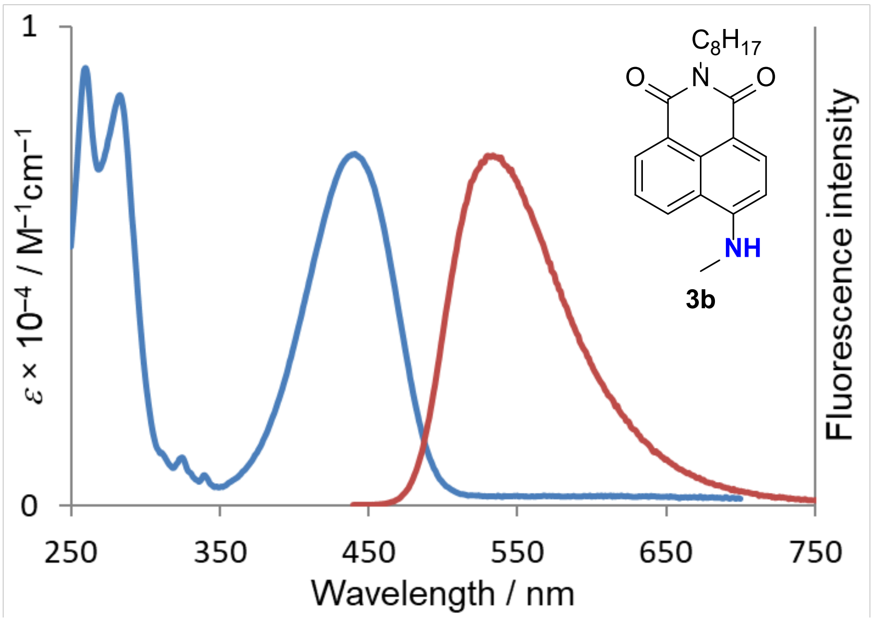

λ_{abs} = 441 nm
ε = 7400 $M^{-1}cm^{-1}$
log ε = 3.87
λ_{em} = 530 nm
Φ_F = 0.564
$\varepsilon \times \Phi_F$ = 4200 $M^{-1}cm^{-1}$

Figure S2. Absorption (blue) and emission (red) spectra of **3b** measured for ethanol.

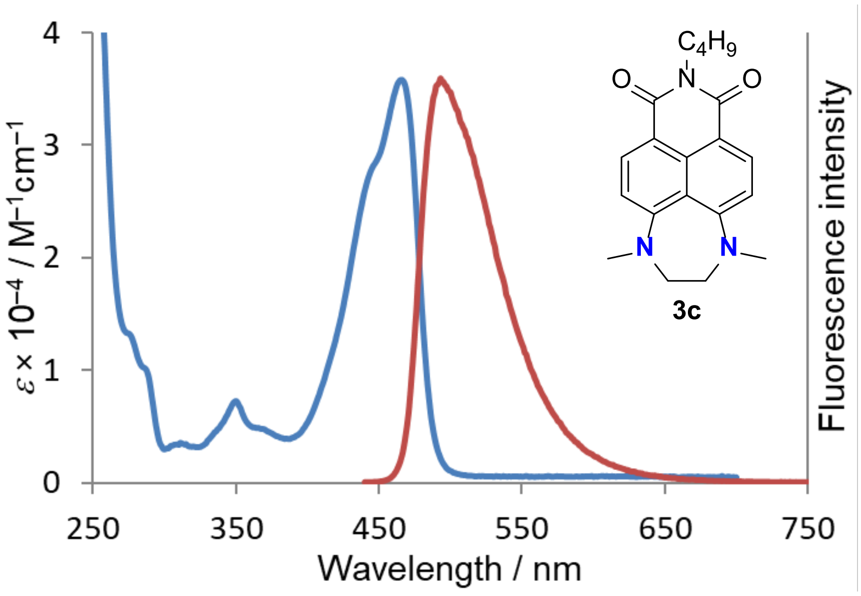

λ_{abs} = 466 nm
ε = 35800 $M^{-1}cm^{-1}$
log ε = 4.55
λ_{em} = 493 nm
Φ_F = 0.760
$\varepsilon \times \Phi_F$ = 27200 $M^{-1}cm^{-1}$

Figure S3. Absorption (blue) and emission (red) spectra of **3c** measured for ethanol.

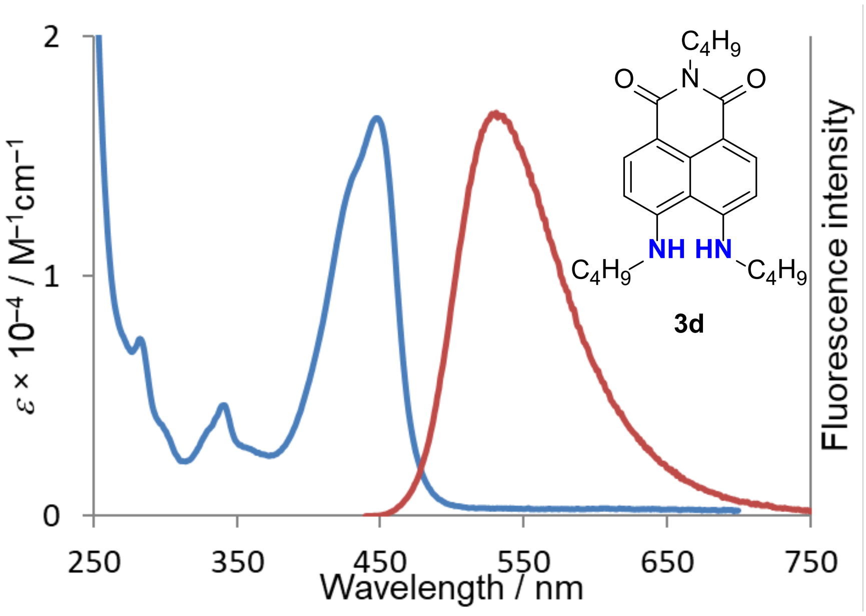

λ_{abs} = 448 nm
ε = 16600 $M^{-1}cm^{-1}$
log ε = 4.22
λ_{em} = 531nm
Φ_F = 0.354
$\varepsilon \times \Phi_F$ = 5900 $M^{-1}cm^{-1}$

Figure S4. Absorption (blue) and emission (red) spectra of **3d** measured for ethanol.

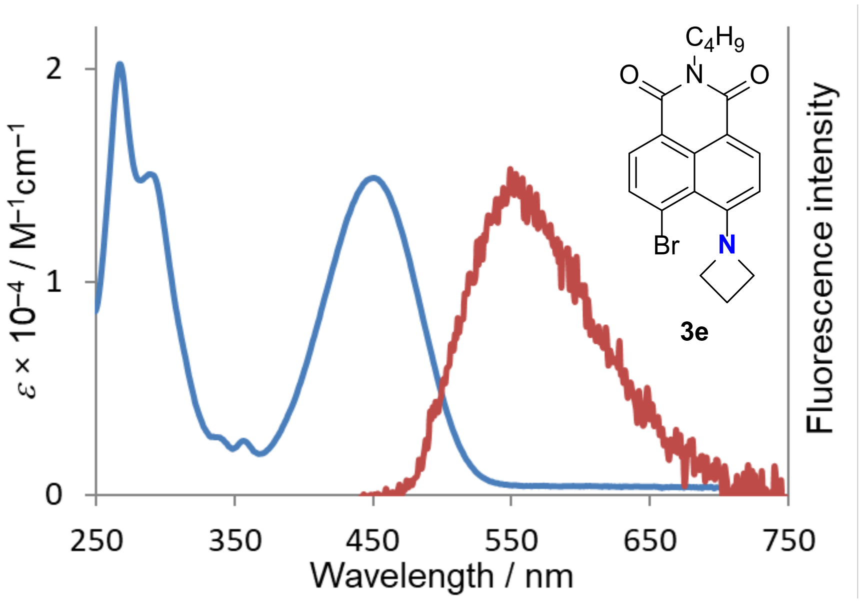

λ_{abs} = 451 nm
ε = 14900 $M^{-1}cm^{-1}$
log ε = 4.17
λ_{em} = 549nm
Φ_F = 0.005
$\varepsilon \times \Phi_F$ = 75 $M^{-1}cm^{-1}$

Figure S5. Absorption (blue) and emission (red) spectra of **3e** measured for ethanol.

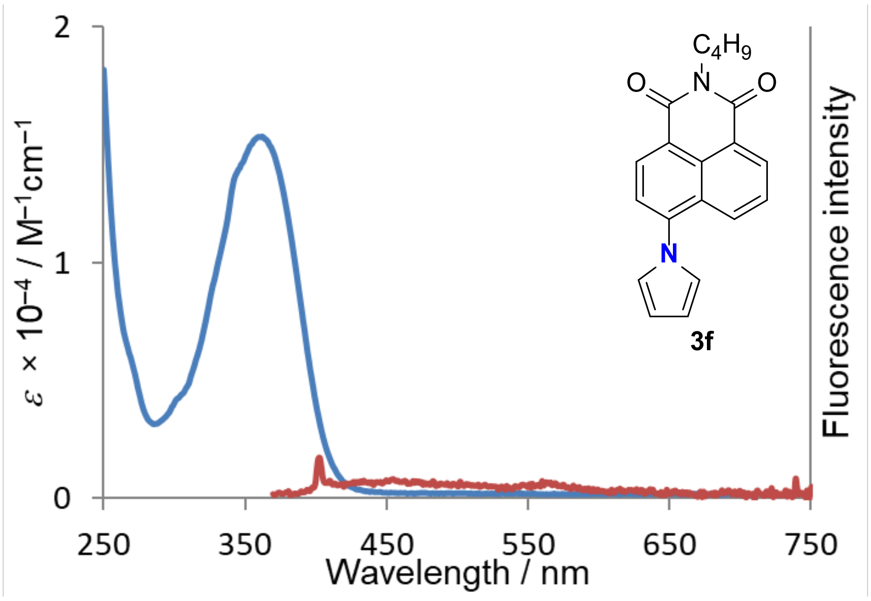

λ_{abs} = 360 nm
ε = 15400 $M^{-1}cm^{-1}$
log ε = 4.19
λ_{em} = n.d.
Φ_F = b.d.

Figure S6. Absorption (blue) and emission (red) spectra of **3f** measured for ethanol; n.d.- not determined; b.d.– below the detection limit.

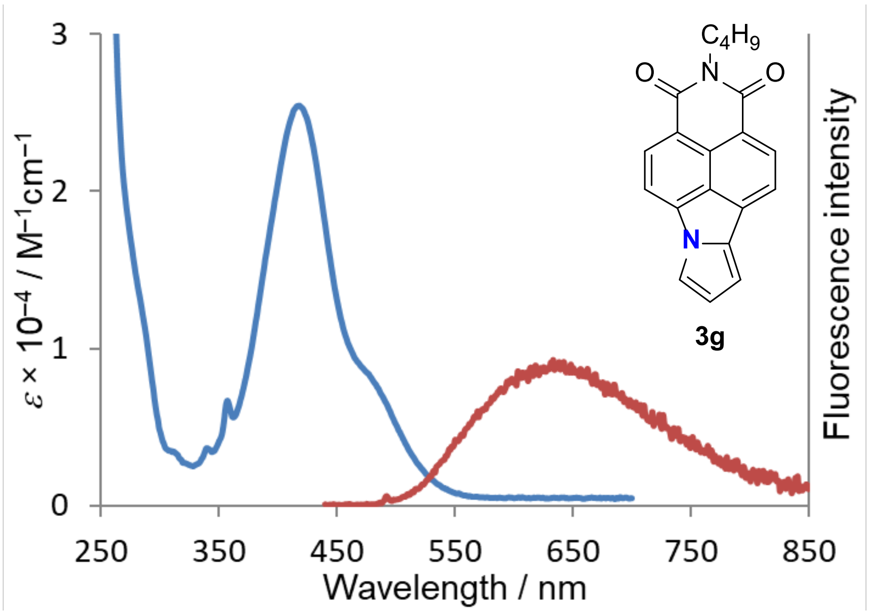

λ_{abs} = 418/465 nm
ε = 25500 / 9400 $M^{-1}cm^{-1}$
log ε = 4.40 / 3.97
λ_{em} = 633 nm
Φ_F = 0.009
$\varepsilon \times \Phi_F$ = 230 $M^{-1}cm^{-1}$

Figure S7. Absorption (blue) and emission (red) spectra of **3g** measured for ethanol.

4. Theoretical investigation

Methods

We have performed the DFT and TD-DFT calculations with Gaussian 16.[6] For all dyes the long alkyl chains have been replaced by methyl groups during the calculations for obvious computational reasons. Default Gaussian16 thresholds and algorithms were used but for an improved optimization threshold (10^{-5} au on average residual forces), a stricter self-consistent field convergence criterion (10^{-10} a.u.) and the use of the *ultrafine* DFT integration grid.

Firstly, the S_0 geometries have been optimized with DFT and the vibrational frequencies have been analytically determined, using the M06-2X *meta*-GGA hybrid exchange-correlation functional.[7] These calculations were performed with the 6-311G(d,p) atomic basis set and account for solvent effects through the linear-response PCM approach considering EtOH as solvent.[8] Secondly, starting from the optimal ground-state geometries, we have used TD-DFT with the same functional and basis set to optimize the S_1 geometry and compute the vibrational frequencies. All optimized structures correspond to true minima of the potential energy surface. Thirdly, the vertical transition energies were determined with TD-DFT and the same functional, but a larger basis set, namely 6-311+G(2d,p), in gas-phase as well as in solution using the cLR^2 variant of the PCM,[9] in its *non-equilibrium* limit.

As we are aware of the significant dependency of the TD-DFT results on the selected functional,[10] the obtained transition energies were also computed using CC2[11] and SCS-CC2[12] with the Turbomole 7.3 code.[13] The CC2 energies were calculated in gas phase applying the resolution of identity scheme, and using the *aug*-cc-pVTZ atomic basis set. Combining the CC2 and TD-DFT data using a well-known protocol,[14] one can obtain accurate CC2-corrected estimates of the absorption, emission and 0-0 energies that can be straightforwardly compared to experimental values. For the S-T gaps (determined only in gas phase without further corrections), we used SCS-CC2 rather than CC2 as this approach is known to be exceptionally efficient for these gaps.[15]

The SOC matrix elements were determined at the M06-2X/*def2*-TZVP level using ORCA.5.0.1[16] with ethanol as solvent as modelled with the SMD solvation model. The RIJCOSX method was used to accelerate the calculation, and DefGrid3 and TightSCF settings were applied, whereas TDA was turned off.

The vibrationally resolved spectrum were determined with the FCClasses 3.01 (v3-0.1-0177-g4b1514a) program.[17,18] We used both the time-dependent and time-independent formulations, applied the FC approximation (HT effects were neglected), and selected the so-called *Adiabatic Hessian* and *Vertical Hessian*[19] vibronic models for the calculations, the former being applied for **3a** only. We used a simulation temperature of 298K. For the TI part, the obtained stick spectra were convoluted with Gaussian having HWHM of 500 cm^{-1}. We found that 10^6 integral was sufficient to obtain a good convergence (FC recovery > 0.9 in the case of **3a**). In some cases, a few problematic vibrational modes had to be removed selectively to allow convergence of the TI expansion. Except when noted, the emission rates were obtained within the time-dependent formulation, the same FC approach and the *Vertical Hessian* model,[19] but we checked that the TI results were very similar. During the vibronic calculations, the vibrational contributions and transition dipoles are coming from TD-DFT, whereas the transition energies are CC2-corrected. The radiative and internal conversion rates have been obtained using the same methodology within the TVCF formalism.[20] For the radiative part, we used the same broadening as for the band shapes, i.e., 500 cm^{-1}, but this is known to be not important for the radiative rate.[20] For the IC part, we used a 10 cm^{-1} broadening Lorentzian, which is a typical value in the literature[21,22] though we are well aware of the difficulty to interpret such value.[21]

Density difference plots for 3a-3g

Below, we show the density difference plots for the excitation on all dyes. In all compounds one notes a significant asymmetry, characteristic of significant CT, especially in **3b**, **3e**, **3f** and **3g**.

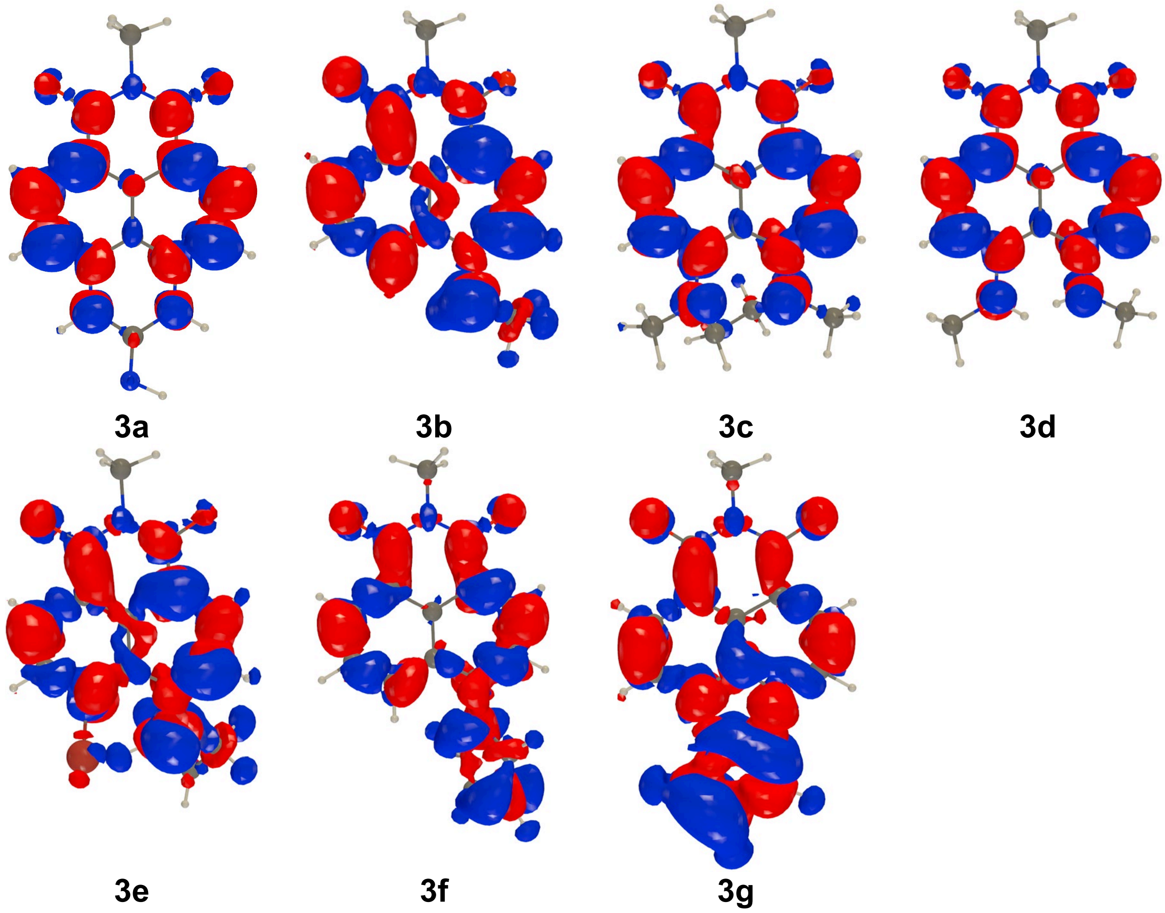

Figure S8. Density difference plot corresponding to the vertical absorption for all considered dyes. The blue and red lobes correspond to region of decrease and increase of electron density upon excitation, respectively. Contour threshold: 1 10^{-3}.

Charge transfer character 3a-3g

The above qualitative trends are confirmed by the CT parameters determined with Le Bahers' model. While in **3a**, **3c** and **3d**, the CT is mild with less than half of an electron transferred on less than 1.5 Å, the electron-hole separation becomes stronger in the four other compounds (ca. 2 Å, even 2.6 Å in **3g**), with larger amount of charge separation (>0.5 e).

Table S1. Charge transfer computed thanks to Le Bahers' model on the basis of the density difference plots. We report the CT distance representing the electron-hole separation at the FC point, as well as the amount of transferred charge.

Dye	d_{CT} (Å)	q_{CT} (e)
**3a**	1.182	0.428
**3b**	2.062	0.580
**3c**	1.370	0.461
**3d**	1.188	0.435
**3e**	1.949	0.576
**3f**	2.168	0.522
**3g**	2.591	0.689

Possible quenching through TICT

Given the push-pull nature of the dyes and the presence of amino groups able to rotate in **3b**, **3d**, **3e**, and **3f**, we have investigated the possibility of twisted intramolecular charge transfer (TICT). For **3a, 3c,** and **3g**, such mechanism is obviously not possible due to the geometrical constrains imposed by the donor groups used. To probe TICT, we first performed TD-DFT optimization

starting with the amino group orthogonal (90° twist) as compared to the core of the dye. In a second step, we determined the excited-state energy with the protocol described above, i.e., we account for both *linear-response* and *state-specific* solvent effects, and include also CC2 corrections.

For **3b**, the geometry optimization led back to the standard planar form, indicating that TICT is not a possible process according to the selected level of theory. This is consistent with the large emission quantum yield measured experimentally.

For **3d, 3e**, and **3f**, we could locate TICT like excited-state geometries that are represented in **Figure S9**. Note that in **3d**, only one of both the donating amino groups can be positioned in a TICT configuration, the former approach leading to a favorable interaction between the hydrogen atom of the (now planar) amino group and the lone pair of the vicinal (TICT) nitrogen.

The relative S_1 energies are as follows: in **3d**, the single (double) TICT structure is more stable than the canonical form by -0.16 (-0.25) eV; in **3e**, the TICT conformer is more stable than the canonical one by -0.33 eV; and in **3f**, the difference becomes very large, namely -0.65 eV. One can therefore conclude that TICT is energetically possible in these three compounds. At this stage we wish however to mention an important point. In the single TICT **3d** conformer, the transition remains bright (large oscillator strength of 0.35), whereas in the double TICT conformation, the transition becomes totally dark (oscillator strength < 0.001). In both **3e** and **3f**, the TICT structures are also showing almost forbidden S_1-S_0 transition (oscillator strength of 0.00 and 0.02, respectively), indicating that all TICT structures will not emit significantly in solution (lack of radiative coupling), but the single-TICT **3d**.

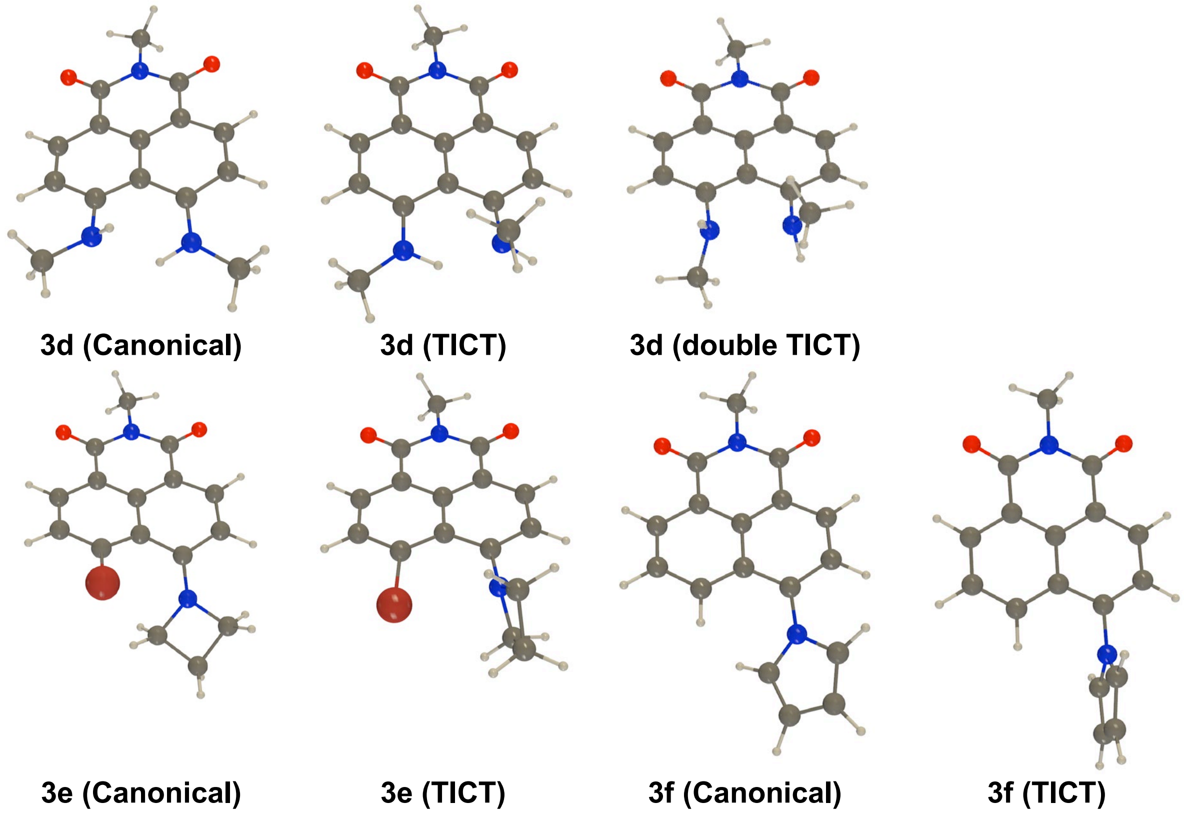

Figure S9. Optimal geometries of the excited-states of **3d**, **3e**, and **3f** in their canonical and TICT minima.

After having shown that the TICT structure is energetically favored for all three compounds displayed in **Figure S9**, we have also explored the pathway connecting the canonical and twisted conformations, by freezing performed a relaxed scan around the dihedral angle connecting the core of the dye and the amino donor by step of 15°. The results are displayed in **Figure S10**.

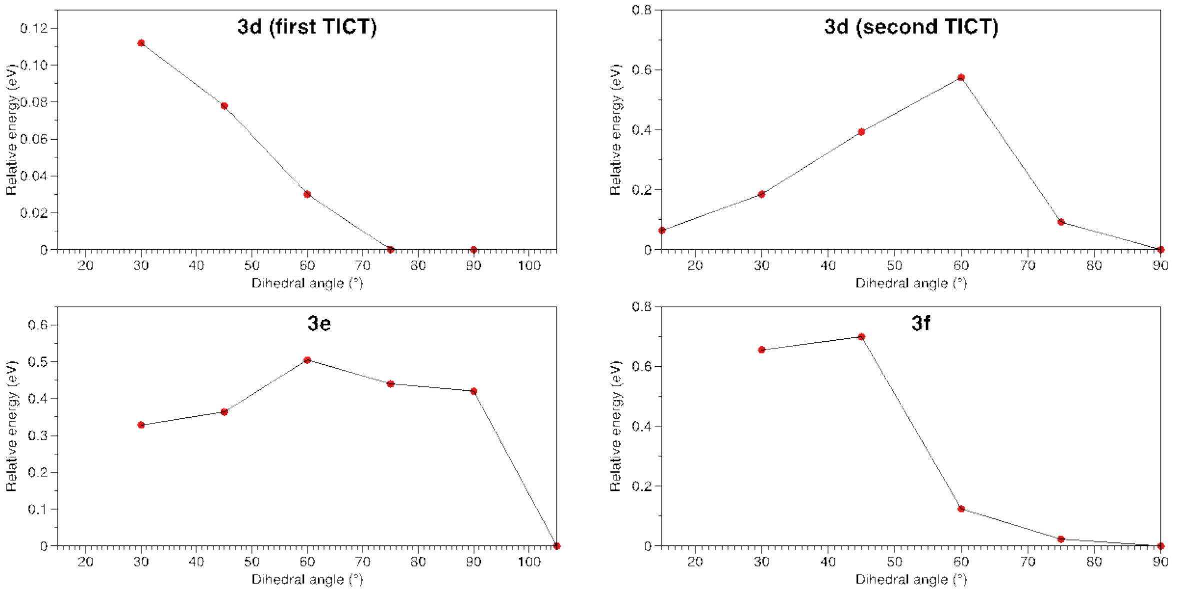

Figure S10. Relaxed energy scan corresponding to TICT (15° step). The most stable species is taken as reference. Note the different X and Y scales.

3d is an interesting case: the first TICT process is totally barrierless, i.e., the canonical structure is unstable in the excited state. This leads to the structure displayed in **Figure S9**, with a strong H-bond between the two NHMe group. This explains why the second TICT process is more difficult (barrier of 0.54 eV, see **Figure S10**) despite the fact that the double TICT structure is the most stable. In that case, one can thus consider that a significant population of **3d** will emit from the (single) TICT structure, consistent with the experimental observation of a reasonably bright emission and rather larger Stokes shift. However, due to the presence of a lot of excess vibrational energy in the excited state, one cannot discard that a fraction of the population will undergo the second TICT leading to a dark excited state.

In **3e**, the presence of the large bromine atom and the rigid four-member cycles induces significant steric clash, and it is only when the twisting becomes very large that the TICT form becomes more stable than the canonical one. However, the potential energy surface is rather flat and the barrier for TICT remains quite small (0.17 eV) for an excited state process in which the vibrational energy is typically very large after photon absorption. It is therefore likely that TICT is an efficient process in **3e**.

It can be seen that in **3f**, the barrier for the TICT is very small (0.04 eV), and it means that in practice the conversion from the canonical to the twisted form is essentially barrierless. In other words, in **3f**, the primary photophysical process after absorption is TICT and no emission will be observed.

Intersystem crossing

Given the presence of bromine in one of the dye, we have also investigated the possibility of intersystem crossing. To this end, we computed S-T gaps at the SCS-CC2/*aug*-cc-pVTZ and the SOC coupling elements at the TD-DFT level. The key results are listed in **Table S2**. For all dyes, only one triplet is below the lowest singlet at the optimal S_1 geometry. The gaps are globally very large (>0.6 eV) and the spin-orbit couplings are well below 1 cm^{-1}, a combination which indicates a very limited efficiency of ISC. There is one, expected, exception to this trend, namely the bromine-bearing **3e**. This compound is still characterized by a large S-T gap (0.651 eV), but much larger SOC matrix elements, which hint at possible and maybe even efficient ISC. Note that even more efficient ISC could be possible after TICT for **3e** but this would not change the conclusion of efficient quenching of the fluorescence.

Table S2. SCS-CC2 singlet-triplet gap (in eV) computed on the optimal (canonical except when noted) S_1 geometry and SOC matrix elements (X/Y/Z, in cm-1) determined on the same geometry

Dye	S_1-T_1 gap (eV)	SOCme X/Y/Z (cm^{-1})
**3a**	0.841	0.00/0.00/0.00
**3b**	0.718	0.01/-0.02/0.00
**3c**	0.742	0.00/0.03/0.03
**3d (canonical)**	0.722	0.01/0.05/-0.10
**3d (TICT)**	0.629	0.01/0.07/0.15
**3e**	0.651	-1.08/-0.62/-5.84
**3f**	1.032	0.04/0.13/-0.16
**3g**	0.676	-0.02/-0.00/0.00

Band shapes, radiative and internal conversion rates

Having discarded dyes **3e** and **3f** as non-emissive, we have considered the remaining five dyes and modelled their vibrationally resolved spectra, considering the single TICT form for **3d**. Let us first start with **3a.** This dye is obviously extremely rigid, and it allows some methodological testing. Our results are displayed in **Figure S11** and **Table S3**. For this rigid system, one can clearly notice, the good agreement between all four vibronic approaches (TD-AH, TD-VH, TI-AH, and TI-VH) with globally similar band shapes (fitting the experimental ones) and alike photophysical parameters. In the following, we have therefore continued with the TD-VH model mainly, systematically checking the agreement with the TI-VH results. This choice of VH rather than AH is dictated by the nature of most other dyes with rather large CT character (and significant Stokes shift) making it challenging to properly converge AH calculations.

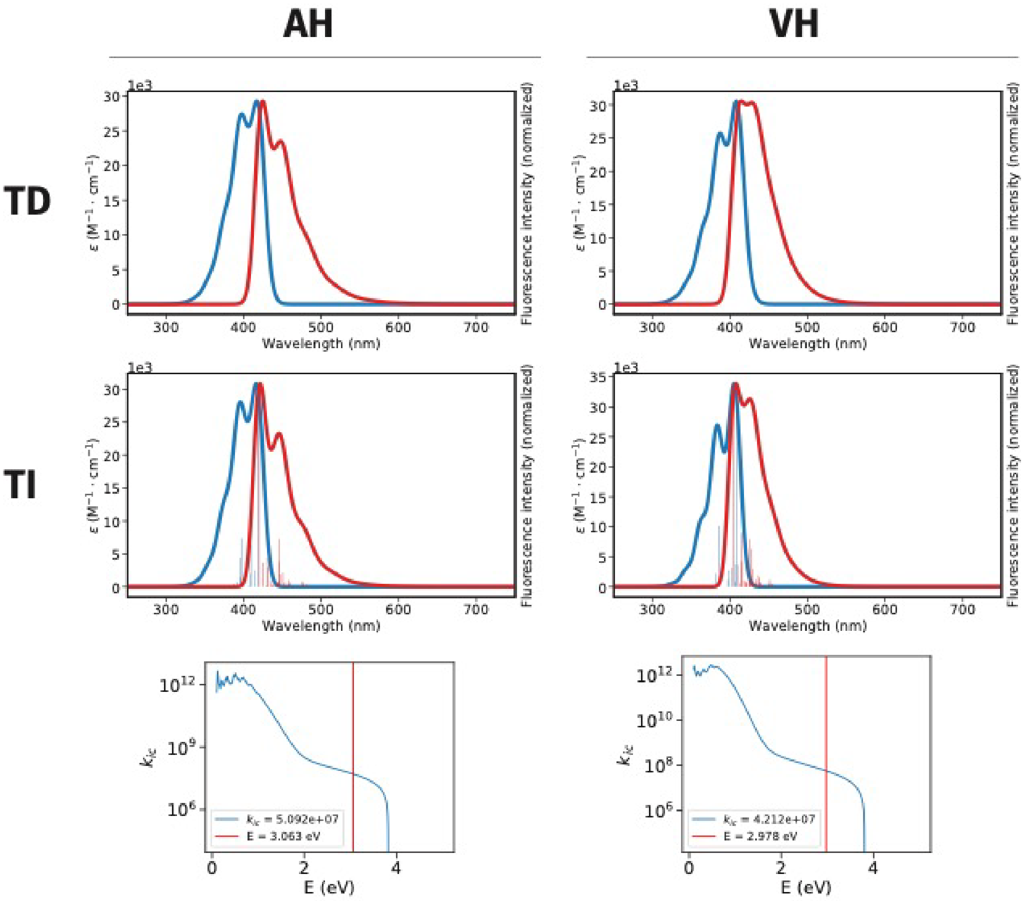

Figure S11. Analysis for **3a.** Top: vibrationally resolved spectra computed in the TD approach. Center: same spectra determined in the TI approach, note the limited number of significant "stick" contributions, the FC recovery exceeds 0.95 in all cases. Bottom: plot relating the energy to the k_{ic}, showing the expected smooth behavior, the redline being the reference energy used in the calculation. The left and right graphs correspond to the AH and VH vibronic models, respectively.

Table S3. Theoretical photophysical data obtained for **3a**.

Model	log (ε)	k_r (10^8 s^{-1})	k_{ic} (10^8 s^{-1})	Φ_F
**TD-AH**	4.47	1.27	0.51	0.71
**TD-VH**	4.48	1.40	0.42	0.77
**TI-AH**	4.49	1.29	0.51	0.72
**TI-VH**	4.53	1.44	0.42	0.77

Let us now turn to the other systems. For **3b** and **3c**, the analysis is straightforward. As can be seen in **Figure S12,** theory nicely reproduces the large CT-band shape with large Stokes shift for **3b**, and the tight band topology with a clear shoulder for **3c**. The epsilons are also in good match with the measurements, as are the orders of magnitude of the emission quantum yields. For **3d**, there is a notable asymmetry between the absorption and emission bands, a phenomenon also noted experimentally and related to the TICT rearrangement explained above. For **3g**, one notes broad bands, typical of strong CT. The molar extinction coefficient is also much smaller than in the other dyes, consistent with the measurements.

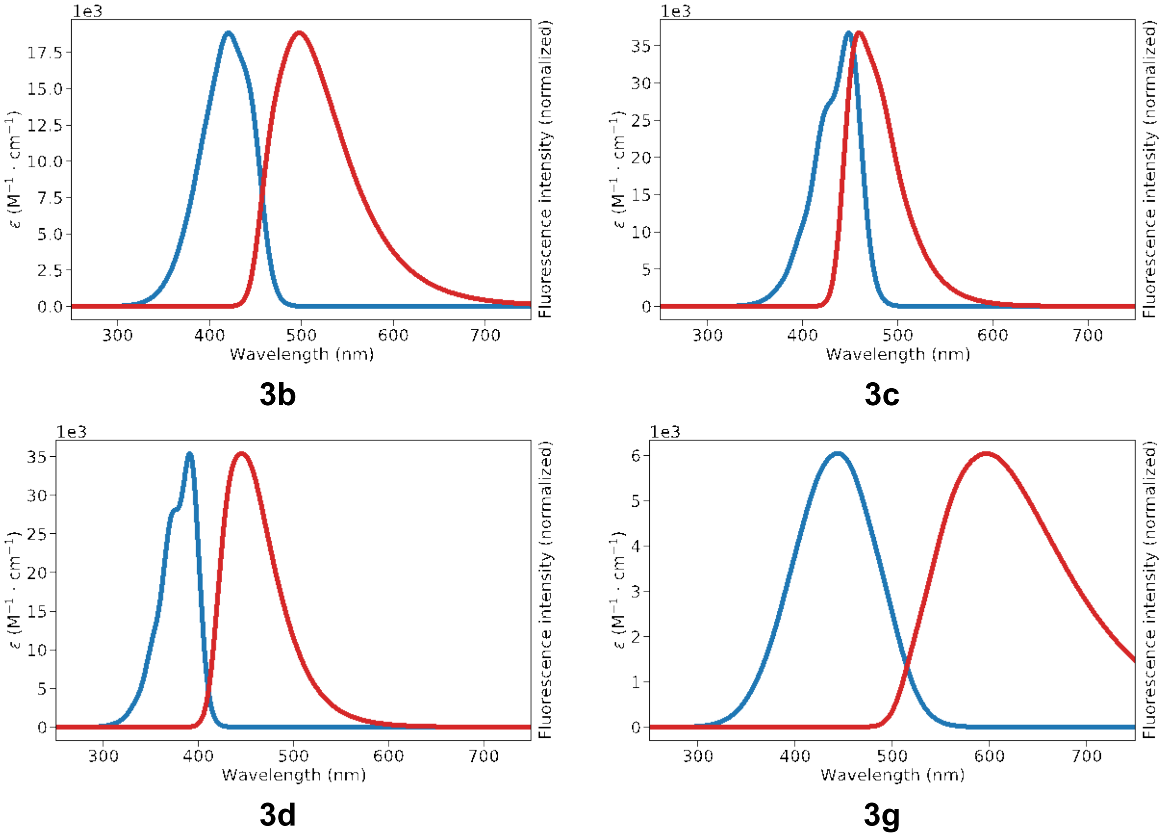

Figure S12. TD-VH absorption and emission spectra computed for **3b**, **3c**, **3d** (at the TICT geometry for emission) and **3g**.

Table S4. Theoretical photophysical data obtained for the five emissive dyes. All results obtained in the TD-VH approach. For **3d**, the emissive properties are determined from the bright TICT structure whereas the molar extinction coefficient on the ground-state geometry.

Dye	log (ε)	k_r (10^8 s^{-1})	k_{ic} (10^8 s^{-1})	Φ_F
**3a**	4.48	1.40	0.42	0.77
**3b**	4.27	0.66	1.01	0.40
**3c**	4.57	1.24	0.65	0.66
**3d**	4.55	1.03	0.67	0.61
**3g**	3.78	0.23	2.69	0.08

5. Biological studies

Enzymes preparation

Prothrombin obtained from fresh frozen plasma was purified by a modified method of Bajzar and colleagues.[23] Thrombin was prepared by activation of prothrombin with *Echis Carinatus* snake venom as described.[24] Protein purity was confirmed using SDS-polyacrylamide gel electrophoresis (SDS-PAGE) according to the methods described by Laemmli.[25]

Activity-based probe synthesis

In the first step 100 mg of 2-chlorotrityl resin (for each activity-based probe) was placed in the peptide synthesis vessel and swollen in anhydrous CH_2Cl_2 for 30 min, and then, the resin was washed once with CH_2Cl_2. Next, Fmoc-P2-OH (2.5 equiv) was dissolved in anhydrous CH_2Cl_2, preactivated with DIPEA (3 equiv, 84 µL), added to the resin under an argon atmosphere, and stirred gently for 12 h. After that, the mixture was filtered, the resin was washed with CH_2Cl_2 (three times), and the remaining active sites on the 2-chlorotrityl resin were deactivated with CH_2Cl_2/MeOH/DIPEA (%, v/v/v, 80:15:5) solution, for 1 h. Next, the mixture was filtered, the resin was washed with DMF (six times), and N-terminus Fmoc-protecting group deprotection was performed using 20% piperidine in DMF (5, 5, and 25 min). After the Fmoc-protecting group removal, the P3, P4 amino acids, and Fmoc-Ahx-OH were attached to the H_2N-P2-resin with HOBt (2.5 equiv, 60 mg), and DICI (2.5 equiv, 52 µL) as coupling reagents in coupling/deprotection cycles. The biotin/fluorescent tag was coupled to H_2N-Ahx-P4-P3-P2-resin using HATU (2.5 equiv, 152 mg), and 2,4,6-collidine (2.5 equiv, 53 µL), as coupling reagents in a DMF/DMSO mixture (%, v/v, 50:50). After 3 h, the resin was washed with DMF (six times), CH_2Cl_2 (three times), MeOH (three times), and dried over P_2O_5. The crude peptide was cleaved from the resin with a mixture of CH_2Cl_2/TFE/AcOH (%, v/v/v, 80:10:10). The solution was filtered, concentrated, and lyophilized. Next, the reactive warhead, diphenyl phosphonate (Cbz-Arg$(Boc)_2{}^P(OPh)_2$) was synthesized according to a previously described methodology.[26] The Cbz-protecting group was removed using hydrogen and palladium on carbon. The obtained H_2N-Arg$(Boc)_2{}^P(OPh)_2$ (1 equiv) was coupled with a peptide sequence tag-6-Ahx-P4-P3-P2-COOH (1.2 equiv), with HATU (1.2 equiv) and 2,4,6-collidine (4 equiv). The reaction was monitored with analytical HPLC and after 2 h the product was extracted to ethyl acetate with 5% $NaHCO_3$, 5% citric acid, and brine. The organic phase was collected, dried over $MgSO_4$, and evaporated. Finally, the side chain amino acid protecting groups were removed with a mixture of TFA/CH_2Cl_2/TIPS (%, v/v/v, 80:15:5). After 30 min solvents were removed with argon flow, and the obtained product (tag-6-Ahx-P4-P3-P2-$Arg^P(OPh)_2$) was dissolved in peptide grade DMSO. All activity-based probes were purified by HPLC on a Waters M600 solvent delivery module with a Waters M2489 detector system using a semipreparative Wide Pore C8 Discovery column (Waters sp z.o.o., Warszawa, Poland). The solvent composition was as follows: phase A (water:0.1% TFA) and phase B (acetonitrile:0.1% TFA). The purity of each compound was confirmed with an analytical HPLC system using a Discovery Bio Wide Pore C8 analytical column. The solvent composition was as follows: phase A (water:0.1% TFA) and phase B (acetonitrile:0.1% TFA); gradient, from 95% A to 5% A over a period of 15 min. The purity of all compounds was ≥ 95%. The molecular weight of each compound was confirmed using a WATERS LC-MS with Acquity QDa Mass Detector. Each activity-based probe was then dissolved in peptide grade DMSO to a final concentration of 10 mM and stored at –80 °C until use.

Inhibition of thrombin by biotinylated and fluorescent activity-based probes

Kinetic experiments were performed using a spectrofluorometer (Molecular Devices SpectraMax Gemini XPS) on 96-well plates (Corning) with excitation/emission wavelength, 355/460 nm (cutoff, 455 nm). The assay buffer contained 20 mM Tris-base, 150 mM NaCl, 5 mM $CaCl_2$, pH 7.4. Each activity-based probe was diluted to obtain the concentrations ranging from 10 to 1000 nM. The assay conditions were as follows: 40 µL of thrombin (10 nM) was added into the wells containing

40 µL of four different activity-based probes concentrations. The mixture was incubated for 30 min at 37 °C and then to each well 20 µL of selected substrate (100 µM SMA4, Ac-Lys-Nle(*O*-Bzl)-Lys(2-Cl-Z)-Arg-ACC) was added. As a control, enzyme without probes was tested. Thrombin activity was measured for 30 min and the linear portion of each kinetic curve was used for analysis. Obtained results were analyzed using SoftMax (Molecular Devices), GraphPad Prism, and Microsoft Excel software. The data represent the mean values ± s.d.; $n = 3$, where n is the number of independent experiments.

Thrombin labeling by activity-based probe based on SDS-PAGE analysis

Purified thrombin (200 nM) was incubated with varying probe concentrations ranging from 50 to 1600 nM in assay buffer (20 mM Tris-base, 150 mM NaCl, 5 mM $CaCl_2$, pH 7.4) for 30 min at 37 °C. Thrombin was incubated with probe in a total volume of 40 µL (20 µL of enzyme and 20 µL of probe), followed by the reduction with 20 µL of 3 × SDS/DTT for 5 min at 95 °C. The first well was loaded with 0.5 µL of the protein marker PageRuler Plus Prestained Protein Ladder (Thermo Scientific), then 10 µL of each sample was run onto a 12% (w/v) 15-well gel. The SDS-PAGE separation was performed at 200 V for 39 min. The gel was then directly scanned at 488 nm using an Azure Biosystems Sapphire Biomolecular Imager and Azure Spot Analysis Software.

Kinetic studies

Kinetic experiments were performed using a spectrofluorometer (Molecular Devices SpectraMax Gemini XPS) on 96-well plates (Corning) with excitation/emission wavelength, 355/460 nm (cutoff, 455 nm). The assay buffer contained 20 mM Tris-base, 150 mM NaCl, 5 mM $CaCl_2$, pH 7.4.

Inhibition of thrombin by biotinylated and fluorescent activity-based probes

Each activity-based probe was diluted to obtain the concentrations ranging from 10 to 1000 nM. The assay conditions were as follows: 40 µL of thrombin (10 nM) was added into the wells containing 40 µL of four different activity-based probes concentrations. The mixture was incubated for 30 min at 37 °C and then to each well 20 µL of selected substrate (100 µM SMA4, Ac-Lys-Nle(*O*-Bzl)-Lys(2-Cl-Z)-Arg-ACC)[27] was added. As a control, enzyme without probe was tested. Thrombin activity was measured for 30 min and the linear portion of each kinetic curve was used for analysis. The residual activity was established by setting the RFU/s value of control as 100% and adjusting other results accordingly. Obtained results were analyzed using SoftMax (Molecular Devices), GraphPad Prism, and Microsoft Excel software. The data represent the mean values ± s.d.; $n = 3$, where n is the number of independent experiments.

Determination of inhibition kinetics (IC_{50}) for biotinylated and fluorescent activity-based probes

Each activity-based probe was serially diluted to obtain concentrations ranging from 0.15 nM to 1600 nM, depending on the activity-based probe used. The assay conditions were as follows: 40 µL of thrombin (10 nM) was added into the wells containing 40 µL of different activity-based probes concentrations. The mixture was incubated for 30 min at 37 °C and then to each well 20 µL of selected substrate (100 µM SMA4, Ac-Lys-Nle(*O*-Bzl)-Lys(2-Cl-Z)-Arg-ACC)[27] was added. As a control, enzyme without probe was tested. The fluorescence increase over time was measured for 30 min and the linear portion of each kinetic curve was used for analysis. Obtained results were analyzed using SoftMax (Molecular Devices). The IC_{50} values were calculated using GraphPad Prism and Microsoft Excel software. The data represent the mean values ± s.d.; $n = 3$, where n is the number of independent experiments.

Table S5. Kinetic parameters (IC_{50}) of the biotinylated and fluorescent activity-based probes determined in the presence of thrombin. The data represent the mean values ± s.d.; *n* = 3, where *n* is the number of independent experiments.

	Activity-based probe		
	**5a**	**5b**	**5c**
**IC_{50} [nM]**	60.9 ± 1.7	14.6 ± 0.5	70.2 ± 2.7

Thrombin labeling by activity-based probe based on SDS-PAGE analysis

Purified thrombin (200 nM) was incubated with varying probe concentrations ranging from 25 to 1200 nM in assay buffer (20 mM Tris-base, 150 mM NaCl, 5 mM $CaCl_2$, pH 7.4) for 30 min at 37 °C. Thrombin was incubated with probe in a total volume of 40 μL (20 μL of enzyme and 20 μL of probe), followed by the reduction with 20 μL of 3 × SDS/DTT for 5 min at 95 °C. When testing the inhibitor utility, 200 nM of thrombin was incubated with inhibitor (10 μM I-SMII55, Ac-Nle-Lys-Pro-$Arg^P(OPh)_2$)[27] for 60 min prior to probe addition. The first well was loaded with 0.5 μL of the protein marker PageRuler Plus Prestained Protein Ladder (Thermo Scientific), then 10 μL of each sample was run onto a 12% (w/v) 15-well gel. The SDS-PAGE separation was performed at 200 V for 39 min. The gel was then directly scanned at 488 nm using an Azure Biosystems Sapphire Biomolecular Imager and Azure Spot Analysis Software. SDS-PAGE analysis was performed using two activity-based probes (**5a** and **5b**). The optimum fluorescence signal was obtained using probe **5a**, therefore it was used to determine the detection limit.

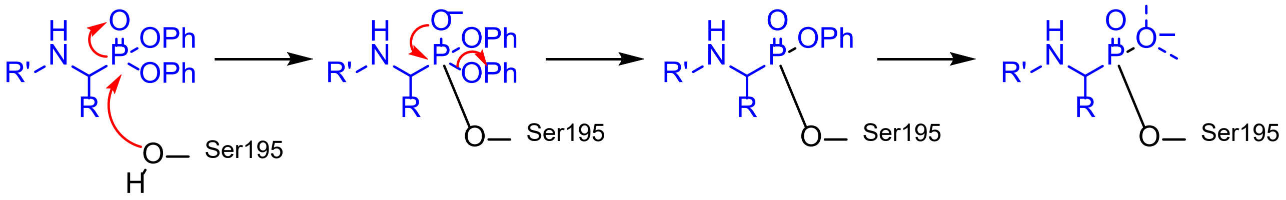

Scheme S6. Mechanism of serine protease inhibition by diphenyl phosphonate ester. Inhibition of activity occurs by formation of a covalent bond involving the hydroxyl group of the Ser195 residue (chymotrypsin numbering).[28]

Purity and MS analysis of activity-based probes

5a, 4a-6-Ahx-Nle-Lys-Pro-Arg^{P}(OPh)$_2$

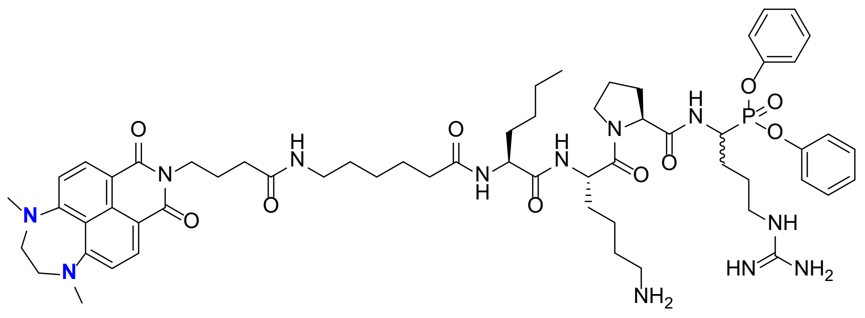

Chemical Formula: $C_{60}H_{83}N_{12}O_{10}P$
Exact Mass: 1162,6093
Molecular Weight: 1163,3718

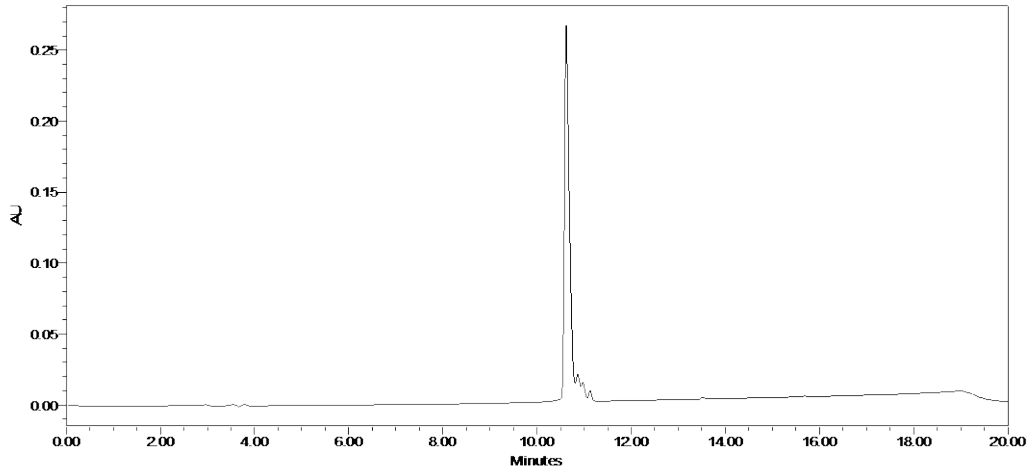

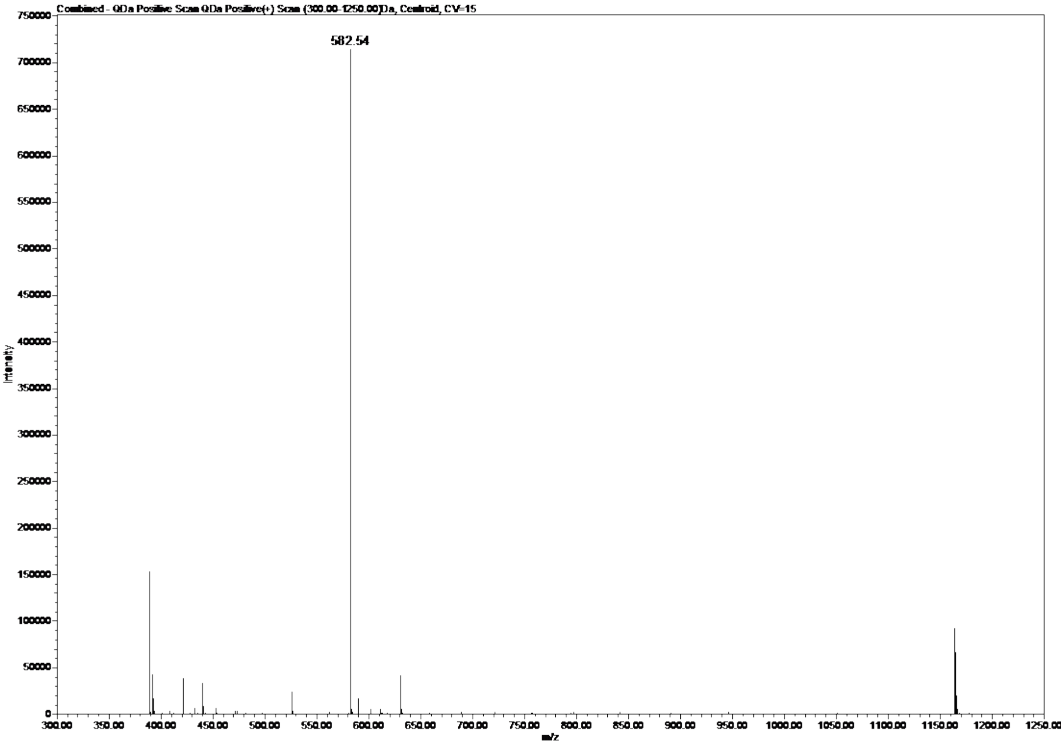

Figure S13. Analytical chromatogram for activity-based probe **5a** (top) LC-MS analysis for activity-based probe **5a** (bottom).

5b, 4b-6-Ahx-Nle-Lys-Pro-Arg^{P}(OPh)$_2$

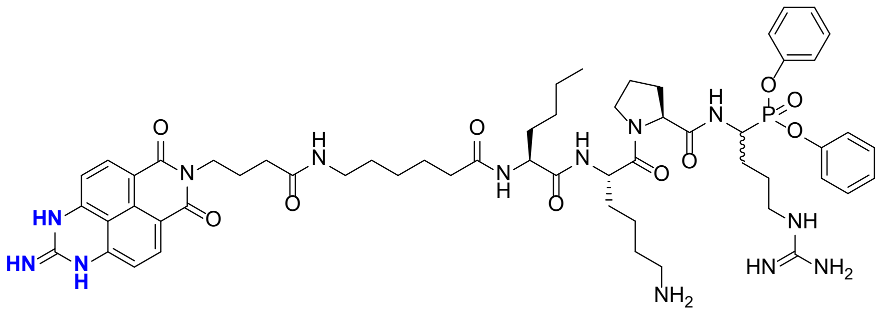

Chemical Formula: $C_{57}H_{76}N_{13}O_{10}P$
Exact Mass: 1133,5576
Molecular Weight: 1134,2898

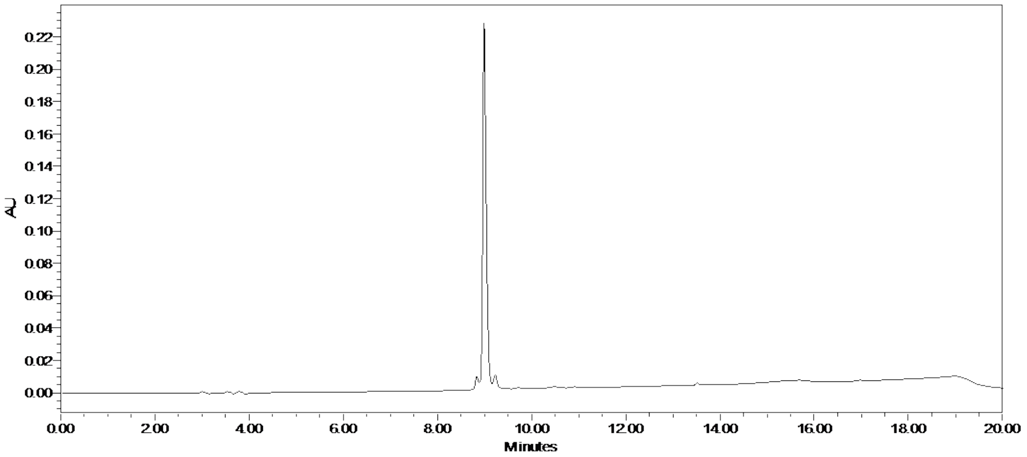

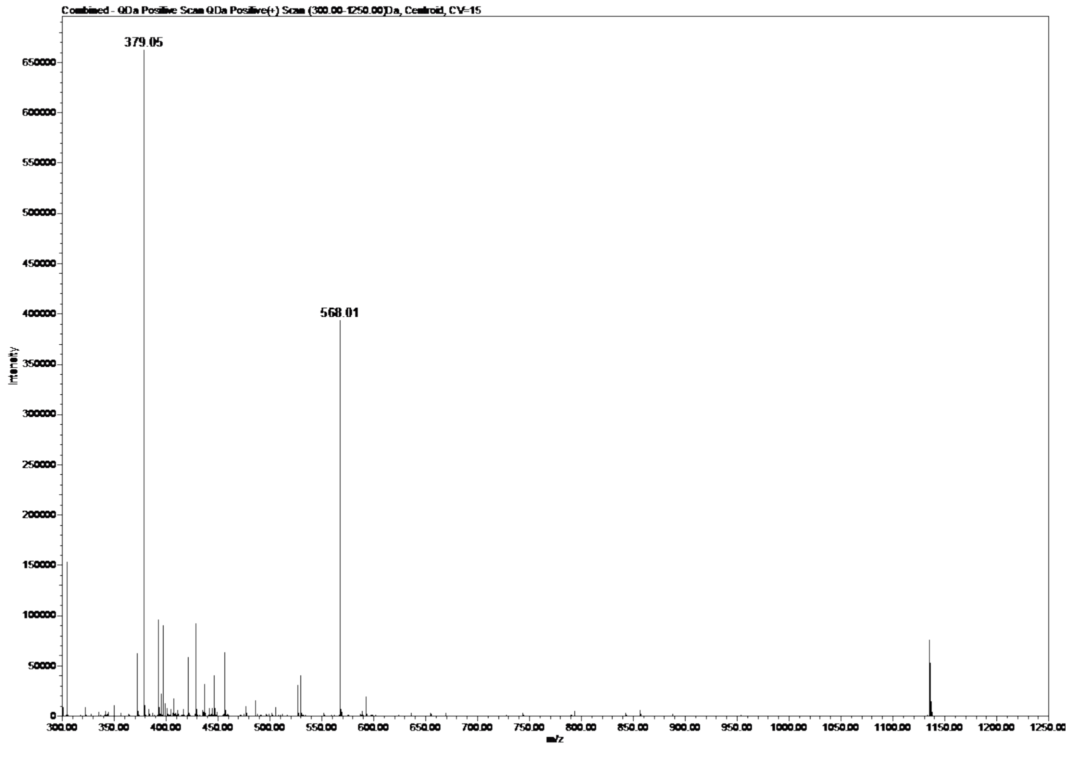

Figure S14. Analytical chromatogram for activity-based probe **5b** (top) LC-MS analysis for activity-based probe **5b** (bottom).

5c, biotin-6-Ahx-Nle-Lys-Pro-Arg^{P}(OPh)$_2$

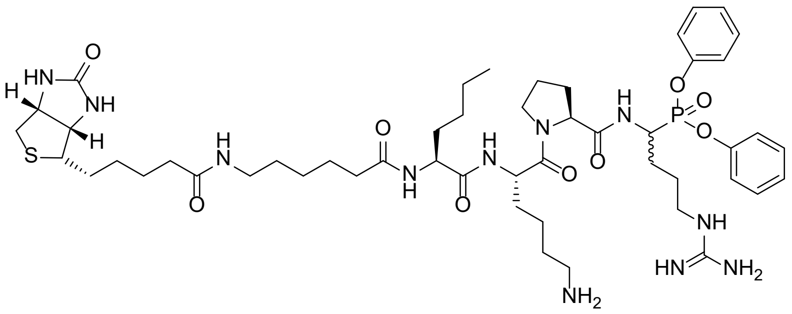

Chemical Formula: $C_{50}H_{78}N_{11}O_{9}PS$
Exact Mass: 1039,5442
Molecular Weight: 1040,2758

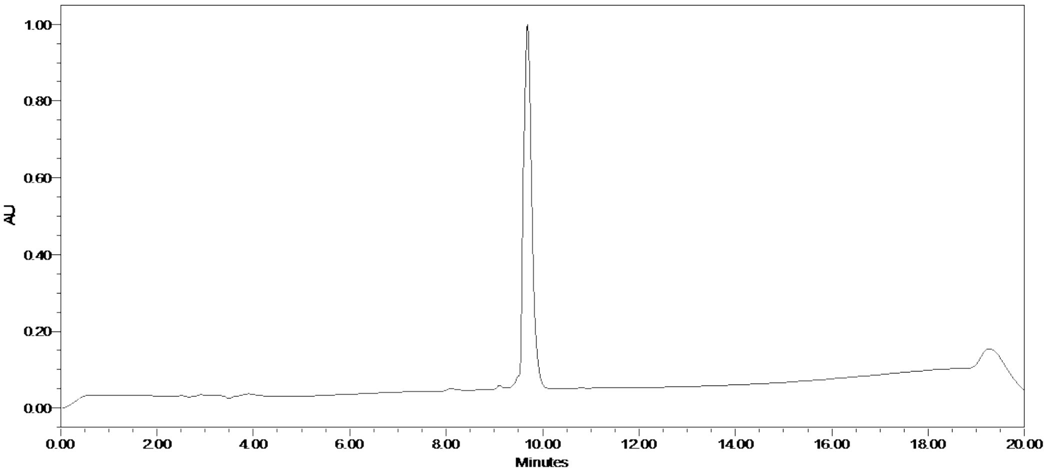

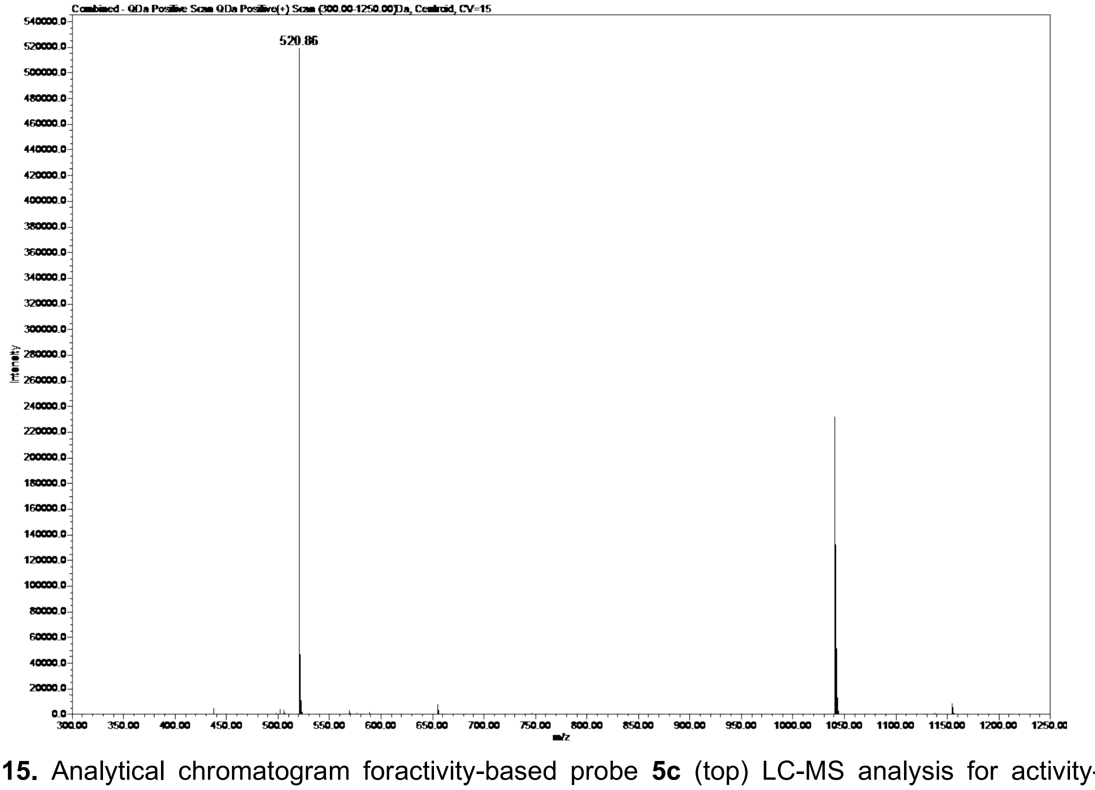

Figure S15. Analytical chromatogram foractivity-based probe **5c** (top) LC-MS analysis for activity-based probe **5c** (bottom).

6. ^{1}H and ^{13}C NMR spectra for synthesized compounds

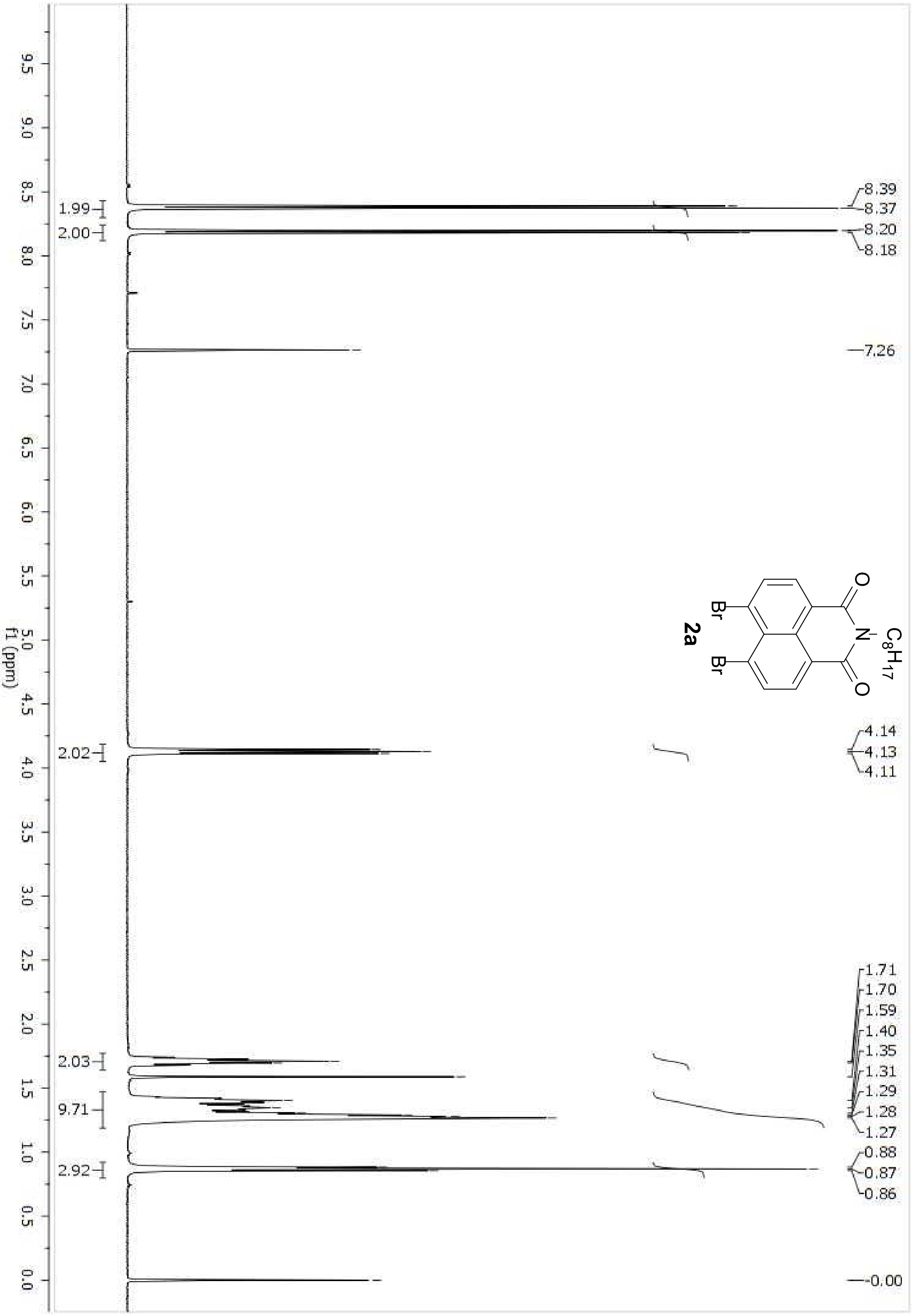

Figure S16. ^{1}H NMR spectrum of **2a** (500 MHz, $CDCl_3$).

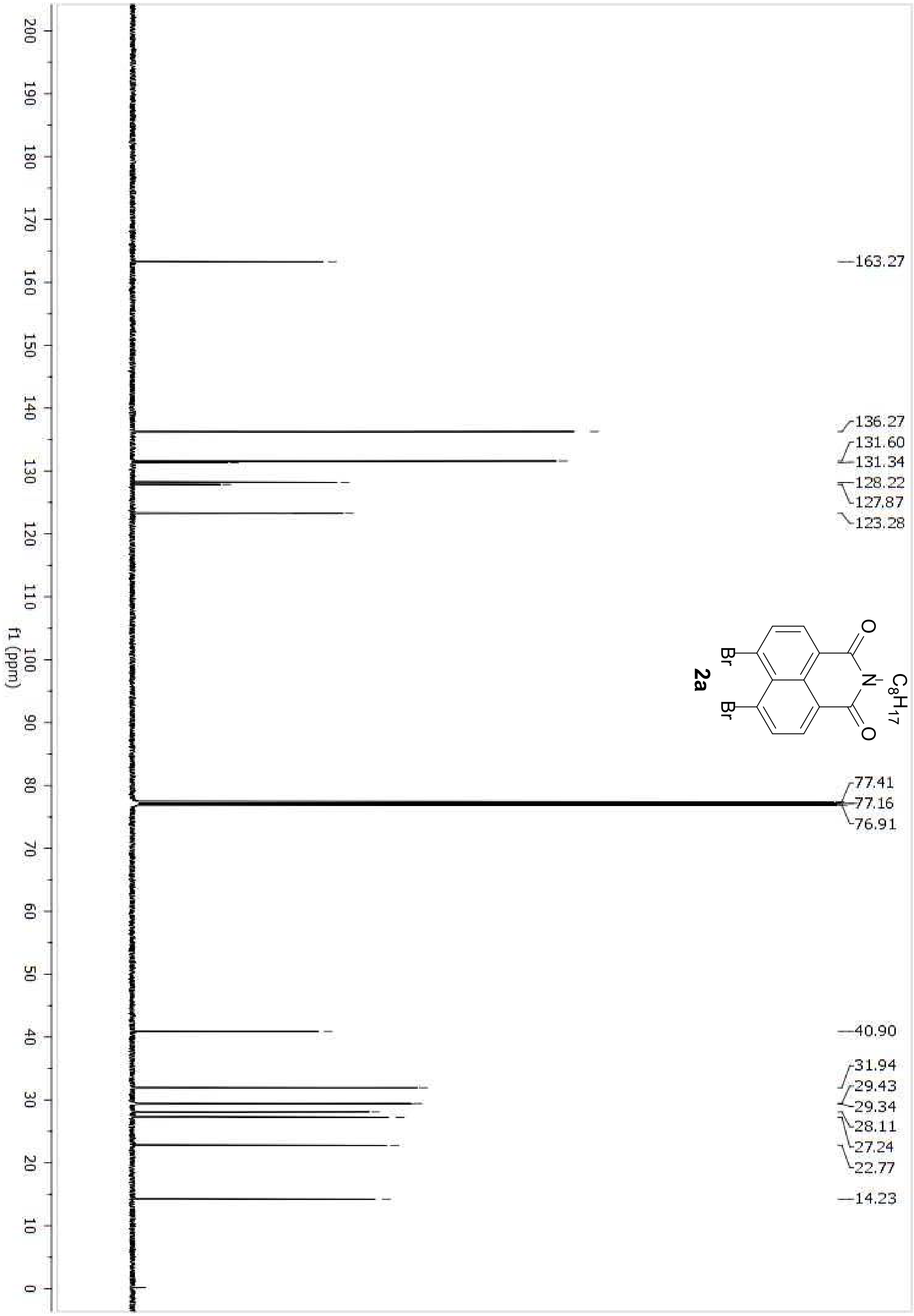

Figure S17. ^{13}C NMR spectrum of **2a** (126 MHz, $CDCl_3$).

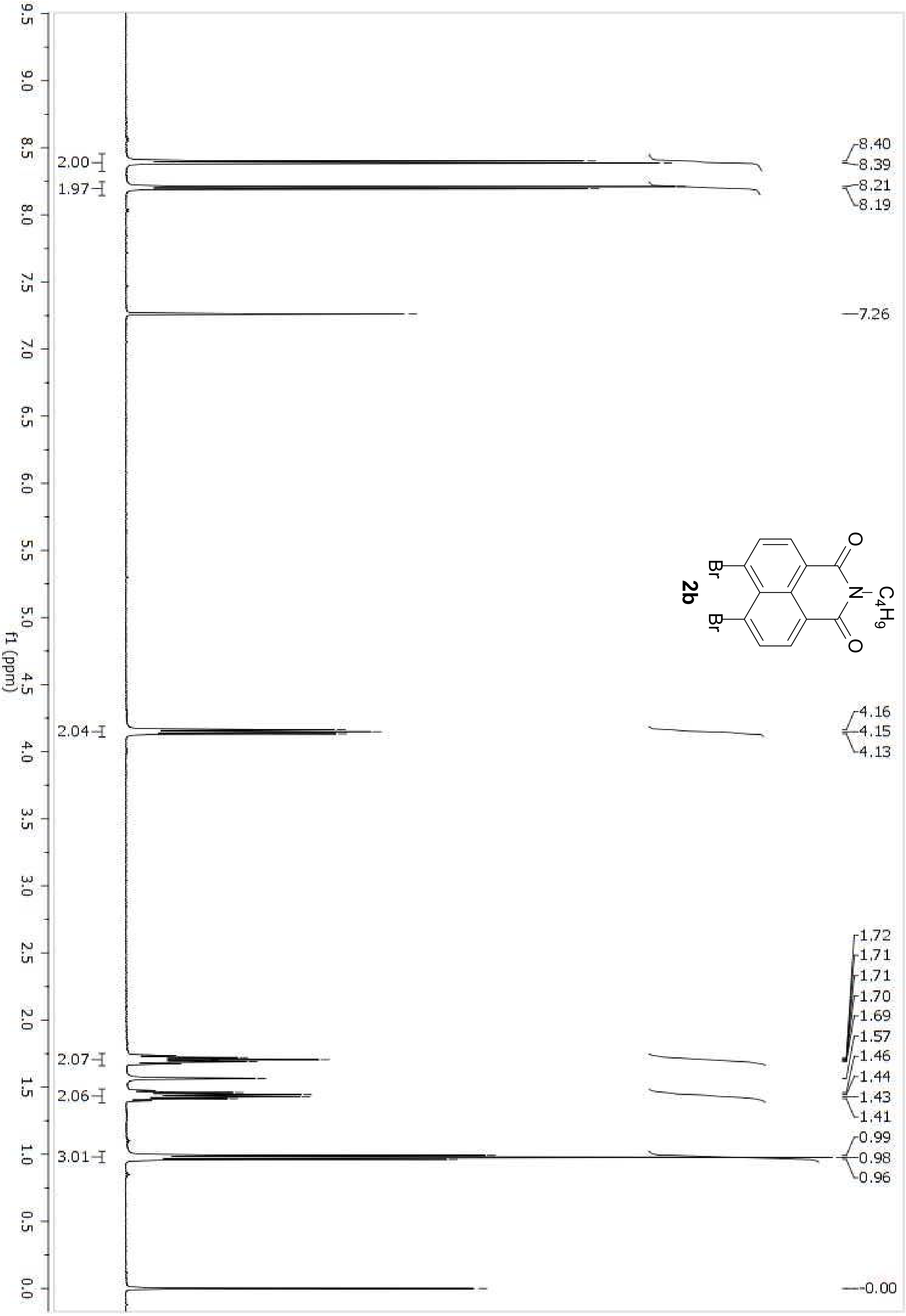

Figure S18. ^{1}H NMR spectrum of **2b** (500 MHz, $CDCl_3$).

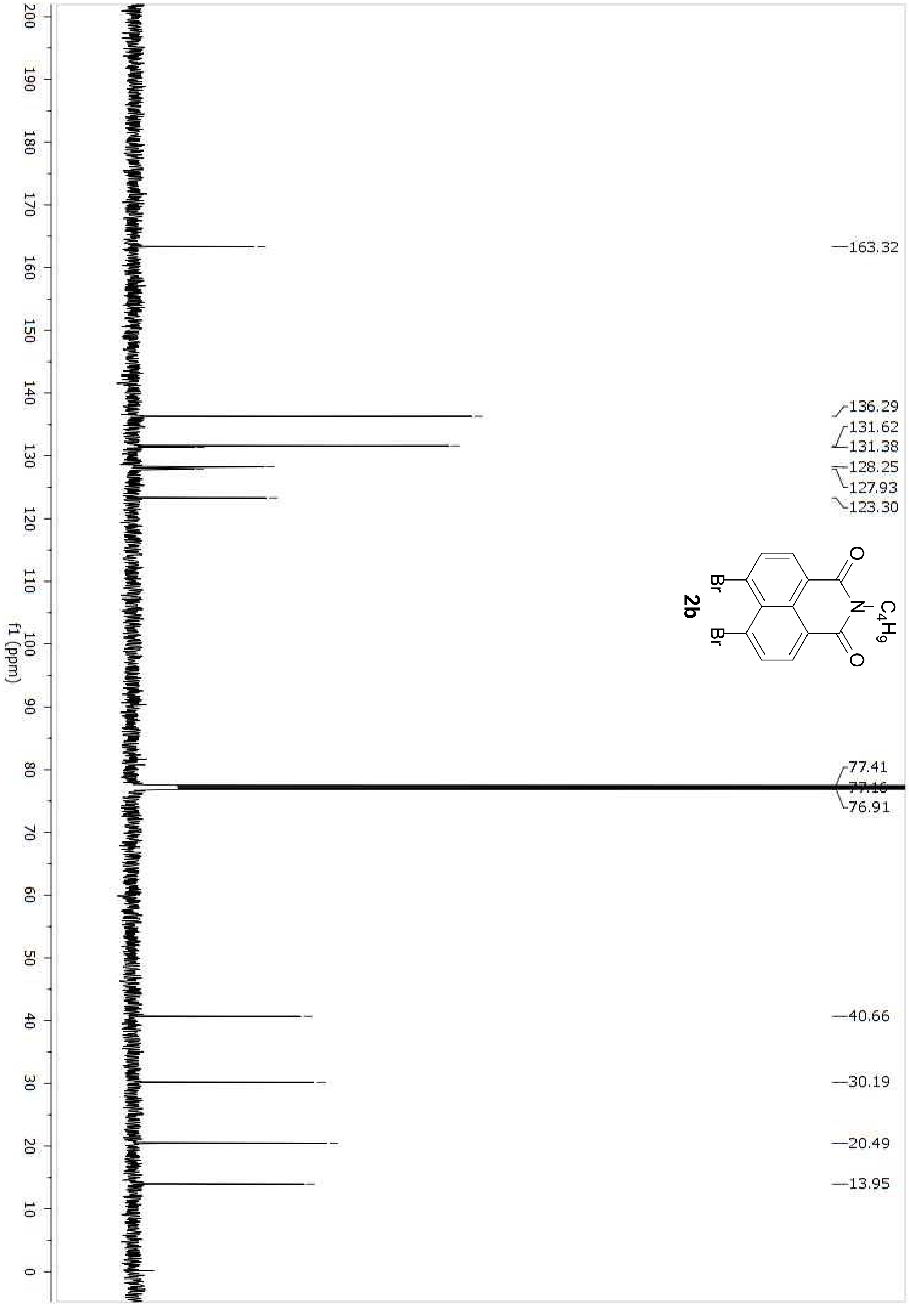

Figure S19. ^{13}C NMR spectrum of **2b** (126 MHz, $CDCl_3$).

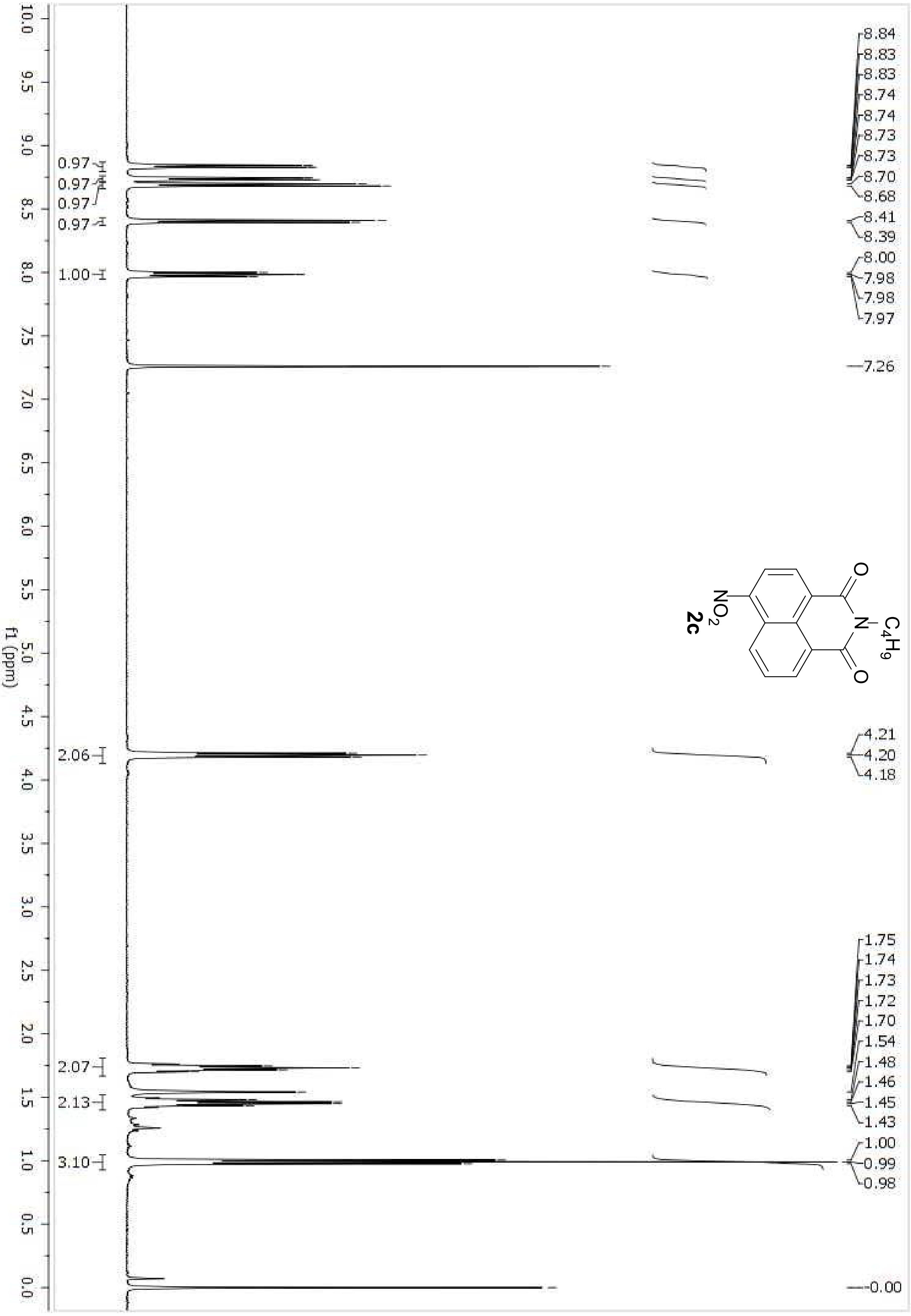

Figure S20. ^{1}H NMR spectrum of **2c** (500 MHz, $CDCl_3$).

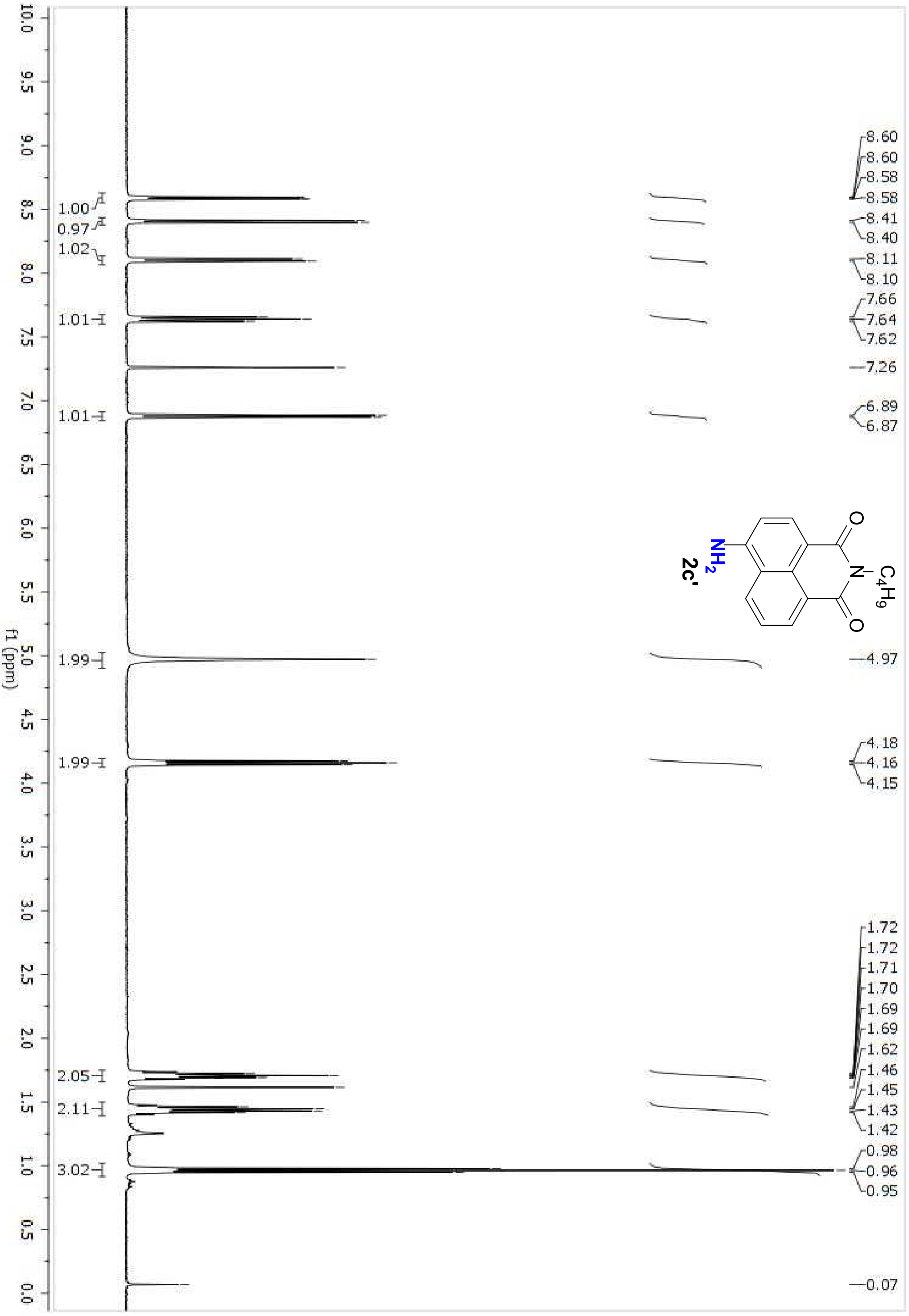

Figure S21. ^{1}H NMR spectrum of **2c'** (500 MHz, $CDCl_3$).

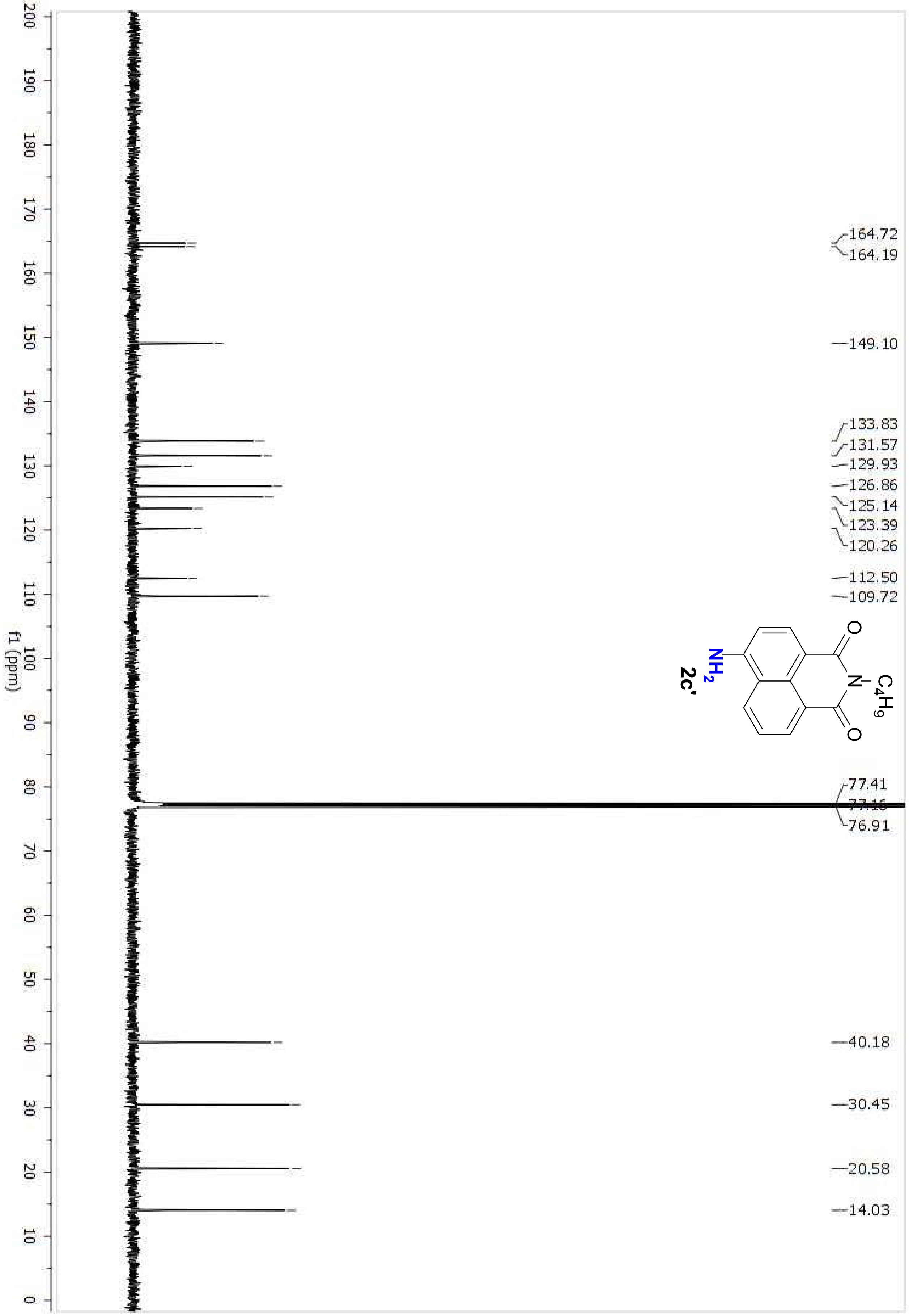

Figure S22. ^{13}C NMR spectrum of **2c’** (126 MHz, $CDCl_3$).

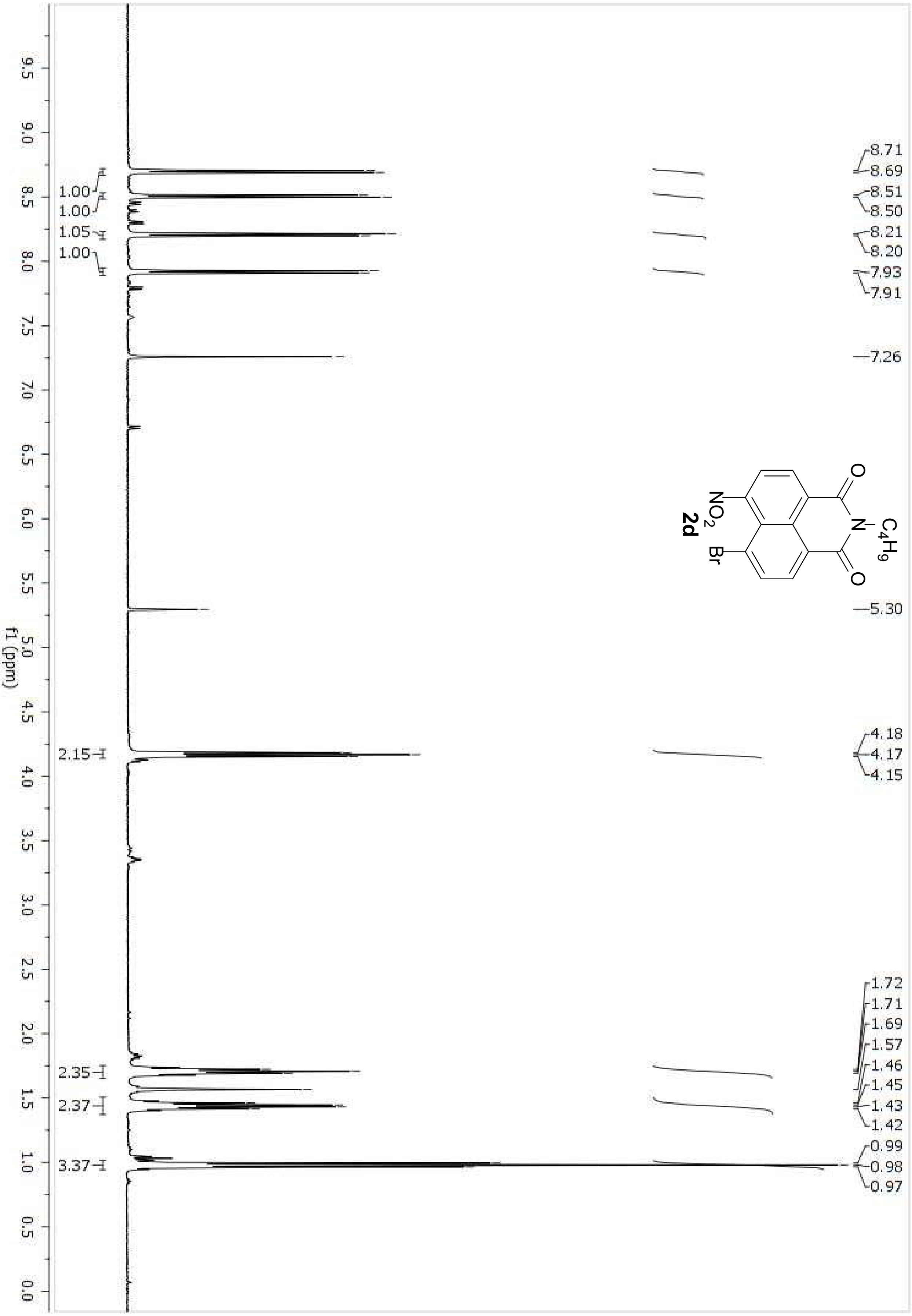

Figure S23. ^{1}H NMR spectrum of **2d** (500 MHz, $CDCl_3$).

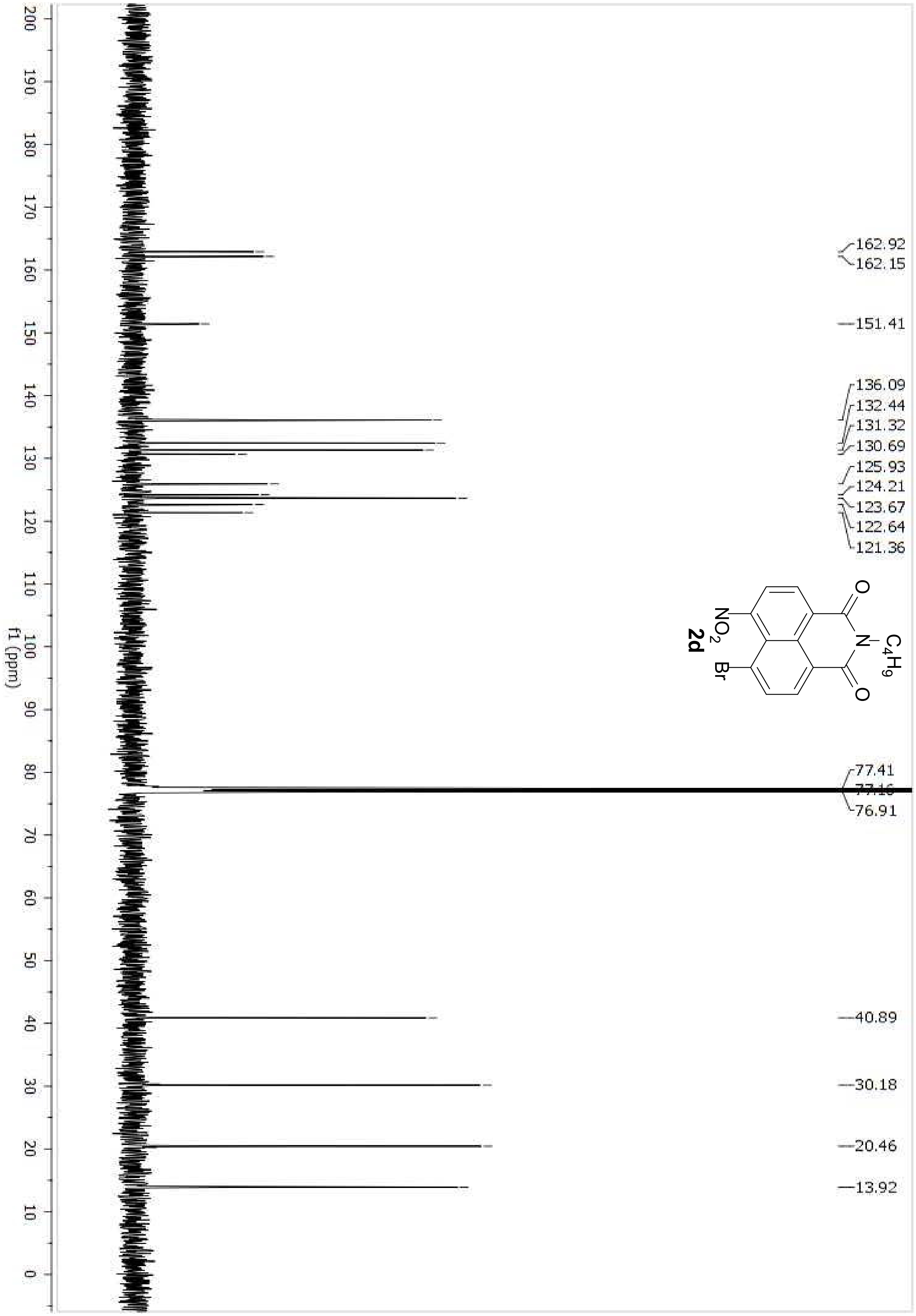

Figure S24. ^{13}C NMR spectrum of **2d** (126 MHz, $CDCl_3$).

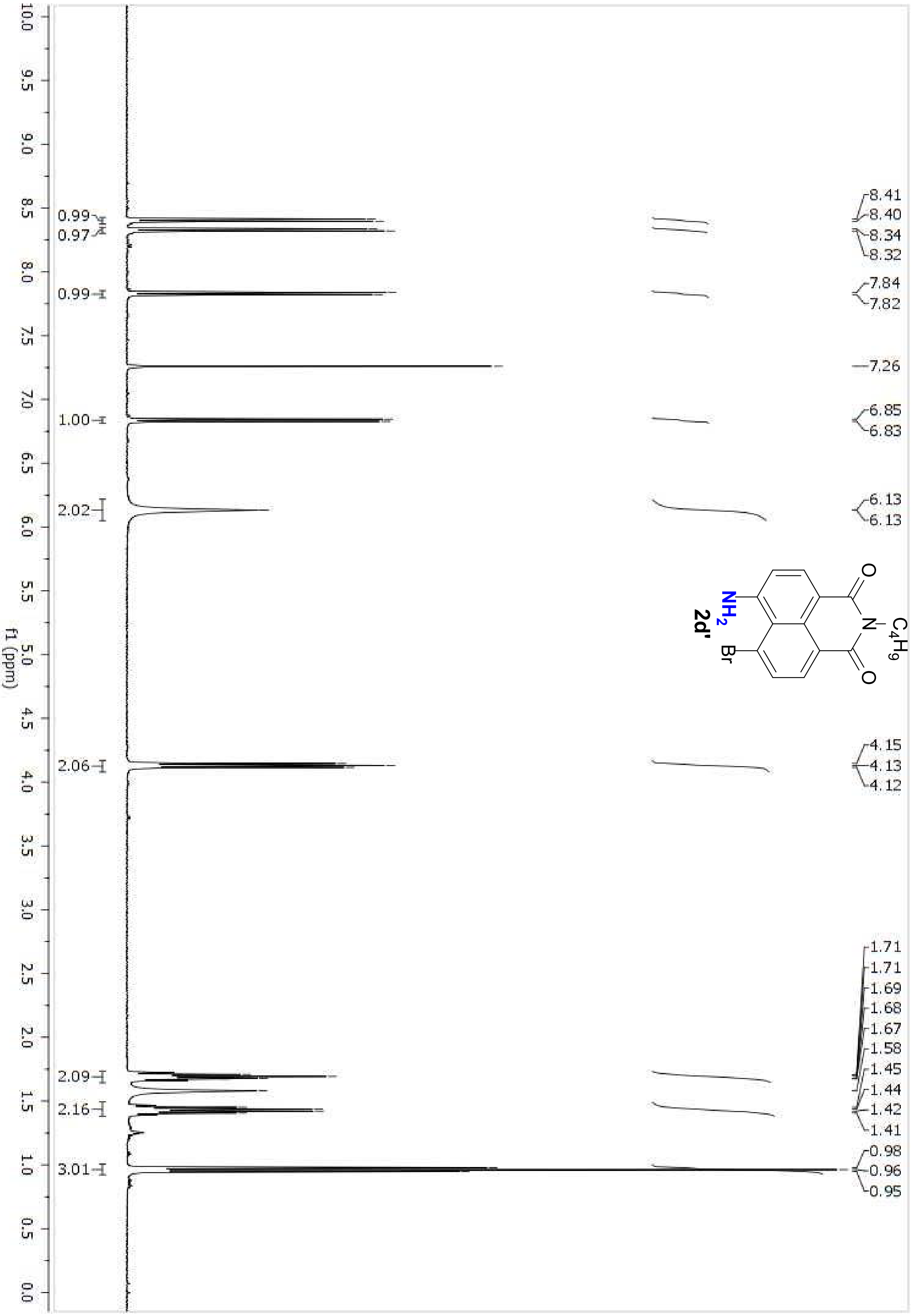

Figure S25. ^{1}H NMR spectrum of **2d'** (500 MHz, $CDCl_3$).

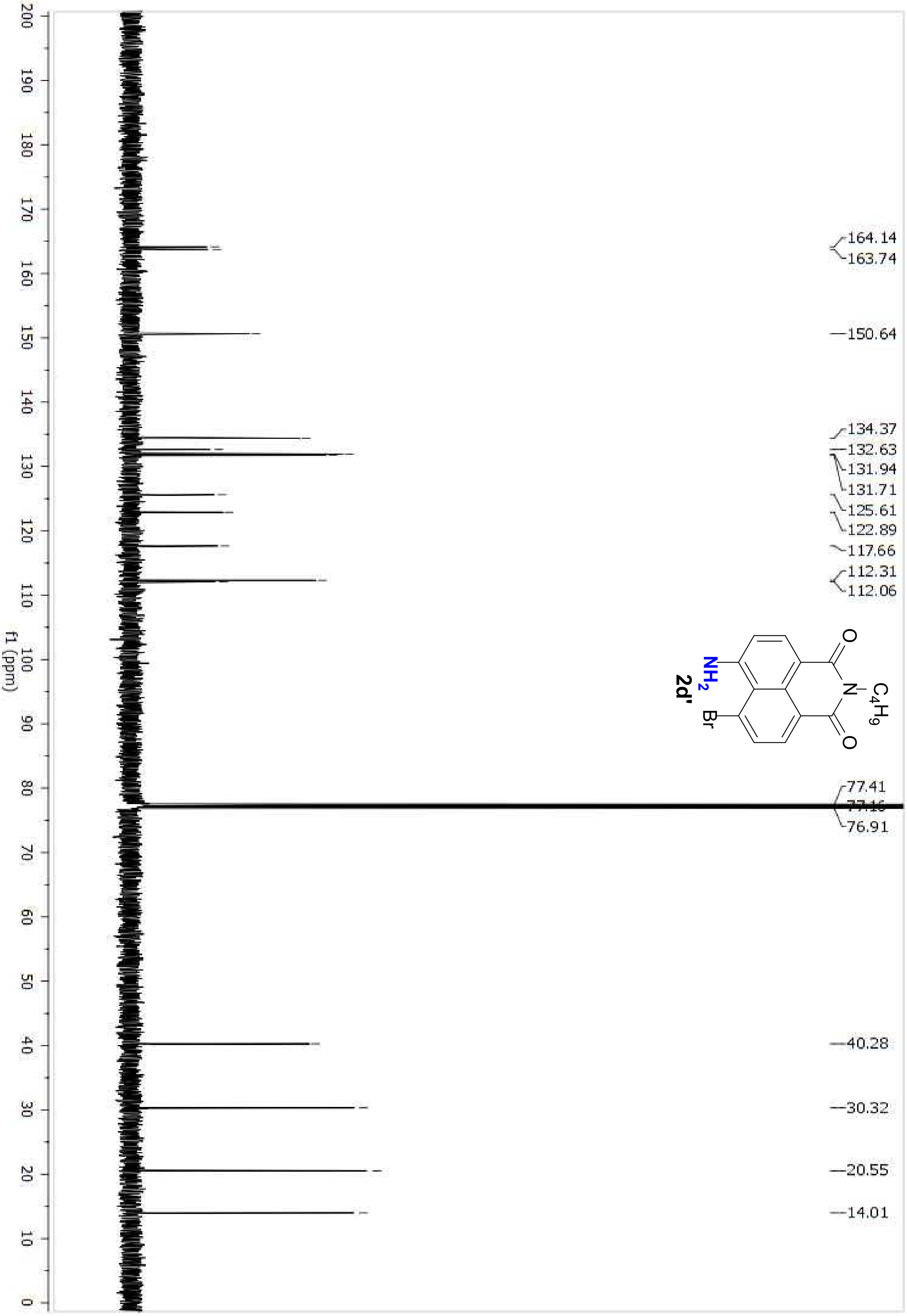

Figure S26. ^{13}C NMR spectrum of **2d’** (126 MHz, $CDCl_3$).

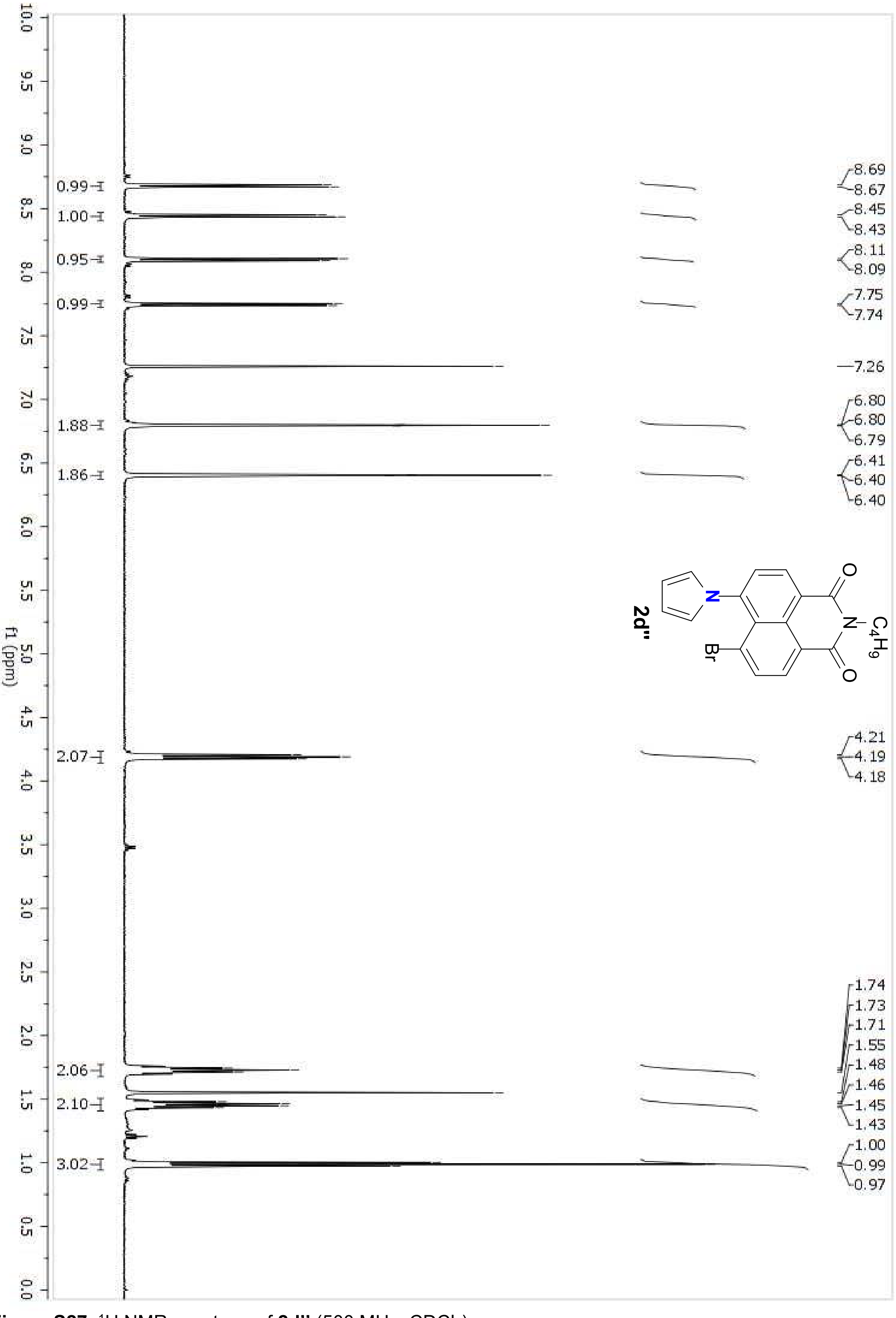

Figure S27. ^1H NMR spectrum of **2d''** (500 MHz, $CDCl_3$).

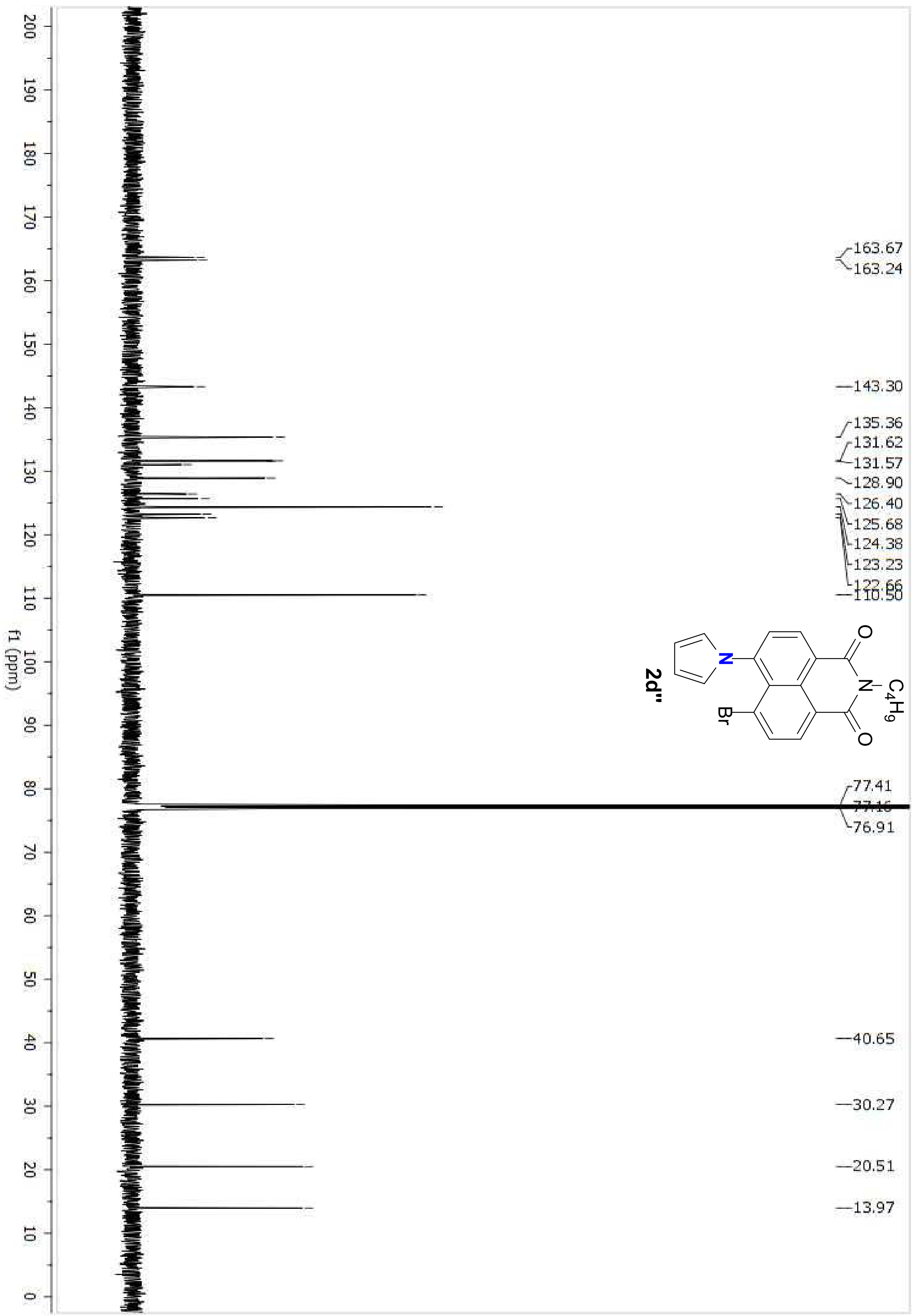

Figure S28. ^{13}C NMR spectrum of **2d''** (126 MHz, $CDCl_3$).

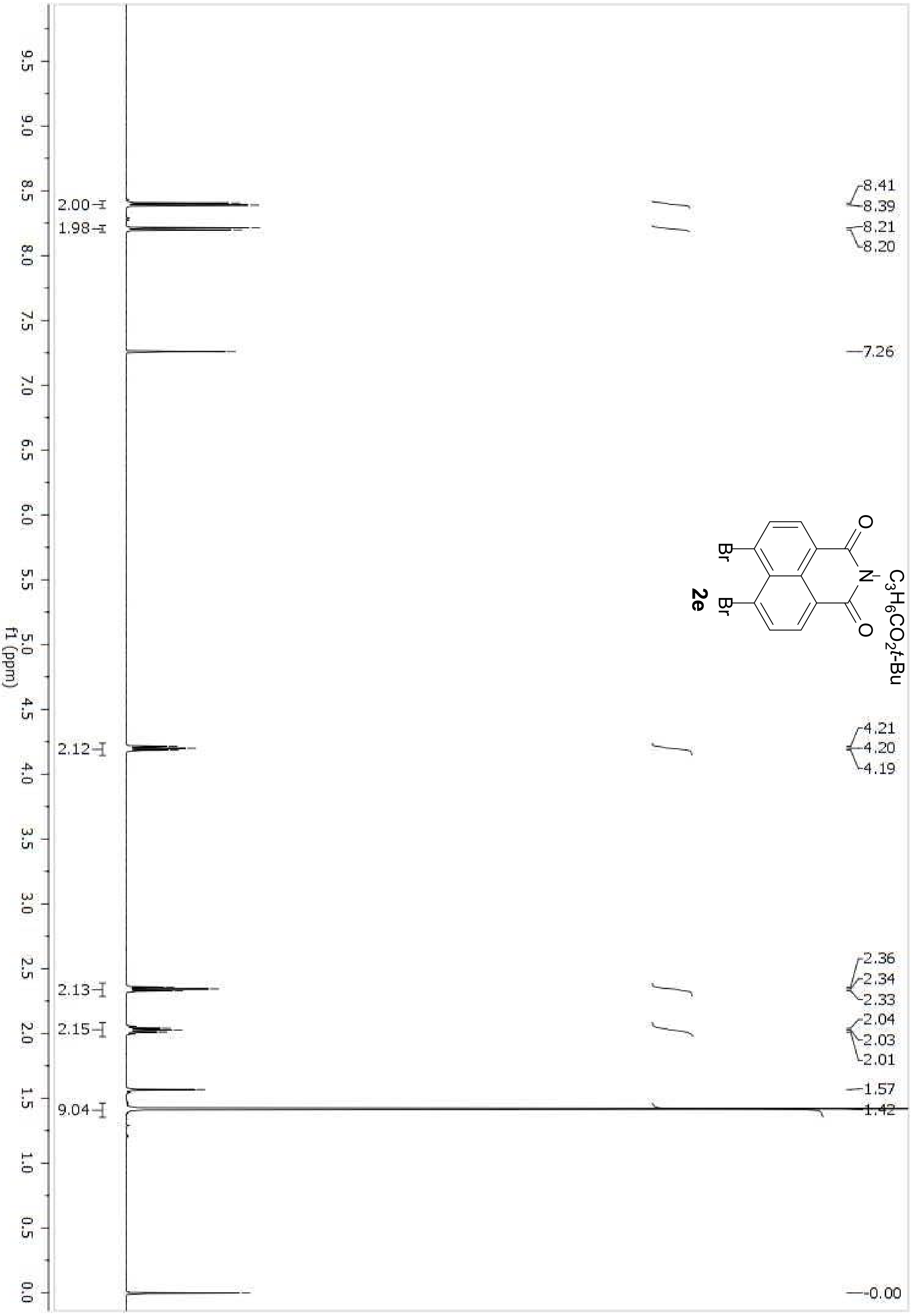

Figure S29. ^{1}H NMR spectrum of **2e** (500 MHz, $CDCl_3$).

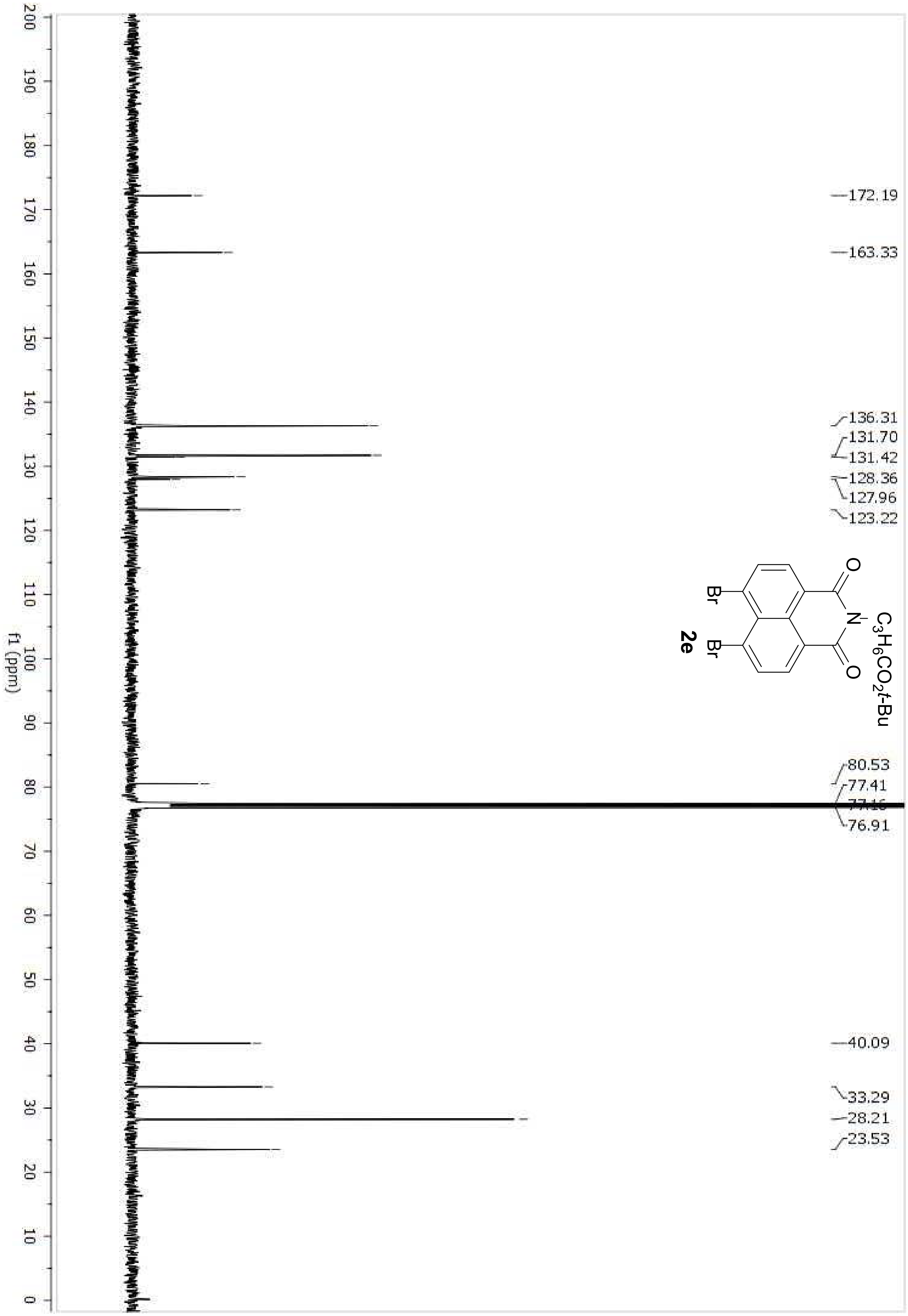

Figure S30. ^{13}C NMR spectrum of **2e** (126 MHz, $CDCl_3$).

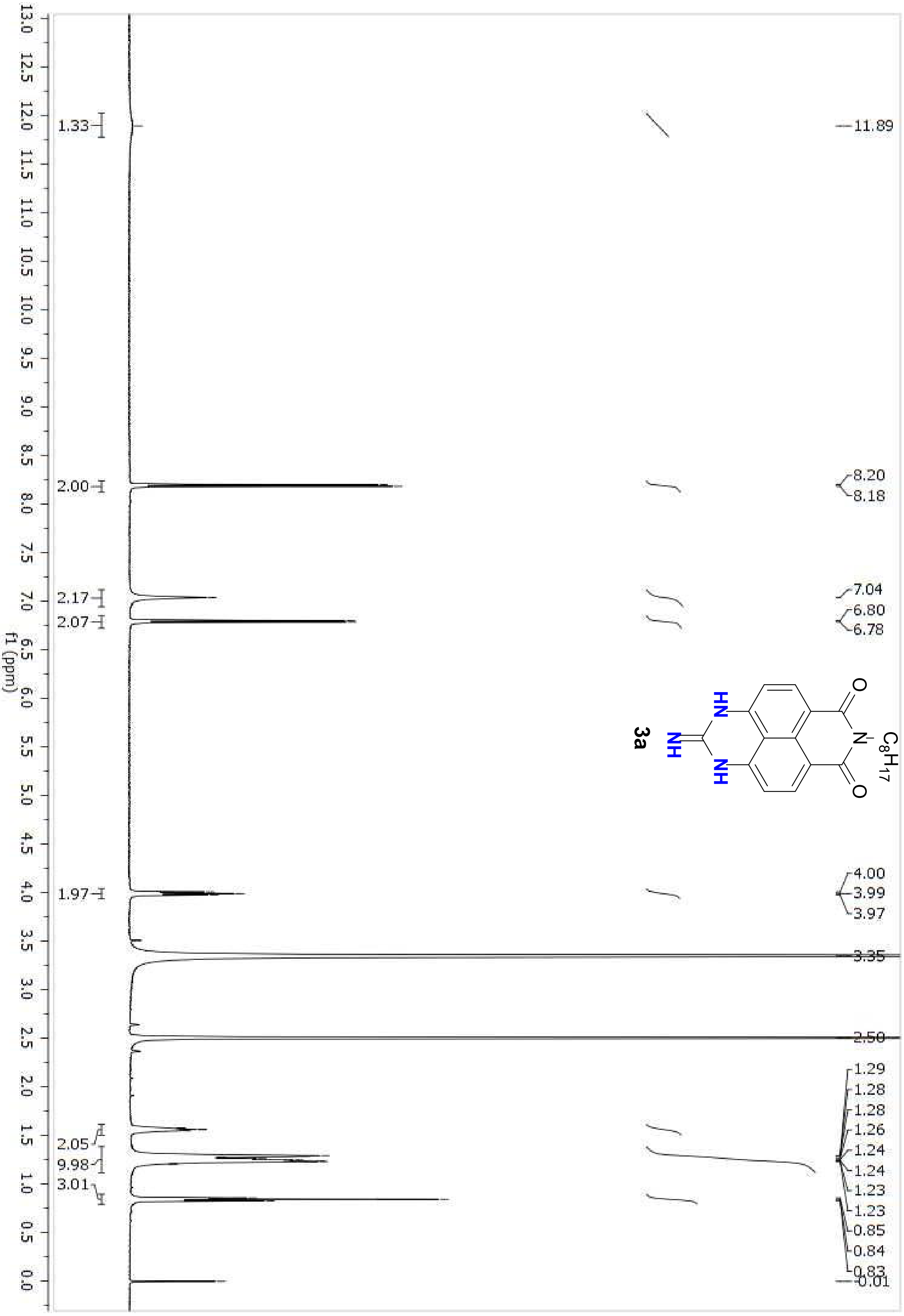

Figure S31. ^{1}H NMR spectrum of **3a** (500 MHz, [D$_6$]-DMSO).

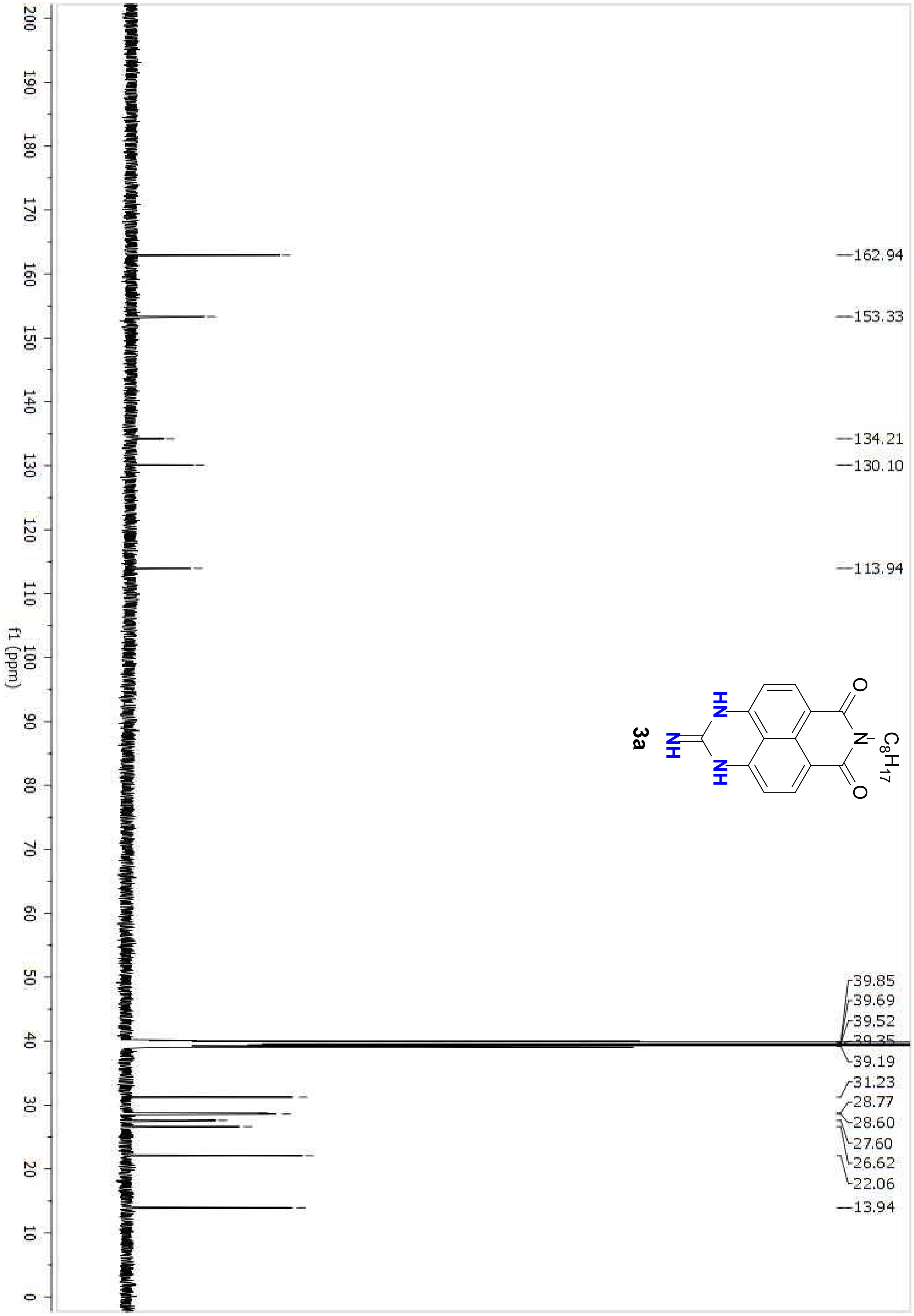

Figure S32. ^{13}C NMR spectrum of **3a** (126 MHz, [D$_6$]-DMSO).

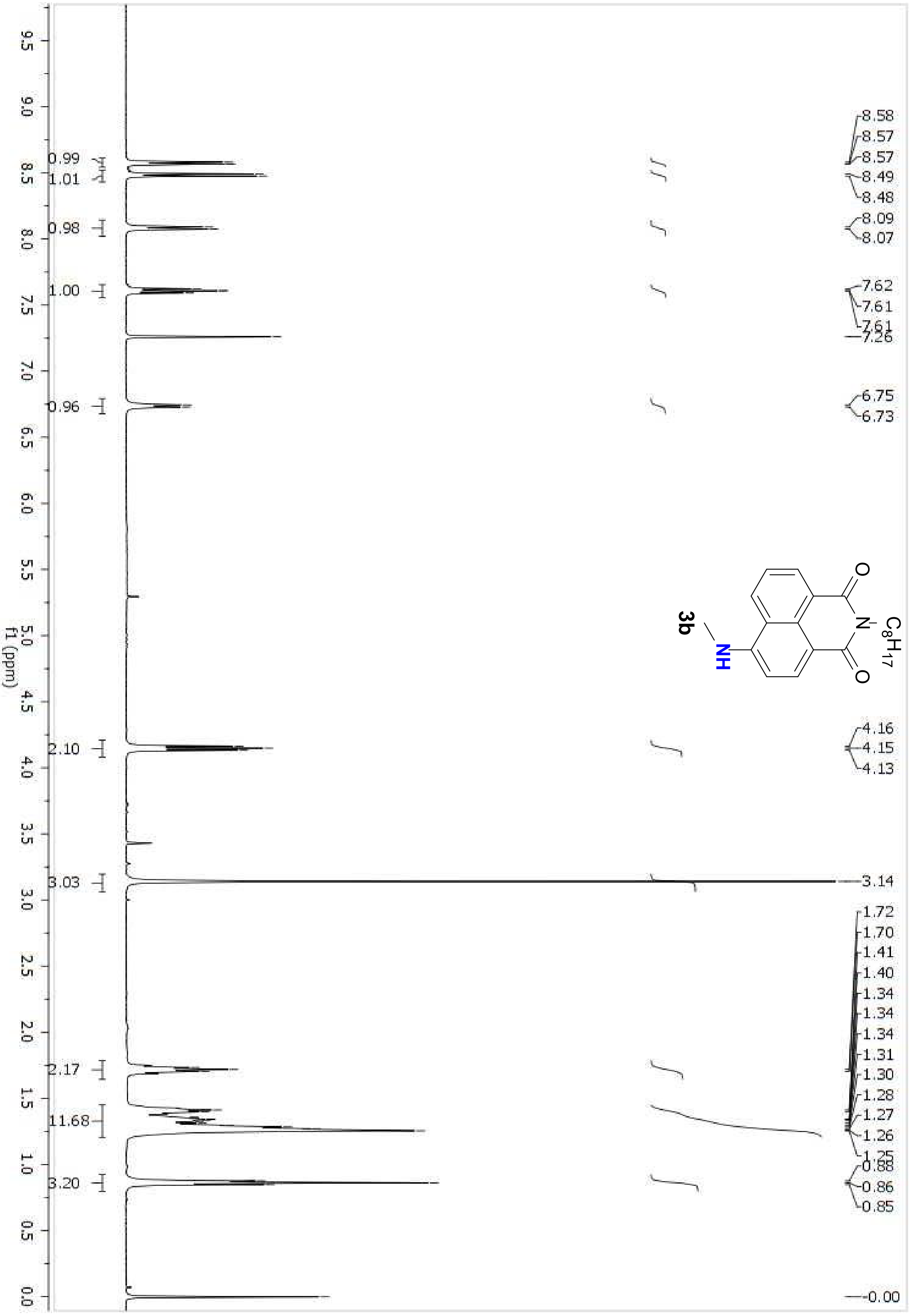

Figure S33. ^{1}H NMR spectrum of **3b** (500 MHz, $CDCl_3$).

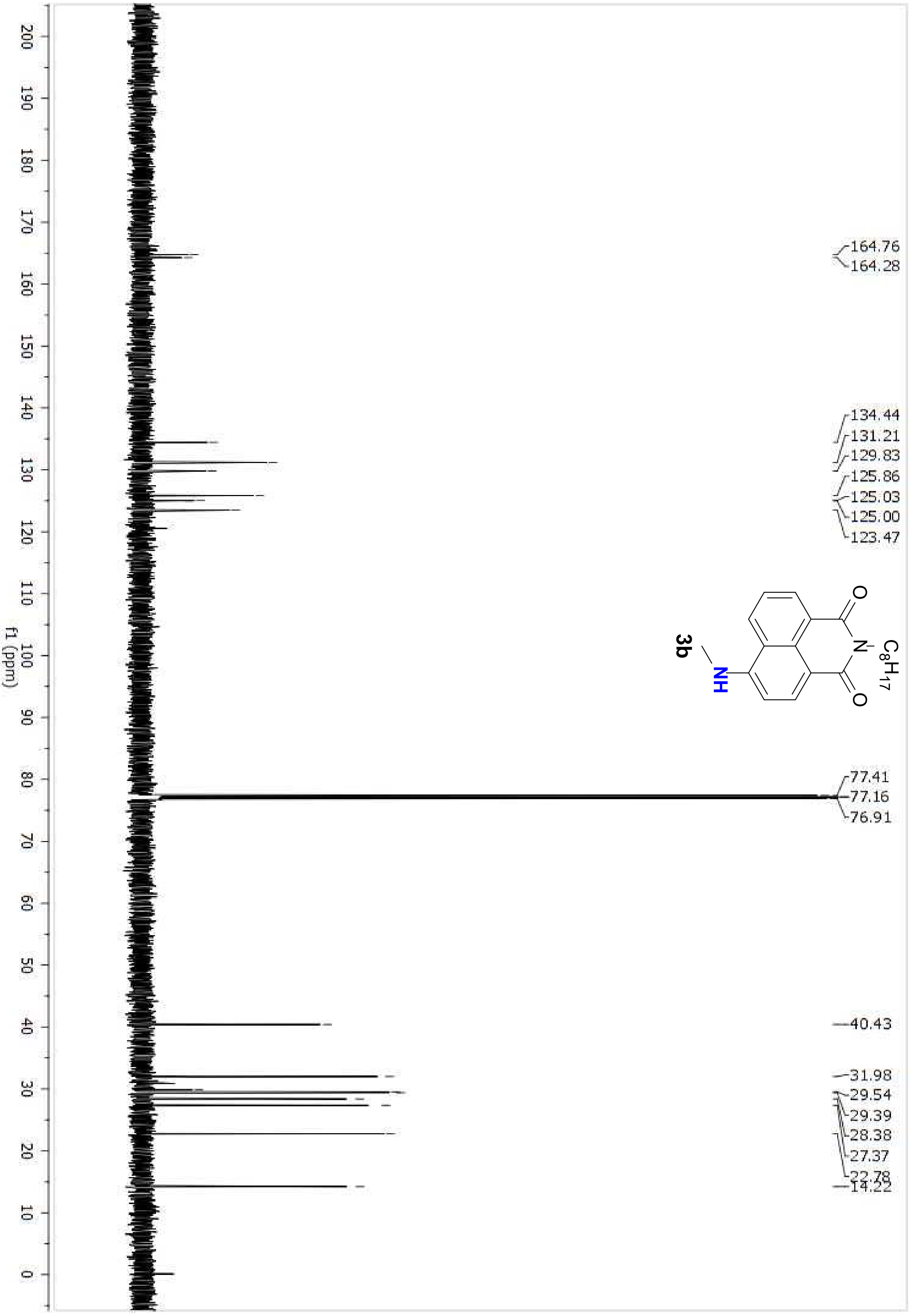

Figure S34. ^{13}C NMR spectrum of **3b** (126 MHz, $CDCl_3$).

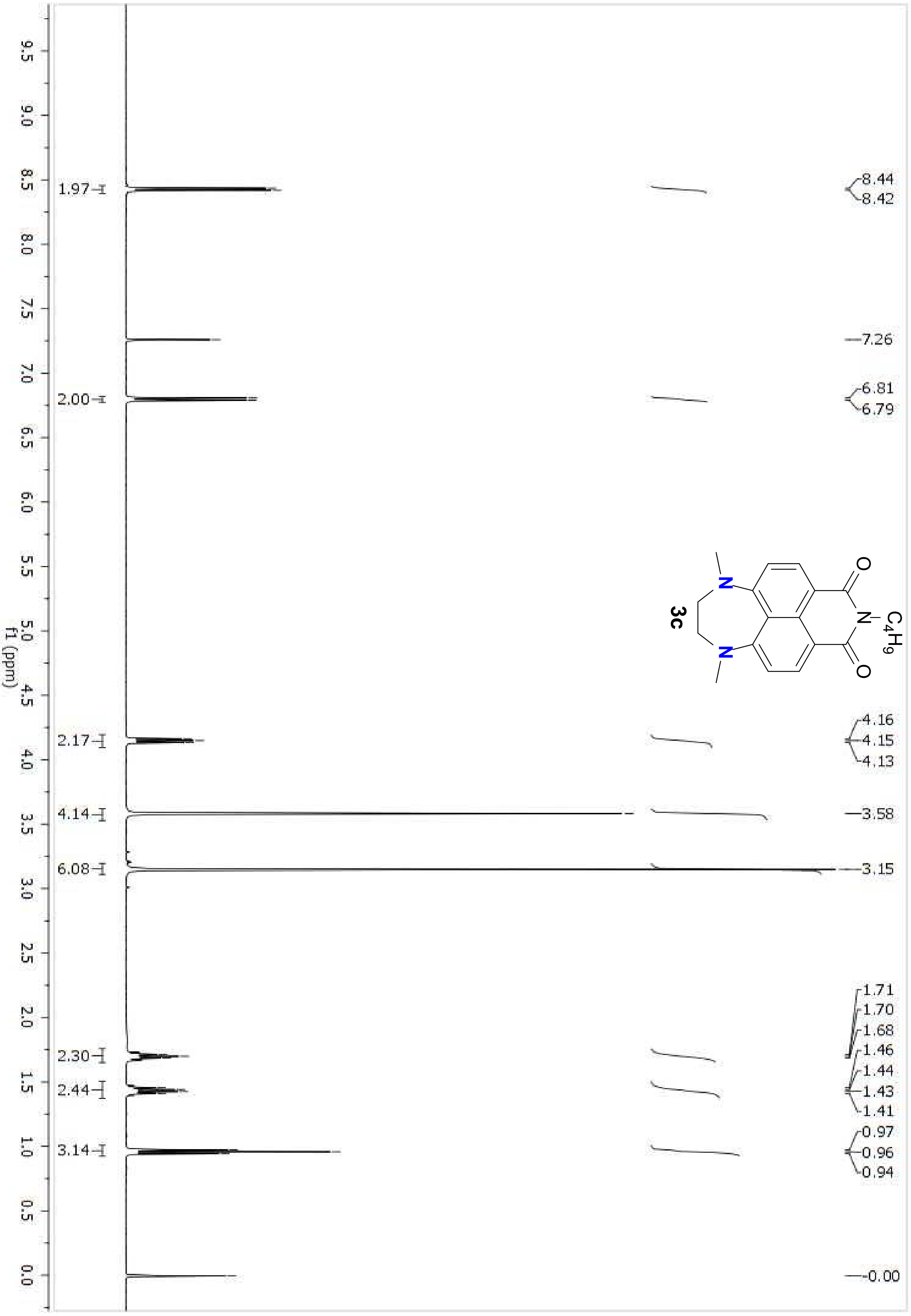

Figure S35. ^{1}H NMR spectrum of **3c** (500 MHz, $CDCl_3$).

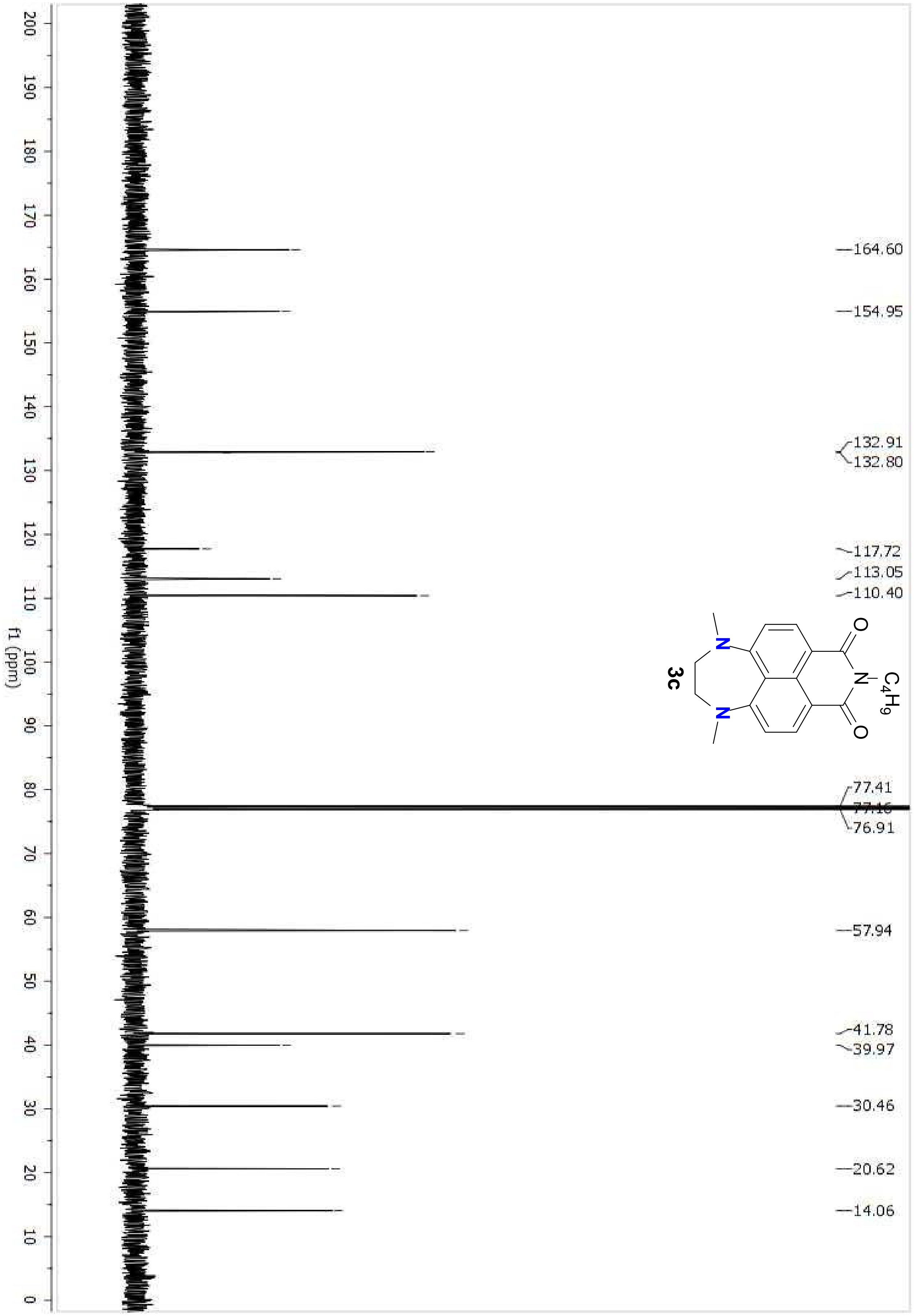

Figure S36. ^{13}C NMR spectrum of **3c** (126 MHz, $CDCl_3$).

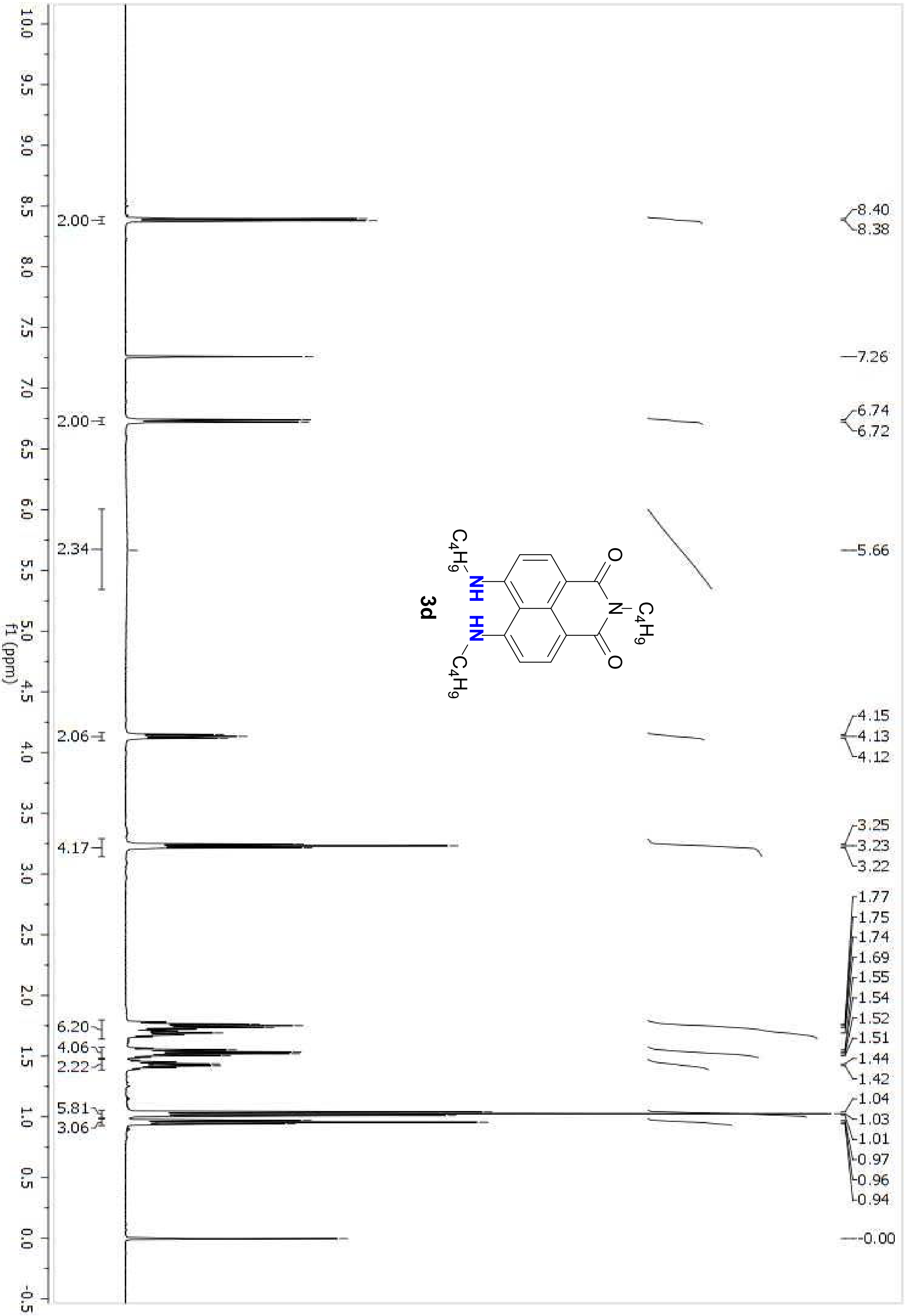

Figure S37. ^{1}H NMR spectrum of **3d** (500 MHz, $CDCl_3$).

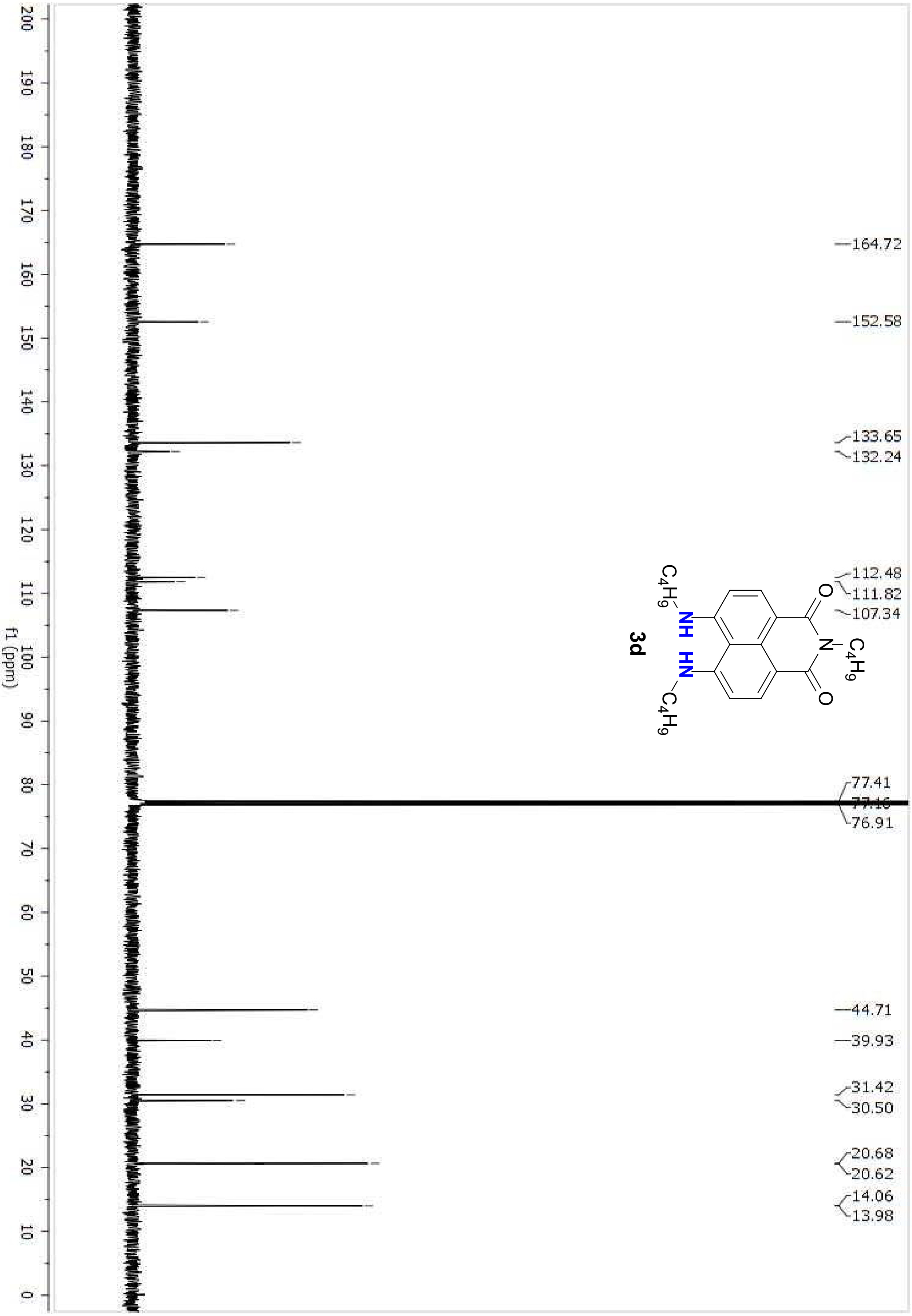

Figure S38. ^{13}C NMR spectrum of **3d** (126 MHz, $CDCl_3$).

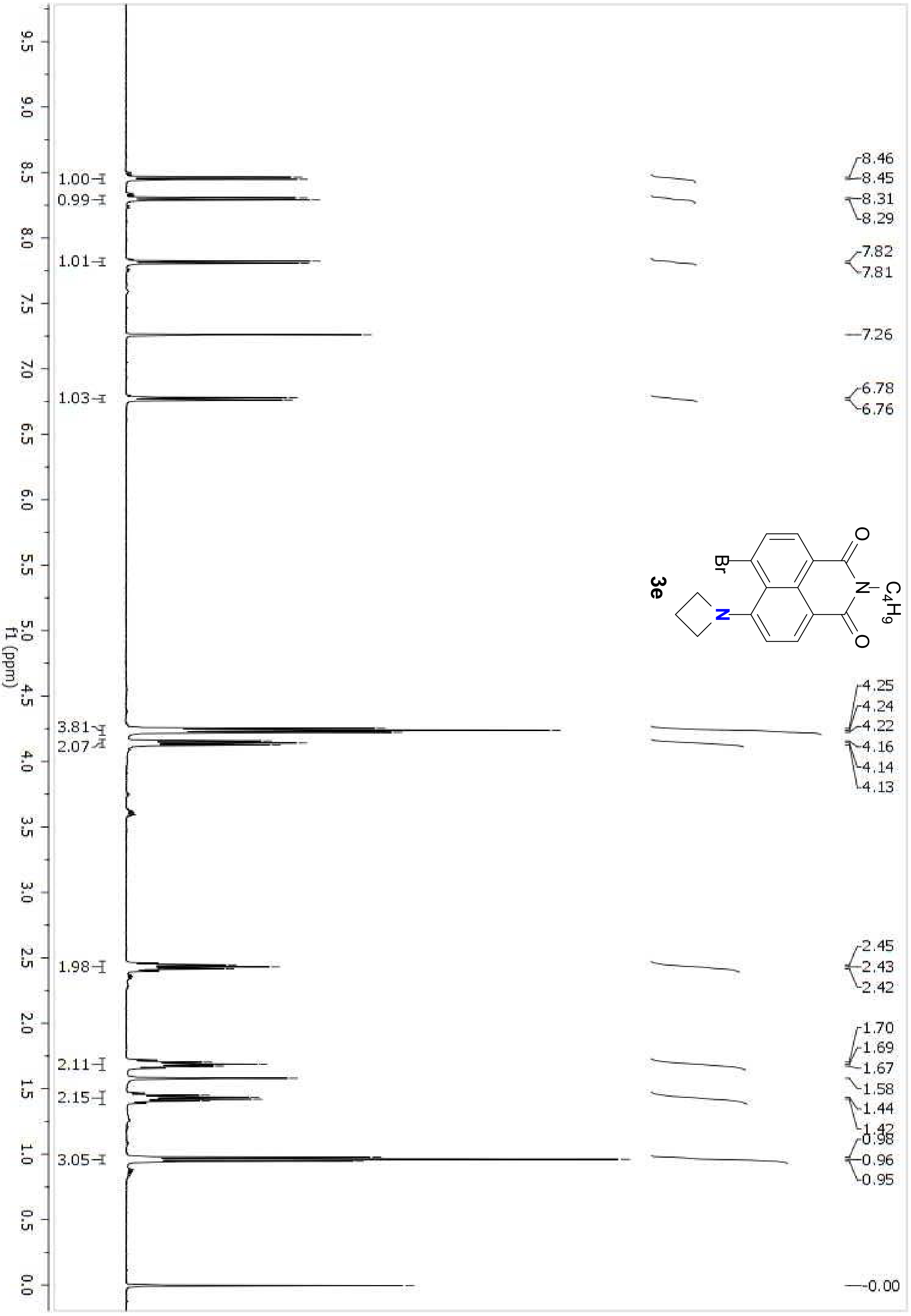

Figure S39. ^{1}H NMR spectrum of **3e** (500 MHz, $CDCl_3$).

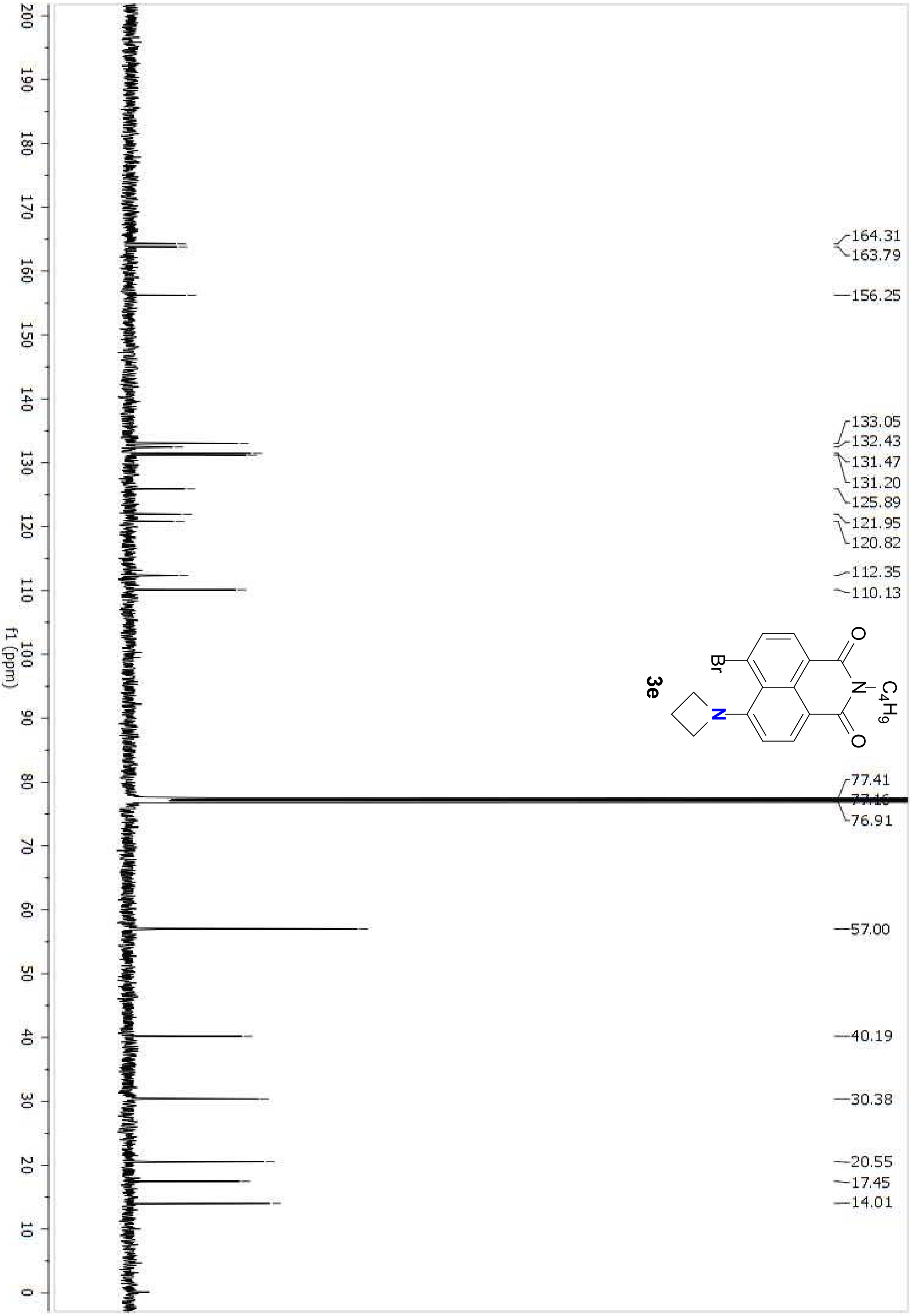

Figure S40. ^{13}C NMR spectrum of **3e** (126 MHz, $CDCl_3$).

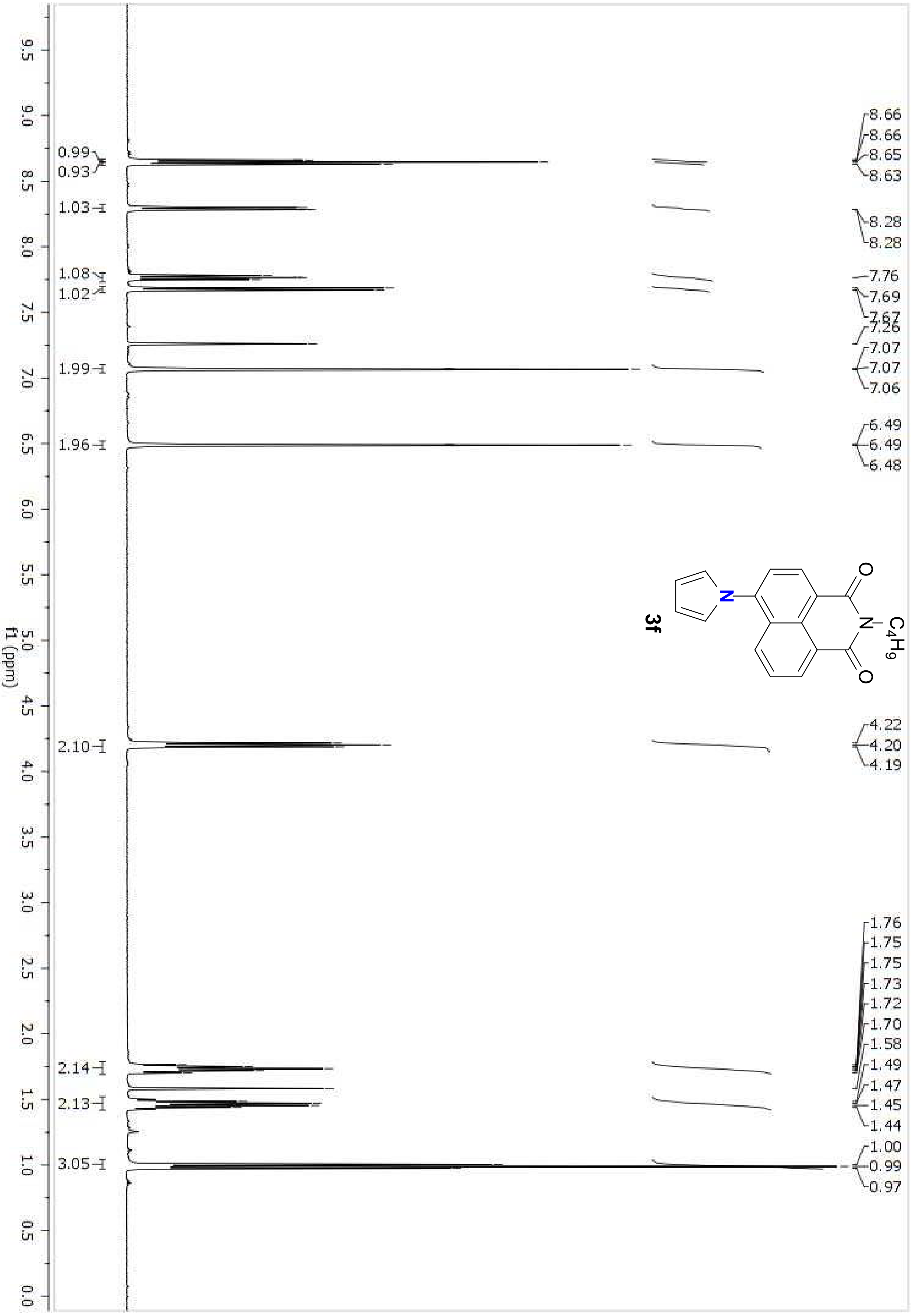

Figure S41. ^{1}H NMR spectrum of **3f** (500 MHz, $CDCl_3$).

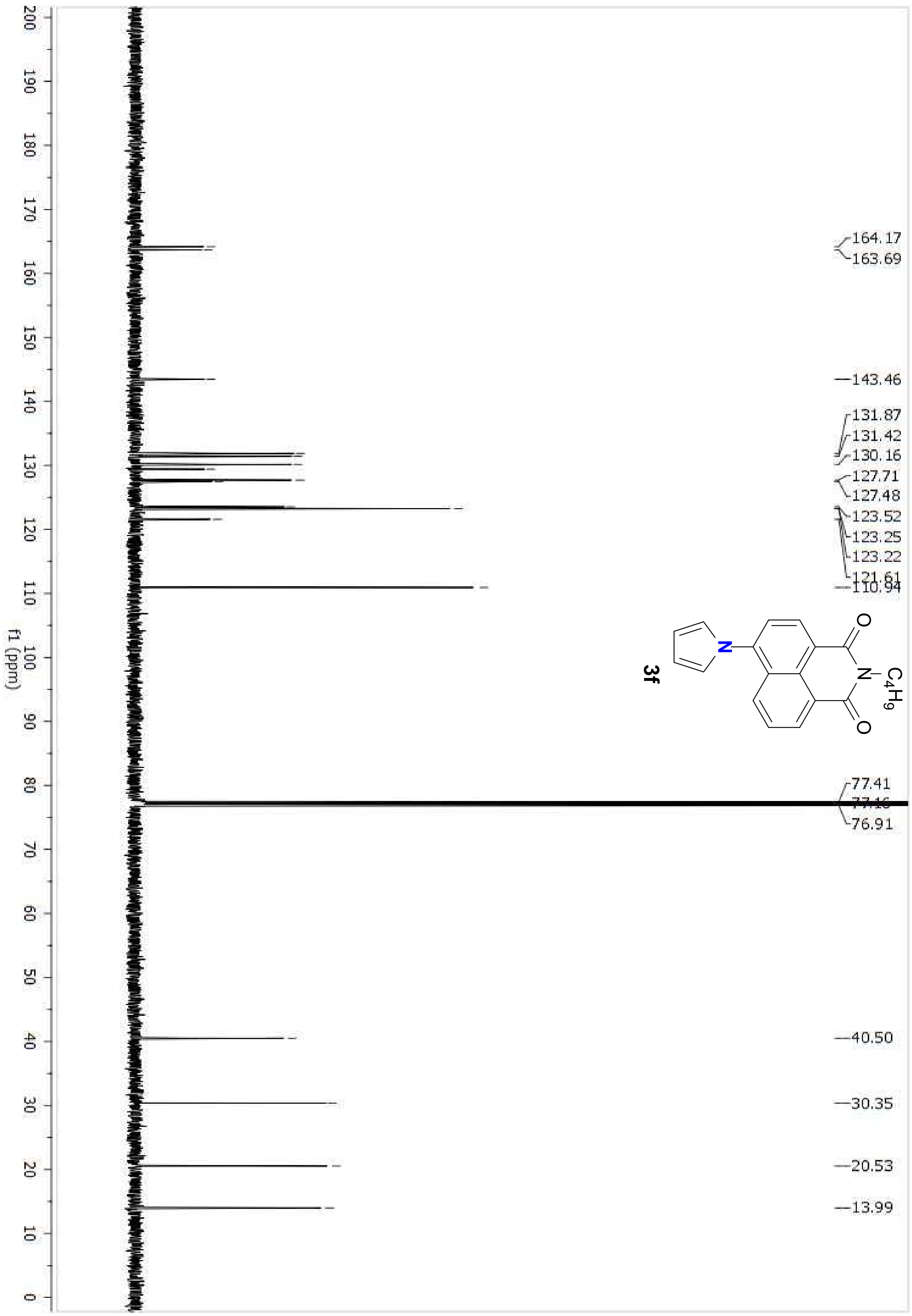

Figure S42. ^{13}C NMR spectrum of **3f** (126 MHz, $CDCl_3$).

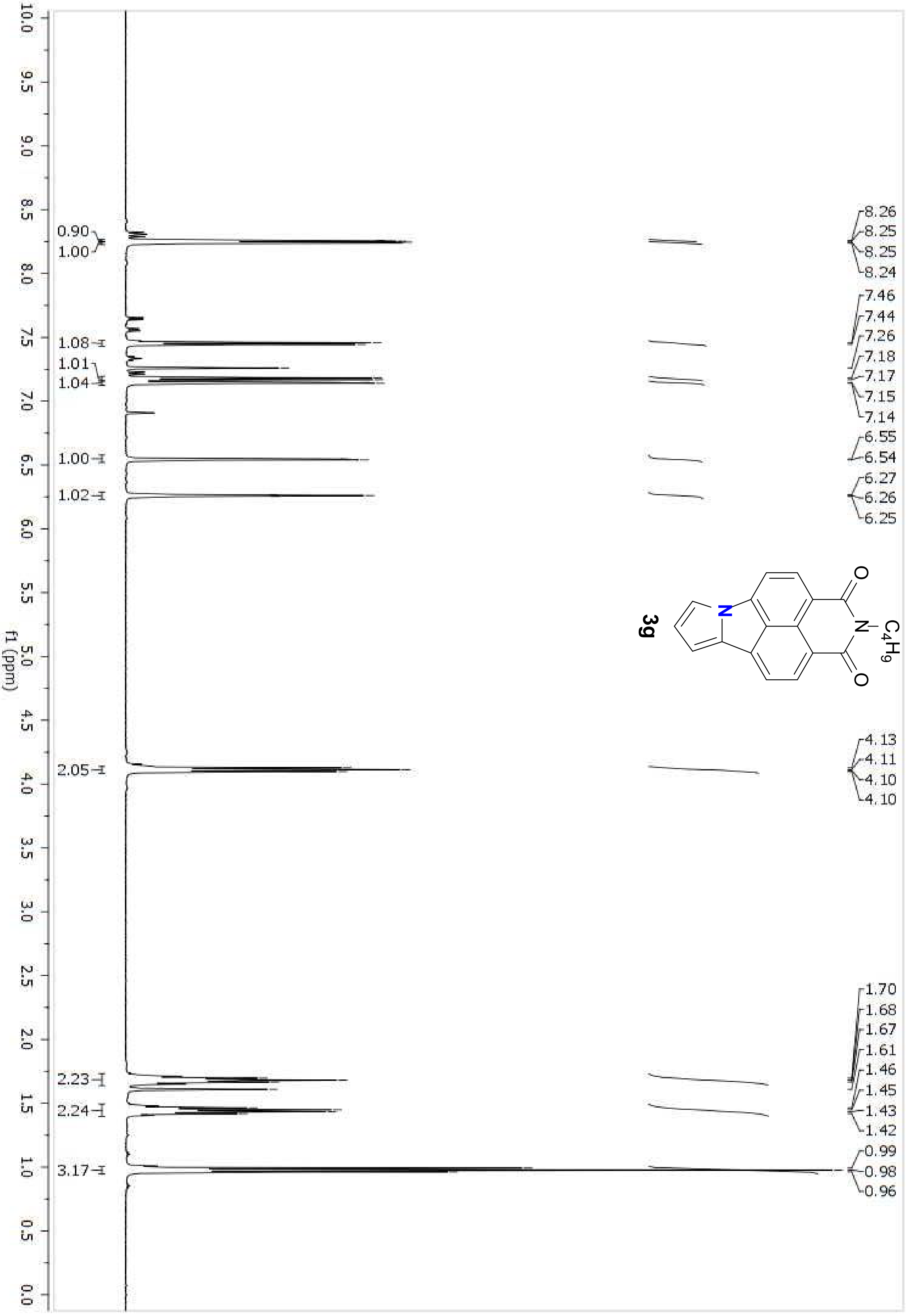

Figure S43. ^{1}H NMR spectrum of **3g** (500 MHz, $CDCl_3$).

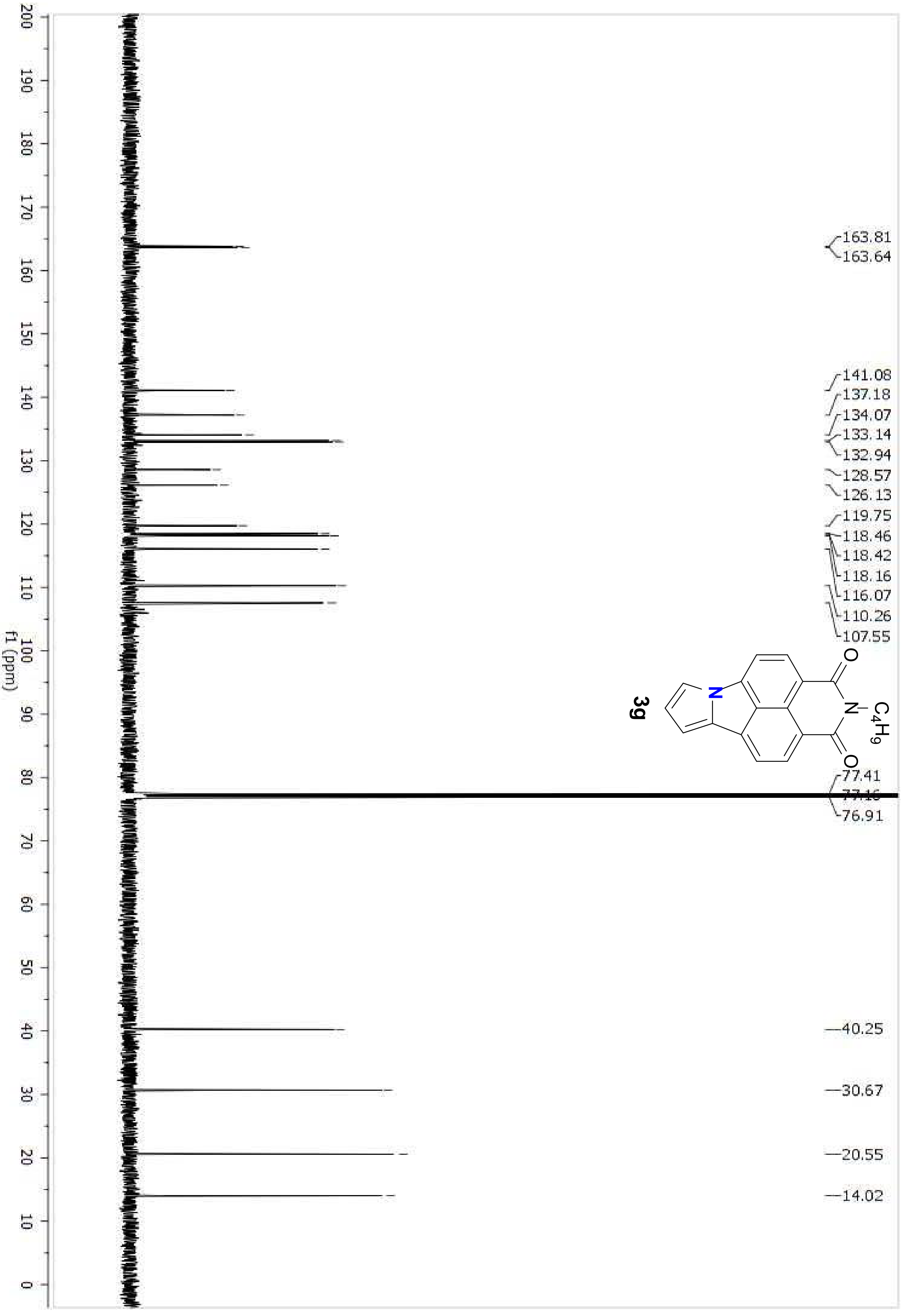

Figure S44. ^{13}C NMR spectrum of **3g** (126 MHz, $CDCl_3$).

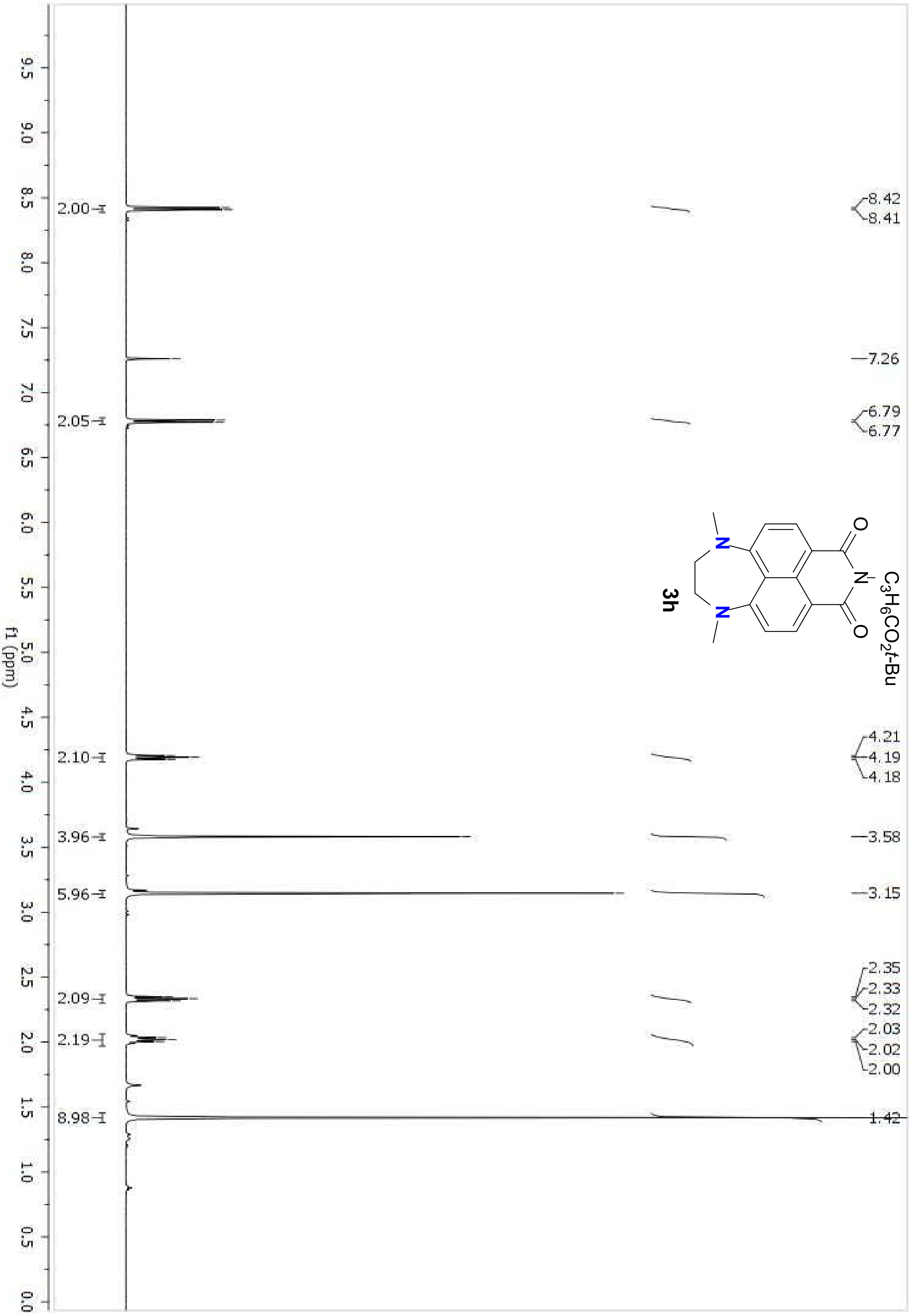

Figure S45. ^{1}H NMR spectrum of **3h** (500 MHz, $CDCl_3$).

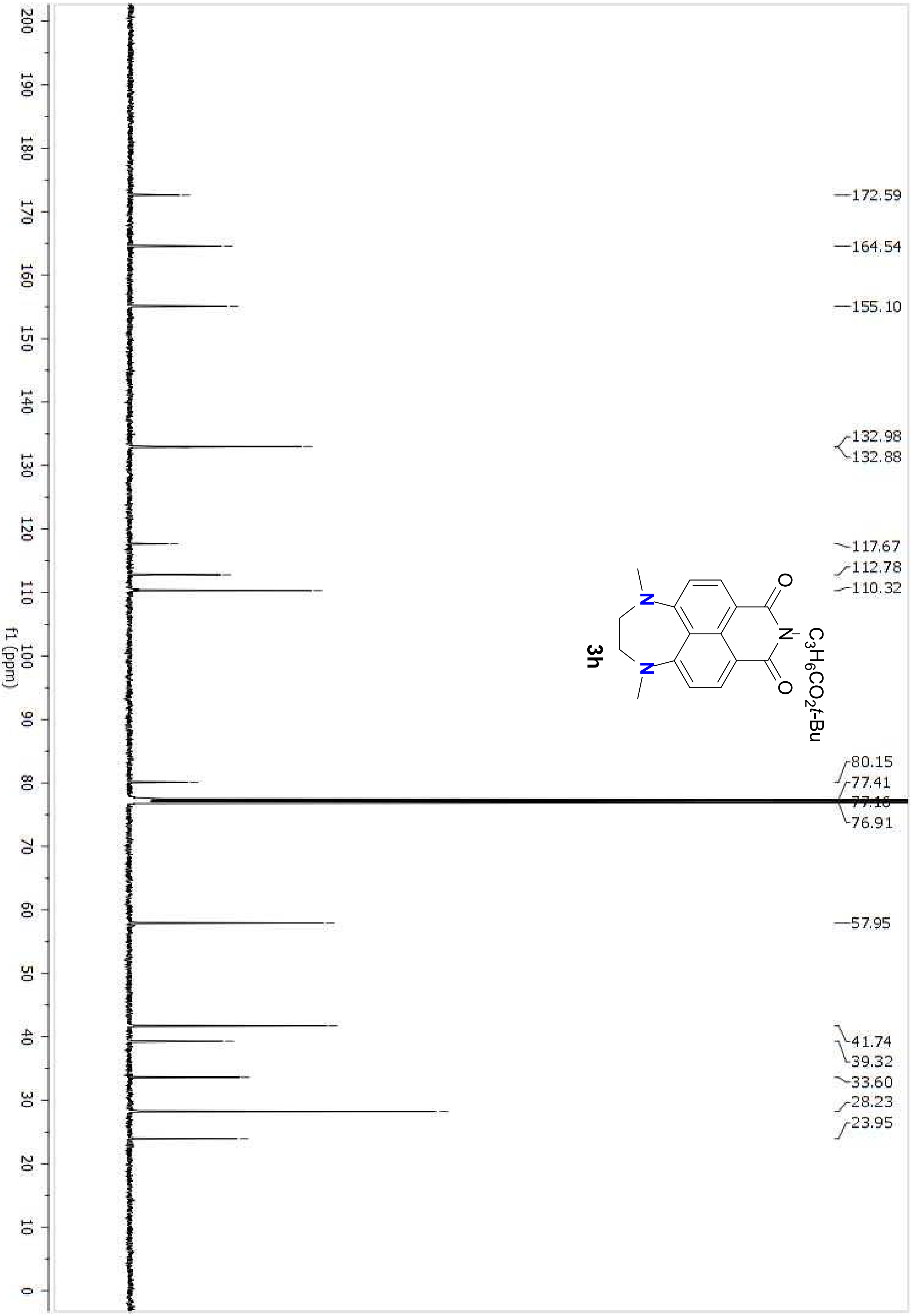

Figure S46. ^{13}C NMR spectrum of **3h** (126 MHz, $CDCl_3$).

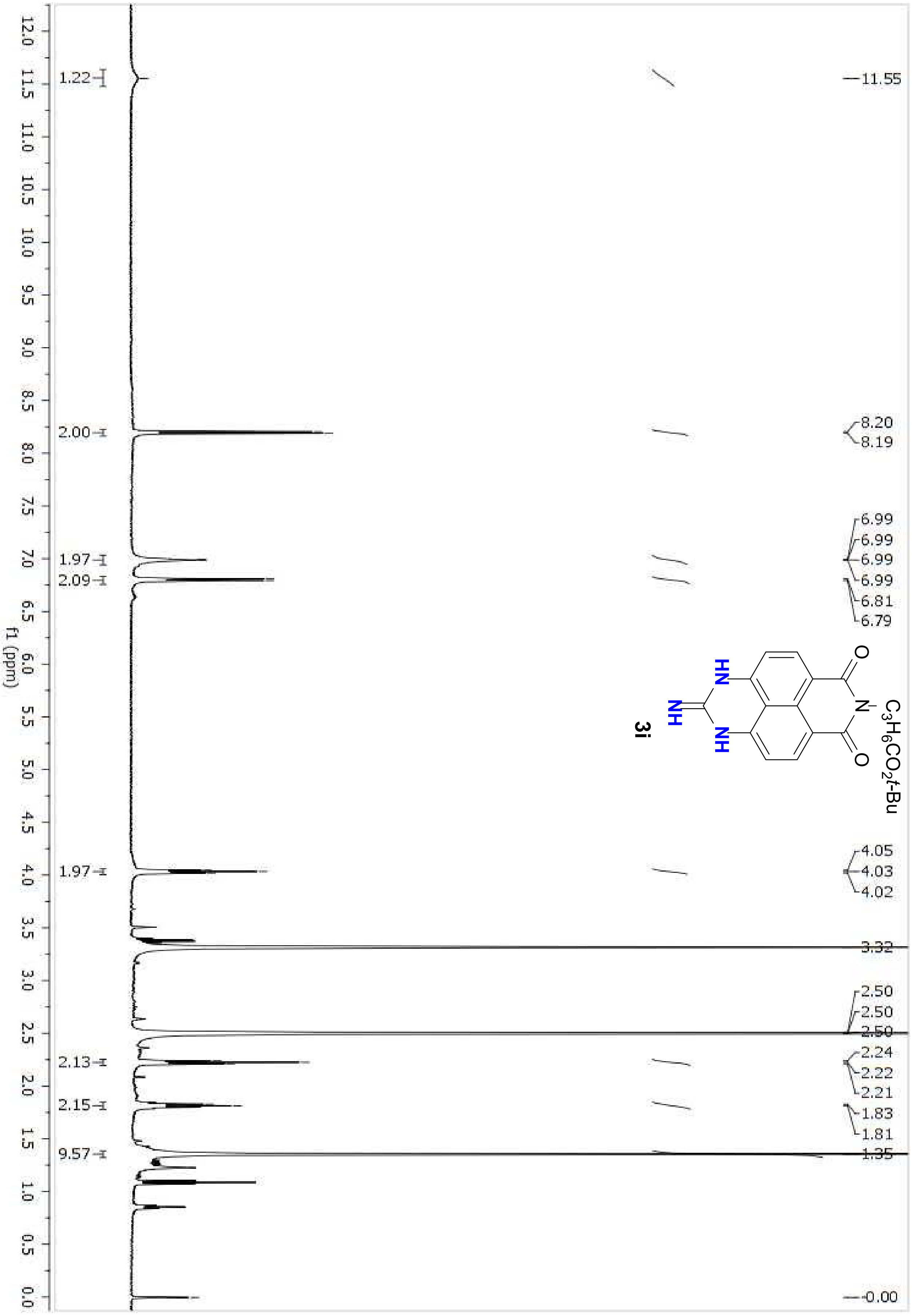

Figure S47. ^{1}H NMR spectrum of **3i** (500 MHz, [D6]-DMSO).

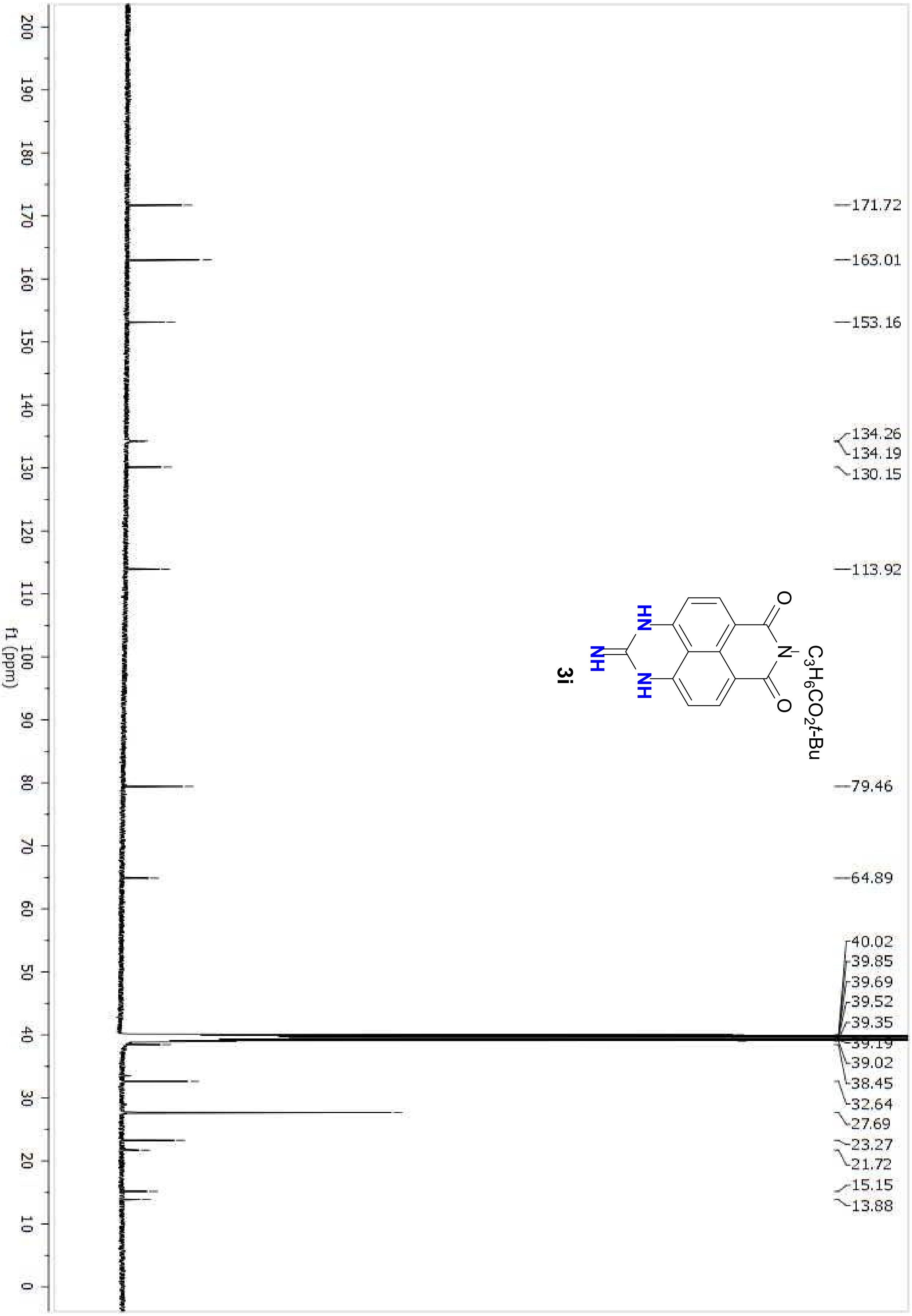

Figure S48. ^{13}C NMR spectrum of **3i** (126 MHz, [D6]-DMSO).

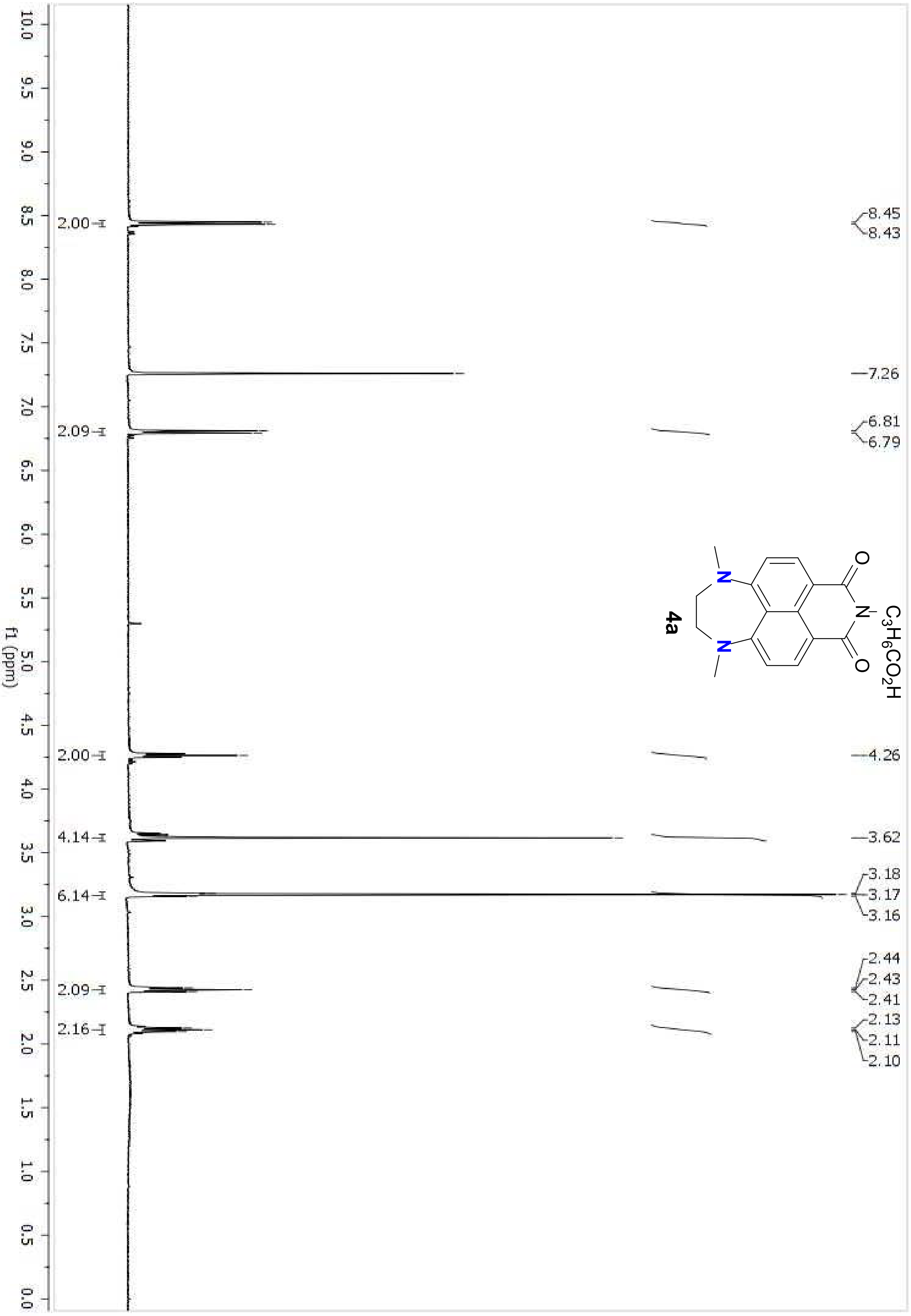

Figure S49. ^{1}H NMR spectrum of **4a** (500 MHz, $CDCl_3$).

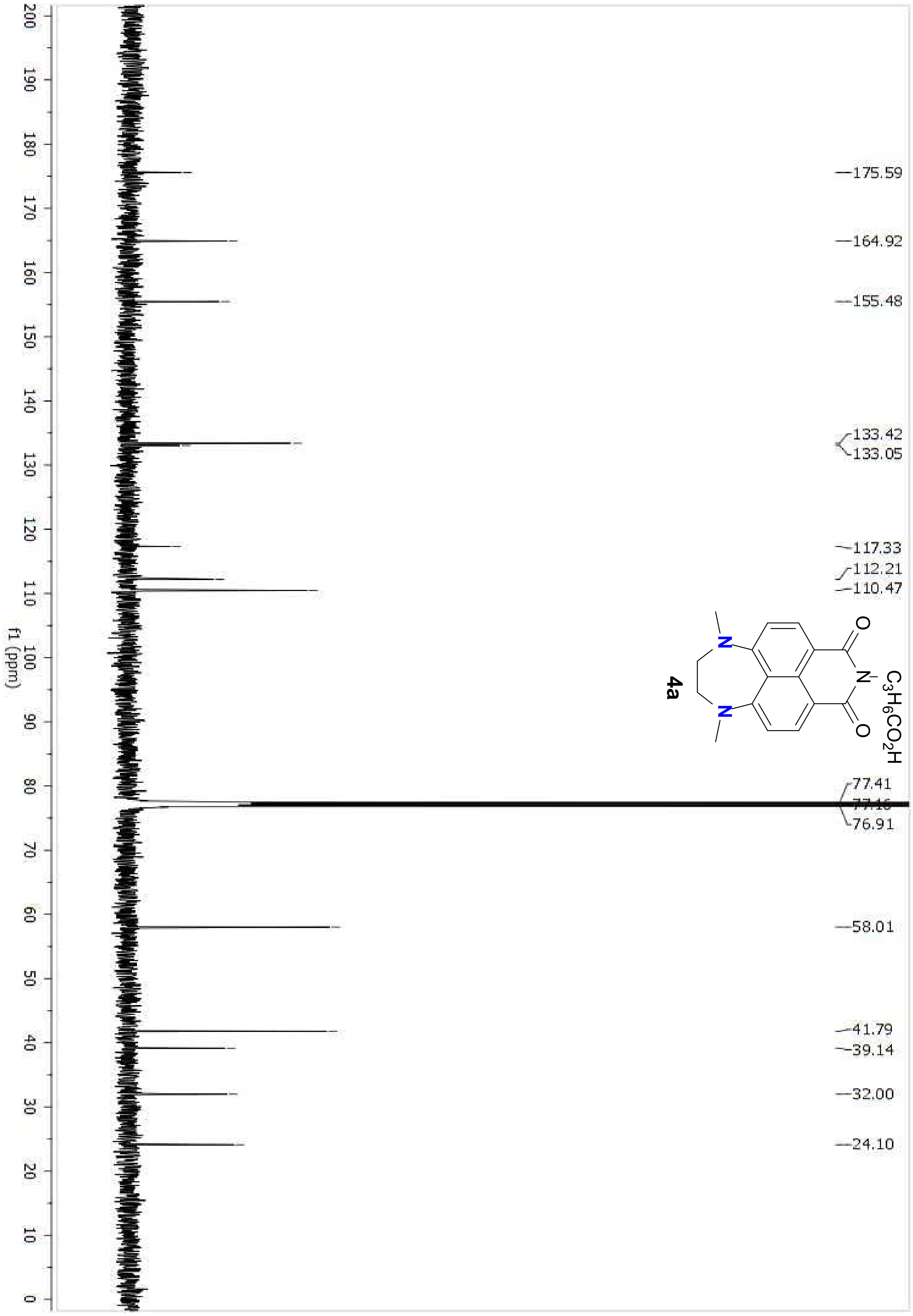

Figure S50. ^{13}C NMR spectrum of **4a** (126 MHz, $CDCl_3$).

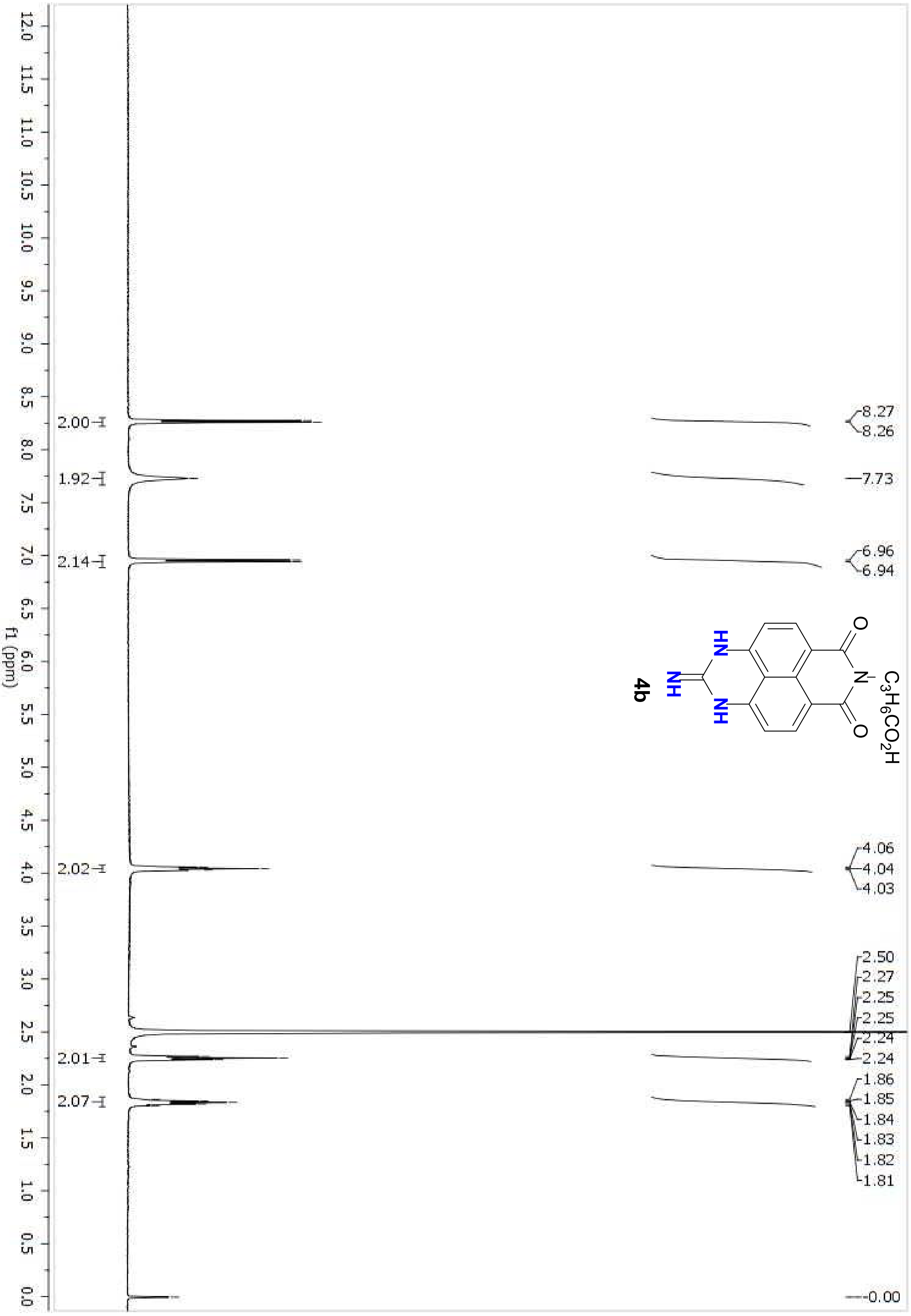

Figure S51. ^{1}H NMR spectrum of **4b** (500 MHz, [D6]-DMSO).

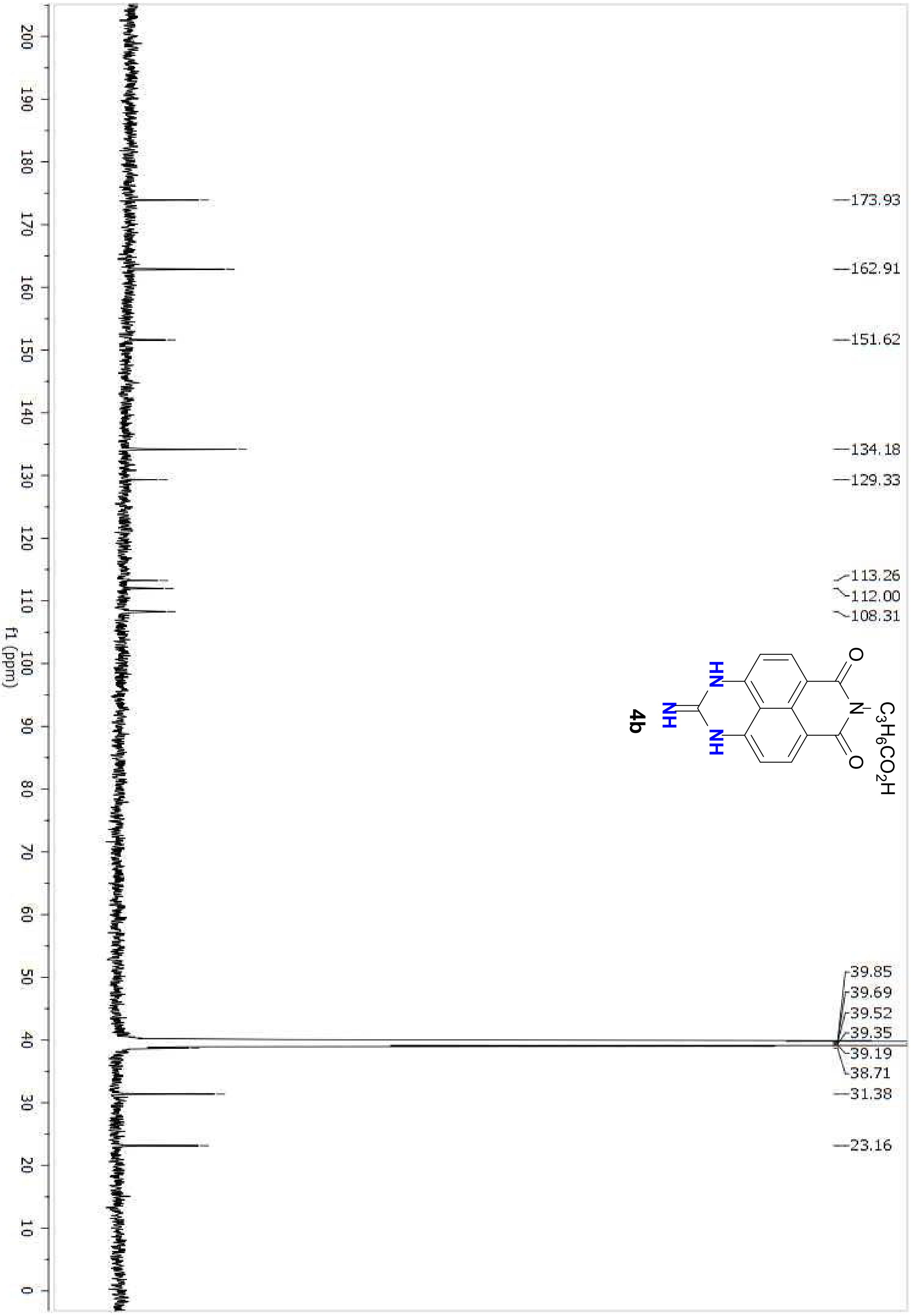

Figure S52. ^{13}C NMR spectrum of **4b** (126 MHz, [D_6]-DMSO).

7. References

(1) Uersfeld, D.; Stappert, S.; Li, C.; Müllen, K. Practical Syntheses of Terrylene Chromophores from Naphthalene and Perylene Building Blocks. *Adv. Synth. Catal.* **2017**, *359* (23), 4184–4189.
(2) Hayakawa, S.; Kawasaki, A.; Hong, Y.; Uraguchi, D.; Ooi, T.; Kim, D.; Akutagawa, T.; Fukui, N.; Shinokubo, H. Inserting Nitrogen: An Effective Concept to Create Nonplanar and Stimuli-Responsive Perylene Bisimide Analogues. *J. Am. Chem. Soc.* **2019**, *141* (50), 19807–19816.
(3) Gutiérrez-Abad, R.; Illa, O.; Ortuño, R. M. Synthesis of Chiral Cyclobutane Containing C 3 -Symmetric Peptide Dendrimers. *Org. Lett.* **2010**, *12* (14), 3148–3151.
(4) Krzeszewski, M. Synthesis of Tetraaryl-, Pentaaryl-, and Hexaaryl-1,4-Dihydropyrrolo[3,2-b]Pyrroles. *Org. Synth.* **2021**, *98*, 242–262.
(5) Wang, C.; Zheng, X.; Huang, R.; Yan, S.; Xie, X.; Tian, T.; Huang, S.; Weng, X.; Zhou, X. A 4-Amino-1,8-Naphthalimide Derivative for Selective Fluorescent Detection of Palladium(II) Ions. *Asian J. Org. Chem.* **2012**, *1* (3), 259–263.
(6) Frisch, M. J. *et al*. Gaussian 16, revision A.03; Gaussian Inc.: Wallingford, CT, 2016.
(7) Zhao, Y.; Truhlar, D. G. The M06 suite of density functionals for main group thermochemistry, thermochemical kinetics, noncovalent interactions, excited states, and transition elements: Two new functionals and systematic testing of four M06-class functionals and 12 other functionals. *Theor. Chem. Acc.,* 2008, **120**, 215−241.
(8) Tomasi, J.; Mennucci, B.; Cammi, R. Quantum Mechanical Continuum Solvation Models. *Chem. Rev*., 2005, **105**, 2999−3094.
(9) Guido, C. A.; Chrayteh, A.; Sclamani, G.; Mennucci, B.; Jacquemin, D. Reference Energies for Intramolecular Charge-Transfer Excitations. *J. Chem. Theory Comput.,* 2021, **17**, 5155−5164.
(10) Laurent, A. D.; Jacquemin, D. TD-DFT benchmarks: A review. *Int. J. Quantum Chem.,* 2013, **113**, 2019−2039.
(11) Christiansen, O.; Koch, H.; Jørgensen, P. The second-order approximate coupled cluster singles and doubles model CC2. *Chem. Phys. Lett.*, 1995, **243***,* 409−418.
(12) Hellweg, A.; Grün, S.; Hättig, C. Benchmarking the performance of spin-component scaled CC2 in ground and electronically excited states. *Phys. Chem. Chem. Phys.*, 2008, **10***,* 1159−1169.
(13) TURBOMOLE V7.3/V7.5, *a development of University of Karlsruhe and Forschungszentrum Karlsruhe GmbH*, 1989−2007; TURBOMOLE GmbH. http://www.turbomole.com.
(14) Jacquemin, D.; Duchemin, I.; Blase, X. 0–0 Energies Using Hybrid Schemes: Benchmarks of TD-DFT, CIS(D), ADC(2), CC2, and BSE/GW formalisms for 80 Real-Life Compounds. *J. Chem. Theory Comput.,* 2015, **11**, 5340−5359.
(15) Pershin, A.; Hall, D.; Lemaur, V.; Sanchi-Garcia, J. C.; Muccioli, L.; Zysman-Colman, E.; Beljonne, D.; Olivier, Y. Highly emissive excitons with reduced exchange energy in thermally activated delayed fluorescent molecules. *Nat. Commun.,* 2019, **10**, 597*.*
(16) Neese, F.; Wennmohs, F.; Becker, U.; Riplinger, C. The ORCA quantum chemistry program package. *J. Chem. Phys.,* 2020, **152**, 224108*.*
(17) Cerezo, J.; Santoro, F. FCClasses 3.0, http://www.pi.iccom.cnr.it/fcclasses
(18) Santoro, F.; Improta, R.; Lami, A.; Bloino, J.; Barone, V. Effective method to compute Franck-Condon integrals for optical spectra of large molecules in solution. *J. Chem. Phys.,* 2007, **126**, 084509
(19) Santoro, F.; Jacquemin, D. Going beyond the vertical approximation with time-dependent density functional theory. *Wires Comput. Mol. Sci*., 2016, **6**, 460–486.
(20) Peng, Q.; Yi, Y.; Shuai, Z. Excited state radiationless decay process with Duschinsky rotation effect: Formalism and implementation. *J. Chem. Phys*., 2007, **126**, 114302.
(21) Humemiuk, A.; Buzancic, M.; Hoche, J.; Cerezo, J.; Mitric, R.; Santoro, F.; Bonacic-Koutecey, V. Predicting fluorescence quantum yields for molecules in solution: A critical assessment of the harmonic approximation and the choice of the lineshape function. *J. Chem. Phys.,* 2020, **152**, 054107.
(22) Ou, Q.; Peng, Q.; Suhai, Z. Toward Quantitative Prediction of Fluorescence Quantum Efficiency by Combining Direct Vibrational Conversion and Surface Crossing: BODIPYs as an Example. *J. Phys. Chem. Lett.,* 2020, **11**, 7790–7797.
(23) Bajzar, L.; Nesheim, M. The effect of activated protein C on fibrinolysis in cell-free plasma can be attributed specifically to attenuation of prothrombin activation. *J. Biol. Chem.*, 1993, **268** (12), 8608–8616.
(24) Briet, E.; Noyes, C. M.; Roberts, H. R.; Griffith, M. J. Cleavage and activation of human prothrombin by Echis carinatus venom. *Thromb Res*., 1982, **27** (5), 591–600.
(25) Laemmli, U. K. Cleavage of structural proteins during the assembly of the head of bacteriophage T4. *Nature*, 1970, **227** (5259), 680–685.
(26) Sivaraman, A.; Kim, D. G.; Bhattarai, D.; Kim, M.; Lee, H. Y.; Lim, S.; Kong, J.; Goo, J.-il.; Shim, S.; Lee, S.; Suh, Y.-G.; Choi, Y.; Kim, S.; Lee, K. Synthesis and Structure–Activity Relationships of Arylsulfonamides as AIMP2-DX2 Inhibitors for the Development of a Novel Anticancer Therapy. *J. of Med. Chem.,* 2020, **63** (10), 5139–5158.
(27) Drąg, M. Parallel imaging of coagulation pathway proteases activated protein C, thrombin, and factor Xa in human plasma. *Chem. Sci.*, 2022, **13**, 6813–6829.
(28) Powers, J. C.; Asgian, J. L.; Ekici, O. D.; James, K. E. Irreversible inhibitors of serine, cysteine, and threonine proteases. *Chem Rev*, 2002, **102(12)**, 4639–4750.
